# Association of Preterm Birth and Socioeconomic Status With Neonatal Brain Structure

**DOI:** 10.1001/jamanetworkopen.2023.16067

**Published:** 2023-05-31

**Authors:** Katie Mckinnon, Paola Galdi, Manuel Blesa-Cábez, Gemma Sullivan, Kadi Vaher, Amy Corrigan, Jill Hall, Lorena Jiménez-Sánchez, Michael Thrippleton, Mark E. Bastin, Alan J. Quigley, Evdoxia Valavani, Athanasios Tsanas, Hilary Richardson, James P. Boardman

**Affiliations:** 1 Medical Research Council Centre for Reproductive Health, University of Edinburgh, Edinburgh, United Kingdom; 2 Centre for Clinical Brain Sciences, University of Edinburgh, Edinburgh, United Kingdom; 3 Department of Radiology, Royal Hospital for Children and Young People, Edinburgh, United Kingdom; 4 Usher Institute, Edinburgh Medical School, University of Edinburgh, Edinburgh, United Kingdom; 5 Alan Turing Institute, London, United Kingdom; 6 School of Philosophy, Psychology, and Language Sciences, University of Edinburgh, Edinburgh, United Kingdom

## Abstract

**Importance:**

Preterm birth and socioeconomic status (SES) are associated with brain structure in childhood, but the relative contributions of each during the neonatal period are unknown.

**Objective:**

To investigate associations of birth gestational age (GA) and SES with neonatal brain morphology by testing 3 hypotheses: GA and SES are associated with brain morphology; associations between SES and brain morphology vary with GA; and associations between SES and brain structure and morphology depend on how SES is operationalized.

**Design, Setting, and Participants:**

This cohort study recruited participants from November 2016 to September 2021 at a single center in the United Kingdom. Participants were 170 extremely and very preterm infants and 91 full-term or near-term infants. Exclusion criteria were major congenital malformation, chromosomal abnormality, congenital infection, cystic periventricular leukomalacia, hemorrhagic parenchymal infarction, and posthemorrhagic ventricular dilatation.

**Exposures:**

Birth GA and SES, operationalized at the neighborhood level (using the Scottish Index of Multiple Deprivation), the family level (using parental education and occupation), and subjectively (World Health Organization Quality of Life measure).

**Main Outcomes and Measures:**

Brain volume (85 parcels) and 5 whole-brain cortical morphology measures (gyrification index, thickness, sulcal depth, curvature, surface area) at term-equivalent age (median [range] age, 40 weeks, 5 days [36 weeks, 2 days to 45 weeks, 6 days] and 42 weeks [38 weeks, 2 days to 46 weeks, 1 day] for preterm and full-term infants, respectively).

**Results:**

Participants were 170 extremely and very preterm infants (95 [55.9%] male; 4 of 166 [2.4%] Asian, 145 of 166 [87.3%] White) and 91 full-term or near-term infants (50 [54.9%] male; 3 of 86 [3.5%] Asian, 78 of 86 [90.7%] White infants) with median (range) birth GAs of 30 weeks, 0 days (22 weeks, 1 day, to 32 weeks, 6 days) and 39 weeks, 4 days (36 weeks, 3 days, to 42 weeks, 1 day), respectively. In fully adjusted models, birth GA was associated with a higher proportion of brain volumes (27 of 85 parcels [31.8%]; β range, −0.20 to 0.24) than neighborhood-level SES (1 of 85 parcels [1.2%]; β = 0.17 [95% CI, −0.16 to 0.50]) or family-level SES (maternal education: 4 of 85 parcels [4.7%]; β range, 0.09 to 0.15; maternal occupation: 1 of 85 parcels [1.2%]; β = 0.06 [95% CI, 0.02 to 0.11] respectively). There were interactions between GA and both family-level and subjective SES measures on regional brain volumes. Birth GA was associated with cortical surface area (β = 0.10 [95% CI, 0.02 to 0.18]) and gyrification index (β = 0.16 [95% CI, 0.07 to 0.25]); no SES measure was associated with cortical measures.

**Conclusions and Relevance:**

In this cohort study of UK infants, birth GA and SES were associated with neonatal brain morphology, but low GA had more widely distributed associations with neonatal brain structure than SES. Further work is warranted to elucidate the mechanisms underlying the association of both GA and SES with early brain development.

## Key Points

**Question:**

Are preterm birth, socioeconomic status (SES), and neonatal brain structure associated?

**Findings:**

In this cohort study of 261 infants, after mutual adjustment, both low birth gestational age (GA) and SES were associated with brain structure. The nature of SES–brain structure associations varied depending how SES was operationalized; there were interactions between GA and measures of family-level SES on brain structure.

**Meaning:**

In this study, low birth GA, and to a lesser extent SES, were associated with neonatal brain structure; further work is required to elucidate potential mechanisms underlying this association.

## Teaser

This cohort study investigates associations of gestational age at birth and socioeconomic status with neonatal brain morphology in a cohort of preterm and full-term infants in the United Kingdom.

## Introduction

Preterm birth, defined as birth at less than 37 weeks of gestation, affects 11% of births globally and is a leading cause of atypical brain development, underpinning long-term motor, cognitive, and behavioral problems.^1,2^ There is a dose-dependent association between low gestational age (GA) and increased likelihood of difficulties, with those born extremely (<28 weeks) and very (28 to 32 weeks) preterm being at greatest risk for cerebral palsy, cognitive impairment, lower educational attainment, visual or hearing impairment, attention-deficit/hyperactivity disorder, autism spectrum disorder, and mental health diagnoses across the life course.^2,3,4^

Preterm birth is associated with structural brain changes apparent by term-equivalent age, including global and regional tissue volume reduction, altered cortical configuration, and enlargement of cerebrospinal fluid (CSF) spaces, although smaller tissue volumes are not inevitable.^5,6,7,8^ Changes in neonatal morphology are associated with later functional impairment, highlighting the importance of elucidating factors contributing to early brain development.^9,10,11,12^

In the general population, brain structure, cognition, educational attainment, and adult income are patterned by socioeconomic status (SES) in childhood.^13^ Children living in poverty are more likely to experience difficulties with memory, language, self-regulation, and socioemotional processing and are more likely to receive behavioral and mental health diagnoses.^14,15,16,17,18,19,20,21^ Indeed, mediation analyses suggest a causal pathway between SES and cognitive development in childhood via changes in brain anatomy and function.^13,22,23,24,25^ SES is inherently a multifaceted construct and can be operationalized using neighborhood-level, family-level, or subjective measures.^14,21^ These capture different phenomena, and the extent to which they correlate with one another depends on setting and population. Understanding the relative associations of socioeconomic disadvantage, operationalized in different ways, and low GA with neonatal brain development is important for designing rational therapies and support strategies for children born preterm.

To investigate associations between birth GA and SES with neonatal brain development, we combined high-resolution brain magnetic resonance imaging (MRI) from infants born extremely preterm, very preterm, and at full term, with neighborhood-level, family-level, and subjective SES measures. We investigated associations of GA and SES with brain structure by testing hypotheses that (1) GA and SES are associated with neonatal brain structure in mutually adjusted models; (2) associations between SES and brain structure vary with GA at birth; and (3) associations between SES and brain morphology depend on how SES is operationalized.

## Methods

### Study Participants

Participants were extremely or very preterm infants (birth <33 weeks’ gestation [n = 170]) and full-term or near-term control infants (n = 91), recruited to a longitudinal study investigating the association of preterm birth with brain development and outcomes^26^ (eFigure 1 in Supplement 1). The cohort is focused on very and extremely preterm infants due to their increased likelihood of atypical brain development.^2,4^

Exclusion criteria were major congenital malformation, chromosomal abnormality, congenital infection, cystic periventricular leukomalacia, hemorrhagic parenchymal infarction, and posthemorrhagic ventricular dilatation. We excluded infants with significant parenchymal brain injury to be representative of most survivors of modern intensive care practices.^27^

Recruitment was conducted at the Royal Infirmary of Edinburgh, United Kingdom, between November 2016 and September 2021. Ethical approval was obtained from the UK National Research Ethics Service, and parents provided written consent. As per COVID-19 policies, recruitment and MRI scans were paused from March to June 2020. This study followed the Strengthening the Reporting of Observational Studies in Epidemiology (STROBE) reporting guideline.

### Demographic Variables

We assigned preterm infants into 4 categories with similar numbers of participants based on birth GA: 22 weeks, 0 days, to 26 weeks, 6 days; 27 weeks, 0 days, to 28 weeks, 6 days; 29 weeks, 0 days, to 30 weeks, 6 days; and 31 weeks, 0 days, to 32 weeks, 6 days, of gestation. SES was measured in 3 ways: neighborhood-level, defined by the Scottish Index of Multiple Deprivation 2016 (SIMD), derived from the family’s postal code at birth^28^; family-level, defined as parental education (highest educational qualification and age leaving education) and parental occupation (current or most recent job); and subjective SES provided by parental self-report using the environment domain of the World Health Organization Quality of Life (WHO QoL) assessment.^29^ SIMD rank was chosen as the primary measure of SES because it correlates with child development^30^ and is a tractable tool for policy makers.^28^ Other SES measures were investigated in exploratory analyses, as specified in our preregistered statistical plan^31^: maternal and paternal education, maternal and paternal occupation, and subjective SES (eMethods in Supplement 1). Ethnicity data were collected, as there are relationships between ethnicity and SES.^32^ Ethnicity (Asian, Black, mixed ethnicity, White, and other ethnic group [Arab, Iraqi, Bulgarian/Turkish, and Fijian]) was determined by self-report through questionnaire (eMethods in Supplement 1).

### Selection of Covariates

Covariate selection was based on associations with brain structure in prior research: birth weight *z* score,^33,34^ birth head circumference *z* score,^35^ age at MRI, infant sex,^36^ smoking in pregnancy,^37,38^ and any breast milk at discharge.^39,40^ Definitions appear in the eMethods in Supplement 1, and the conceptual model appears in eFigure 2 in Supplement 1.

### MRI Data Acquisition

MRI scans were performed at term-corrected gestation according to a published protocol.^26^ In summary, a MAGNETOM Prisma 3T MRI clinical scanner (Siemens Healthcare) and 16-channel phased-array pediatric head receive coil were used to acquire a 3-dimensional T1-weighted magnetization-prepared rapid acquisition with gradient echo structural volume scan (voxel size, 1 mm isotropic); and a 3-dimensional T2-weighted (T2w) sampling scheme with application-optimized contrasts using flip angle evolution structural scan (voxel size, 1 mm isotropic).

Infants were fed, wrapped, and slept naturally. Flexible earplugs and neonatal earmuffs (MiniMuffs) were used for acoustic protection. Infants were monitored throughout, and scans were supervised by a doctor or nurse trained in neonatal resuscitation.

### MRI Data Analysis

Structural images were reported by a radiologist with experience in neonatal MRI (A.J.Q.). The developing Human Connectome Project (dHCP) minimal processing pipeline for neonatal data^41^ was used to preprocess T2w images, allowing surface reconstruction from tissue segmentation. We obtained bias field–corrected T2w, brain masks, tissue segmentation, label parcellation, and surface reconstruction. We then calculated tissue volumes, gyrification index (GI), cortical thickness, sulcal depth, cortical curvature, and cortical surface area (SA).^41^ Visual inspection and quality control were performed by experienced neuroscientists (M.B.-C. and K.V.).

### Selection of Image Features

We included 85 individual regional brain parcels (including white matter, gray matter, and CSF spaces) and 5 whole-cortex measures (GI, thickness, sulcal depth, curvature, and SA), as defined by the dHCP.^41^ This was due to previous literature providing evidence for regional volumetric changes in association with SES in childhood.^31,42,43,44,45,46,47,48,49^ Based on reports of SES–brain structure correlations when the brain is characterized using larger parcellations, we analyzed associations of GA and SES with whole brain volume, lobar regional volumes, and lobar cortical measures.

### Statistical Analysis

Statistical analyses were preregistered^31^ and conducted in R version 4.2.1 (R Project for Statistical Computing). We compared demographic data and SES measures across preterm and full-term groups. We compared categorical variables using χ^2^ tests (significance threshold, *P* < .05). For continuous variables, we used Mann-Whitney *U* tests. We assessed statistical relationships between SES measures using Spearman correlation, with strength of correlation classified as very weak (*r* = 0-0.19), weak (*r* = 0.20-0.39), moderate (*r* = 0.40-0.59), strong (*r* = 0.60-0.79), or very strong (*r* = 0.80-1).

Because the sample included twins (25 pairs) and siblings (5 groups), which violates the assumption of nonindependence among data points, we repeated analyses after random removal of all but 1 sibling or twin per family. To investigate associations between SES and birth GA with regional brain volumes and cortical measures, we developed regression models. Model 1 was a baseline linear regression model, including GA at birth, SES (neighborhood-level, family-level, or subjective), gestation at MRI, and a product interaction term (GA at birth × SES, if significant). Model 2 was a linear ridge regression model^50^ including all covariates: GA at birth, SES (neighborhood-level, family-level, or subjective), gestation at MRI, the product interaction term (if significant), birth weight *z* score, birth head circumference *z* score, sex, smoking in pregnancy, and breast milk at discharge. Ridge regression aims to partially mitigate potential multicollinearity among factors. We ran 85 regression models testing the association between each SES measure and GA and each regional brain volume, and 5 additional regression models testing the association between each SES measure and GA and each whole-brain cortical measure. Results are reported as standardized β values, classified as small (β < 0.20), medium (β = 0.20-0.49), and large (β ≥ 0.50), with 95% CIs. To correct for multiple comparisons for each measure, we used Benjamini-Hochberg correction^51^ across the 85 parcels for each SES measure at adjusted and unadjusted levels separately, with the threshold of statistical significance set at *P* < .05. We compared the frequency of brain volume associations with GA and SES variables using McNemar tests.

As described in the statistical analysis plan,^31^ SIMD was the primary SES measure in analyses, and maternal and paternal education, maternal and paternal occupation, and subjective SES were investigated in exploratory analyses. For these, the same statistical thresholds used in the primary analyses were applied.

## Results

### Participant Characteristics

Participants were 170 extremely and very preterm (95 [55.9%] male; 4 of 166 [2.4%] Asian and 145 of 166 [87.3%] White) and 91 full-term or near-term (50 [54.9%] male; 3 of 86 [3.5%] Asian, 76 of 86 [90.7%] White) infants, with median (range) birth GAs of 30 weeks, 0 days (22 weeks, 1 day, to 32 weeks, 6 days) and 39 weeks, 4 days (36 weeks, 3 days, to 42 weeks, 1 day), respectively; their demographic characteristics are shown in Table 1. There was no difference in sex distribution across groups. Ethnicity did not differ between groups and is representative of Edinburgh.^52^ All SES measures, smoking prevalence, and multiple pregnancy differed between groups, and the SES measure spread is representative of Edinburgh.^52,53^

**Table 1.** Participant Characteristics

Characteristic	Participants, No./total No. (%)^a^		*P* value^b^
	Preterm (n = 170)	Term (n = 91)	
Gestational age at birth, median (range), wk + d	30 + 0 (22 + 1 to 32 + 6)	39 + 4 (36 + 3 to 42 + 1)	<.001
Gestational age at MRI, median (range), wk + d	40 + 5 (36 + 2 to 45 + 6)	42 + 0 (38 + 2 to 46 + 1)	<.001
Birth weight, median (range), g	1315 (370 to 2510)	3460 (2410 to 4560)	<.001
Birthweight *z* score, median (range)	0.13 (−3.13 to 2.14)	0.45 (−2.30 to 2.57)	<.001
Head circumference, median (range), cm^c^	27.5 (17.5 to 33.8)	35 (32 to 39)	<.001
Head circumference *z* score, median (range)^c^	−0.12 (−3.13 to 5.31)	0.99 (−1.54 to 3.73)	<.001
Sex			
Male	95/170 (55.9)	50/91 (54.9)	.21
Female	75/170 (44.1)	41/91 (45.1)	
Maternal smoking during pregnancy	30/167 (18.0)	3/91 (3.3)	<.001
Multiple pregnancy	51/170 (30.0)	2/91 (2.2)	<.001
Any breast milk at discharge	122/167 (73.1)	85/91 (93.4)	<.001
Child ethnicity			
Asian	4/166 (2.4)	3/86 (3.5)	.22
Black	2/166 (1.2)	0/86	
Mixed ethnicity	9/166 (5.4)	5/86 (5.8)	
White	145/166 (87.3)	76/86 (90.7)	
Other ethnic group^d^	5/166 (3.0)	0/86	
SIMD rank, median (range)	3913 (6-6929)	5502 (727-6967)	<.001
Mother age leaving education, median (range), y^e^	20 (14-33)	23 (16-36)	<.001
Father age leaving education, median (range), y^f^	18 (14-34)	22 (16-36)	<.001
Mother highest educational qualification			
None	5/165 (3.0)	0/91	<.001
Basic high school qualification	18/165 (10.9)	5/91 (5.5)	
Advanced high school qualification	13/165 (7.9)	2/91 (2.2)	
College qualification	42/165 (25.5)	9/91 (9.9)	
University undergraduate degree	44/165 (26.7)	40/91 (44.0)	
University postgraduate degree	34/165 (20.6)	35/91 (38.5)	
Not applicable	9/165 (5.5)	0/91	
Father highest educational qualification			
None	4/148 (2.7)	0/90	<.001
Basic high school qualification	33/148 (22.3)	7/90 (7.8)	
Advanced high school qualification	13/148 (8.8)	5/90 (5.6)	
College qualification	33/148 (22.3)	12/90 (13.3)	
University undergraduate degree	33/148 (22.3)	41/90 (45.6)	
University postgraduate degree	22/148 (14.9)	25/90 (27.8)	
Not applicable	10/148 (6.8)	0/90	
Mother current or recent job			
Professional	75/166 (45.2)	76/91 (83.5)	<.001
Nonmanual skilled	32/166 (19.3)	6/91 (6.6)	
Manual skilled	16/166 (9.6)	1/91 (1.1)	
Partly skilled	8/166 (4.8)	4/91 (4.4)	
Unskilled	14/166 (8.4)	2/91 (2.2)	
Unemployed	3/166 (1.8)	1/91 (1.1)	
Homemaker	10/166 (6.0)	0/91	
Still in full-time education	7/166 (4.2)	1/91 (1.1)	
Father current or recent job			
Professional	59/157 (37.6)	63/91 (69.2)	<.001
Nonmanual skilled	23/157 (14.6)	6/91 (6.6)	
Manual skilled	37/157 (23.6)	7/91 (7.7)	
Partly skilled	22/157 (14.0)	6/91 (6.6)	
Unskilled	6/157 (3.8)	3/91 (3.3)	
Unemployed	3/157 (1.9)	0/91	
Homemaker	3/157 (1.9)	0/91	
Still in full time education	3/157 (1.9)	6/91 (6.6)	
WHO QoL environment score, median (range)	75.0 (21.9 to 100.0)	84.4 (40.6 to 100.0)	<.001

Abbreviations: MRI, magnetic resonance imaging; SIMD, Scottish Index of Multiple Deprivation; WHO QoL, World Health Organization Quality of Life assessment.

^a^ Case definitions are available in eMethods in Supplement 1.

^b^ For binary data, the χ^2^ test was used for *P* values. For continuous data, Mann-Whitney *U* test was used.

^c^ For head circumference and head circumference *z* score, there were data for 157 preterm infants and 83 full-term infants.

^d^ This included Arab, Iraqi, Bulgarian/Turkish, and Fijian.

^e^ For maternal age leaving education, data were available for 158 preterm and 85 full-term infants.

^f^ For partner age leaving education, data were available for 139 preterm and 79 full-term infants.

There were no significant demographic differences between included and excluded infants (eTable 1 in Supplement 1). The major exposures and comorbidities of the preterm group are shown in eTable 2 in Supplement 1. Removal of all but 1 sibling per family from the sample and rerunning analyses yielded a similar pattern of results, so the whole sample is included in reported analyses.

### Associations Between Regional Brain Volumes, Prematurity, and Neighborhood Deprivation

Birth GA was associated with more regional brain volumes than SIMD (McNemar test comparing frequency of associations: *P* < .001) (Table 2; eTable 3 in Supplement 1). After Benjamini-Hochberg correction, GA correlated with the volume of 22 of 85 parcels (26%) (range: subthalamic nucleus left, β = −0.13 [95% CI, −0.23 to −0.03] to medial and inferior temporal gyri anterior part right gray matter, β = 0.22 [95% CI, 0.16 to 0.29]) compared with 1 of 85 (1.2%) for SIMD (medial and inferior temporal gyri anterior part right white matter: β = 0.17 [95% CI, −0.16 to 0.50]). GA-associated parcels were within gray and white matter, often bilaterally, and predominantly had a positive association (17 of 19 tissue [non-CSF] regions [89.5%]) meaning that higher GA at birth was associated with increased tissue volume. There were negative associations between GA and volumes of CSF spaces (bilateral lateral ventricles and extracerebral CSF). There were no interactions between GA and SIMD for any brain region.

**Table 2.** Regional Brain Volumes With Significant Associations With Either Gestational Age or the SIMD^a^

Regional volume	Standardized β coefficient (95% CI)	*P* value	
		Raw	BH corrected
Gestational age			
Anterior temporal lobe lateral part left gray matter	0.14 (0.06 to 0.23)	<.001	.003
Caudate nucleus left	0.16 (0.07 to 0.25)	<.001	.001
Caudate nucleus right	0.13 (0.04 to 0.22)	<.001	.01
Cerebrospinal fluid	−0.11 (−0.21 to −0.005)	<.001	<.001
Cingulate gyrus posterior part right gray matter	−0.12 (−0.21 to −0.02)	.01	.047
Frontal lobe left white matter	0.20 (0.12 to 0.28)	<.001	<.001
Frontal lobe right white matter	0.16 (0.08 to 0.25)	<.001	<.001
Gyri parahippocampalis et ambiens anterior part left white matter	0.16 (0.06 to 0.26)	<.001	.001
Gyri parahippocampalis et ambiens posterior part right white matter	0.14 (0.04 to 0.24)	<.001	.01
Insula left white matter	0.17 (0.08 to 0.26)	<.001	<.001
Insula right white matter	0.16 (0.08 to 0.25)	<.001	.001
Lateral occipitotemporal gyrus gyrus fusiformis anterior part left white matter	0.20 (0.12 to 0.29)	<.001	<.001
Lateral occipitotemporal gyrus gyrus fusiformis anterior part right gray matter	0.15 (0.07 to 0.24)	<.001	.001
Lateral ventricle left	−0.12 (−0.23 to −0.02)	<.001	.001
Lateral ventricle right	−0.10 (−0.20 to 0.004)	<.001	.002
Medial and inferior temporal gyri anterior part left gray matter	0.22 (0.15 to 0.29)	<.001	<.001
Medial and inferior temporal gyri anterior part right gray matter	0.22 (0.16 to 0.29)	<.001	<.001
Medial and inferior temporal gyri posterior part left gray matter	0.11 (0.05 to 0.18)	<.001	.005
Medial and inferior temporal gyri posterior part right gray matter	0.14 (0.07 to 0.20)	<.001	.001
Parietal lobe right gray matter	0.09 (0.03 to 0.15)	.001	.01
Subthalamic nucleus left	−0.13 (−0.23 to −0.03)	.003	.03
Superior temporal gyrus middle part right gray matter	0.11 (0.0 to 0.18)	.001	.01
SIMD			
Medial and inferior temporal gyri anterior part right white matter	0.17 (−0.16 to 0.50)	.004	.03

Abbreviations: BH, Benjamini Hochberg correction; SIMD, Scottish Index of Multiple Deprivation.

^a^ Fully adjusted ridge regression model, including gestation at birth, SIMD, gestation at magnetic resonance imaging scan, the interaction term (when significant), birth weight *z* score, birth head circumference *z* score, sex, smoking in pregnancy, and breast milk at discharge. Corrected for false discovery rate. Results for all parcels are available in eTable 3 in Supplement 1.

Fully adjusted models showed a similar profile of results to baseline models (eTable 4 in Supplement 1), with GA-parcel volume associations more widely distributed than SIMD-parcel volume associations. The number of associations was higher for GA than SIMD: 50 of 85 (58.8%) and 5 of 85 (5.9%), respectively.

### Associations Between Global Cortical Measures, Prematurity, and Neighborhood Deprivation

Birth GA was associated with cortical SA (β = 0.10 [95% CI, 0.02-0.18]; *P* = .03) and GI (β = 0.16 [95% CI, 0.07-0.25]; *P* < .001), whereas SIMD was not associated with any measure of cortical morphology (Table 3). There were no interactions between GA and SIMD for any global cortical measure.

**Table 3.** Association of Cortical Measures With Gestational Age and the SIMD^a^

Cortical measure	Standardized β coefficient (95% CI)	*P* value	
		Raw	BH corrected
**Gestational age**			
Mean cortical curvature	0.01 (−0.07 to 0.09)	.75	.75
Mean cortical surface area	0.10 (0.02 to 0.18)	.01	.03
Mean cortical thickness	−0.03 (−0.12 to 0.06)	.43	.48
Mean gyrification index	0.16 (0.07 to 0.25)	<.001	<.001
Mean sulcal depth	0.03 (−0.08-0.14)	.04	.15
**SIMD**			
Mean cortical curvature	−0.05 (−0.15 to 0.06)	.37	.46
Mean cortical surface area	0.05 (−0.05 to 0.16)	.29	.46
Mean cortical thickness	−0.05 (−0.18 to 0.08)	.36	.46
Mean gyrification index	0.07 (−0.05 to 0.19)	.20	.40
Mean sulcal depth	0.02 (−0.12 to 0.17)	.19	.40

Abbreviations: BH, Benjamini Hochberg correction; SIMD, Scottish Index of Multiple Deprivation.

^a^ Fully adjusted ridge regression model, including gestation at birth, SIMD, gestation at magnetic resonance imaging scan, the interaction term (if applicable), birth weight *z* score, birth head circumference *z* score, sex, smoking in pregnancy, and breast milk at discharge. Corrected for false discovery rate.

Fully adjusted models revealed a similar profile of results to the baseline linear regression models. There were GA associations in 2 of 5 cortical measures (40.0%) and no associations with SIMD (eTable 5 in Supplement 1).

### Correlations Between Neighborhood-Level, Family-Level, and Subjective SES Measures

Figure, A shows that correlations between SES measures ranged from very weak (eg, between age of father leaving education and the WHO QoL environment score, *r* = 0.07) to strong (eg, age of father and mother leaving education, *r* = 0.70; final education qualification, *r* = 0.64). Correlations between SIMD rank and parental education and occupation were weak to moderate (*r* = 0.22-0.40).

**Figure.**
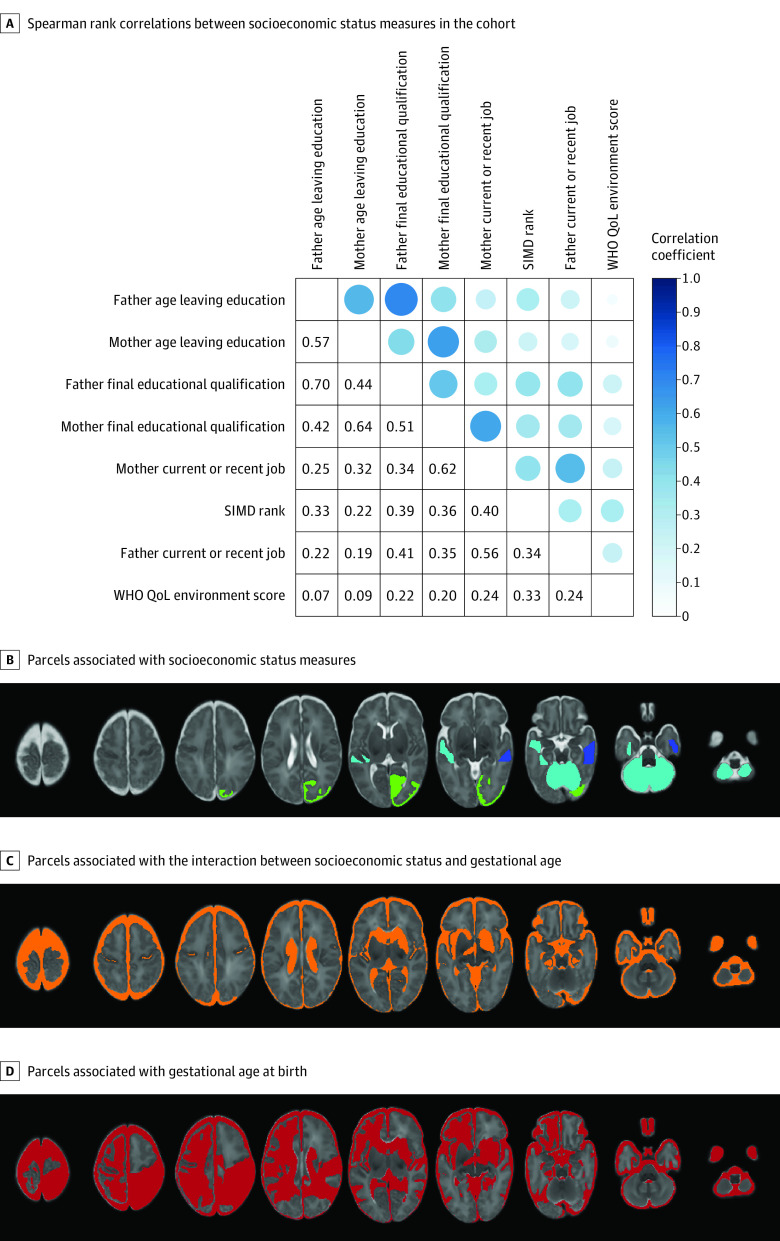
Socioeconomic Status Measures A, Coefficients are shown when *P* < .05. B-D, Panels show all regions and parcels that were significantly associated with respective measures after Benjamini-Hochberg correction in fully adjusted ridge regression models. B, Blue indicates SIMD (1 parcel); turquoise, maternal education (4 parcels); green, maternal occupation (1 parcel). C, Orange indicates socioeconomic status × gestational age interaction (12 parcels). D, Red indicates gestational age at birth (27 parcels).

### Associations Between Brain Features and Family-Level and Subjective SES Measures

In exploratory analyses defining SES by parental education and occupation and subjective measures, a similar pattern of results was obtained as with SIMD: a greater proportion of brain volumes were associated with birth GA than SES (McNemar test comparing frequency of associations: *P* < .001 for all SES measures) (Table 4; eTables 6-10 in Supplement 1; Figure, B-D). However, there were differences in associations between SES and brain structure depending on the SES measure used. Specifically, maternal education was associated with the volume of 4 parcels (left and right cerebella, left middle superior temporal gyrus, and left anterior lateral occipitotemporal gyrus/gyrus fusiformis; β range, 0.09 to 0.15) (eTable 6 in Supplement 1), whereas neighborhood SES (Table 2) and maternal occupation (eTable 8 in Supplement 1) were each associated with 1 parcel volume: right anterior medial and inferior temporal gyri (white matter) (β = 0.17 [95% CI, −0.16 to 0.50], *P* = .03), and right occipital lobe (gray matter) (β = 0.06 [95% CI, 0.02 to 0.11]; *P* = .049), respectively. Associations between parcel volumes and family-level SES were positive, meaning that higher family-level SES was associated with increased regional tissue volume.

**Table 4.** Summary of Associations Between GA and Neighborhood-Level, Family-Level, and Subjective Measures of SES With Brain Features^a^

Brain Features	Parcels, No./total No. (%)		
	GA	SES	Interaction effect
**Proportion of brain volumes associated with GA and SES**			
SIMD	22/85 (25.9)	1/85 (1.2)	0/85
Maternal education	15/85 (17.6)	4/85 (4.7)	5/85 (5.9)
Paternal education	22/85 (25.9)	0/85	7/85 (8.2)
Maternal occupation	12/85 (14.1)	1/85 (1.2)	10/85 (11.8)
Paternal occupation	21/85 (24.7)	0/85	5/85 (5.9)
Subjective socioeconomic status	17/85 (20.0)	0/85	4/85 (4.7)
**Proportion of cortical features associated with GA and SES**			
SIMD	2/5 (40.0)	0/5	0/5
Maternal education	0/5	0/5	0/5
Paternal education	0/5	0/5	0/5
Maternal occupation	0/5	0/5	0/5
Paternal occupation	0/5	0/5	0/5
Subjective socioeconomic status	1/5 (20.0)	0/5	0/5

Abbreviations: GA, gestational age; SES, socioeconomic status; SIMD, Scottish Index of Multiple Deprivation.

^a^ Results summary from ridge regression models for each socioeconomic status measure, for the 85 regional brain volumes and 5 mean cortical measures. Significance threshold is *P* < .05 following Benjamini-Hochberg correction. Full results are available in Table 3; eTables 3 and 6 to 15 in Supplement 1.

GA-volume associations for paternal education and occupation were found in 22 of 85 parcels (25.9%) and 21 of 85 parcels (24.7%), respectively. For maternal education and occupation, GA-volume associations were found in 15 of 85 parcels (17.6%) and 12 of 85 parcels (14.1%), respectively. Associations were observed for subjective SES in 17 of 85 parcels (20.0%) (Table 4).

In contrast to SIMD, there were significant interactions between family-level and subjective SES measures and GA for several brain structures (eTables 6-15 in Supplement 1; Figure, C). For family-level and subjective SES measures, the association with SES was smaller in children with higher GA for extracerebral CSF and left and right lateral ventricular volumes (β range, −0.05 to −0.001). The direction of the interaction varied for those involving white and gray tissue parcels, such as left anterior gyri parahippocampalis et ambiens (white matter), left caudate nucleus, and left subthalamic nucleus. For the 5 global cortical measures (eTables 11-15 in Supplement 1), there were no associations with family-level or subjective SES in the fully adjusted model.

### Associations Between GA and SES Measures With Whole Brain and Lobar Volumes and Lobar Cortical Measures

GA was associated with whole brain volume (β range, 0.05-0.09), left frontal lobe SA (β range, 0.14-0.19), right frontal lobe volume (β range, 0.10-0.13) and SA (β range, 0.13-0.18), left parietal lobe volume (β range, 0.09-0.11) and SA (β range, 0.14-0.17), right parietal lobe volume (β range, 0.09-0.10) and SA (β range, 0.10-0.12), left temporal lobe volume (β range, 0.10-0.11) and SA (β range 0.11-0.14), right temporal lobe volume (β range, 0.09-0.10) and SA (β range, 0.09-0.13), and left occipital lobe thickness (β = −0.02) (eTables 16-27 in Supplement 1). Maternal education was associated with right frontal lobe sulcal depth (β = 0.14), and paternal occupation was associated with left occipital lobe GI (β = −0.09) (eTables 16-27 in Supplement 1).

There was an interaction between GA and maternal education in association with left and right frontal lobe thickness (β = −0.02). An interaction with GA and maternal occupation was seen with left and right frontal lobe thickness (β = −0.01) and left occipital lobe curvature (β = −0.01) and GI (β = −0.01). There was an interaction between GA and paternal education in association with left frontal lobe thickness (β = −0.01) and between GA and SIMD with right parietal lobe sulcal depth (β = −0.03) (eTables 16-27 in Supplement 1).

## Discussion

In a high-income setting, low GA at birth and SES were both associated with regional differences in brain structure that were apparent at the end of neonatal care. Low GA was associated with widely distributed differences in brain structure and cortical morphology, whereas differences associated with SES were less widely distributed. Standardized β values showed small to medium associations between brain structure and low GA and small associations with SES measures. We found that neighborhood-level, family-level, and subjective measures of SES were only weakly to moderately correlated; of these, family-level measures (parental education and occupation) were associated with more differences in brain structure than subjective SES and neighborhood deprivation. Furthermore, there was an interaction between low GA and family-level and subjective SES measures. These results suggest that atypical brain development seen in preterm infants is associated predominantly with GA at birth, but prematurity does not override SES-brain structure patterning, so interventions designed to attenuate family-level socioeconomic disadvantage in the perinatal period^54^ could promote healthier brain development in preterm infants.

Our results are consistent with studies suggesting that SES may modify the association between preterm birth and neurodevelopmental and educational outcomes, especially in the context of preterm brain injury.^55,56,57,58,59^ Although few studies have reported the impact of SES on brain morphology of preterm infants, there is some consensus that socioeconomic factors play a role. In a large study of US infants with GA range of 27 to 42 weeks who were scanned between term and age 4 months, higher SES, indexed by parental education, had marginal associations with brain volume in infancy, and paternal education was associated with gray matter volume after accounting for birth weight. However, extremely preterm infants were not represented in this study, and findings could have been confounded by post–neonatal intensive care unit (NICU) exposures.^57^ In an Australian cohort, a multidimensional measure of social risk was associated with global and regional brain volumes in a group of preterm infants at term-equivalent age, but the strength of association varied by GA category (strongest for late preterm [34-36 weeks of gestation] and full-term infants compared with moderate or very preterm infants [<34 weeks of gestation]) and was diminished in multivariable models adjusting for poor intrauterine growth, multiple birth, and male sex.^59^ Our results indicate that although low GA was associated with the widest distribution of parcels, SES associations were detectable at the end of NICU care after adjustment for GA at birth, age at MRI, birth weight *z* score, birth head circumference *z* score, sex, smoking in pregnancy, and nutrition during NICU care.

The regional volumes associated with low birth GA are in white and gray matter bilaterally, with concurrent increases in extra-axial and lateral ventricular CSF spaces. This pattern is consistent with the cerebral signature of preterm birth, which includes altered cortical morphology, focal white matter volume loss, and reduced deep gray matter volume.^5^ By using a contemporary atlas,^60^ we have added anatomic granularity to the preterm brain structural phenotype. In adjusted models, regions associated with SES were right anterior medial and inferior temporal gyri (white matter) (SIMD); right occipital lobe (gray matter) (maternal occupation); left and right cerebella, left middle superior temporal gyrus, and left anterior lateral occipitotemporal gyrus/gyrus fusiformis (maternal education). SES measures have previously shown variable associations in childhood with the cerebellum,^25,45,56^ occipital lobe,^25,61^ and temporal lobe,^24,25,61^ and the middle temporal gyri and occipitotemporal regions specifically have been positively associated with a composite SES of maternal education and occupation.^49^ The possible functions of these SES-associated regions include memory, visual information processing, speech, motor control, balance, and cognition.^62^

Most studies examining associations between SES and brain morphology have used individual or family measures of SES (parental education or occupation, or family income)^46,56,57,58,61,63,64^ or a composite,^59,65^ and only 1 included neighborhood deprivation.^65^ We found that family-level SES measures were most closely associated with brain structure. This could be explained by family-level measures capturing shared genetic determinants of brain anatomy and exposures to stress, nutrition, or smoking that influence brain development,^38,66,67^ which are not fully captured by neighborhood deprivation. Neighborhood-level SES is associated with functional changes in later infancy within this cohort, including preference to view social stimuli^68^ and emotional regulation and cortisol response,^69^ suggesting that neighborhood deprivation could have a greater impact after discharge from hospital.

### Strengths and Limitations

The study has the following strengths. First, the preterm infants did not have focal parenchymal brain injuries so are representative of most survivors of modern intensive care in high-income countries. Second, we assessed whole brain anatomy and global cortical structure using an open-source age-specific atlas. Third, we adjusted for real and potential variables associated with neonatal brain structure and used ridge regression to mitigate the problem of multicollinearity. Fourth, we explored SES-brain associations with SES operationalized at different levels, showing that family and subjective measures interact with the associations of GA with the brain. Finally, we used a preregistered analysis plan to reduce the risk of false-positive results.

The study also has some limitations. Although the study population was comparable with neonatal populations in high-income, majority-White settings, the results may not be generalizable to settings with different socioeconomic or ethnicity profiles. We studied a range of measures of SES but did not include all that could be relevant, such as household income. Our preterm cohort was designed to assess only those born extremely or very preterm; further work would be required to determine whether the associations we observed are present in moderate to late preterm infants.^26^ Our choice to study morphology was based on existing literature. We did not investigate possible SES associations with structural or functional connectivity, or other metrics of early brain development such as network complexity,^70^ brain age,^71^ voxel-based morphometry,^72^ tensor-based morphometry,^73^ or relative brain volumes; future research could investigate whether these techniques uncover additional insight about SES and preterm brain development. SES and preterm birth were correlated (Table 1), which makes the disambiguation of effects challenging. The statistical mitigations we used to address this issue included ridge regression to reduce impacts of colinearity and inclusion of interaction terms (GA × SES) in models. The results imply that GA leads to more widely distributed associations across the brain than SES. However, associations with SES might be underestimated, for example, if atypical fetal brain development begins in utero, before the event of preterm birth, for some women living in deprived circumstances. Prospective fetal studies using MRI and/or large-scale epidemiological studies that include neurobehavioral outcomes could provide additional information about the role of social determinants on the outcomes of children born preterm. A larger sample would be required to investigate whether comorbidities of preterm birth, such as sepsis, necrotizing enterocolitis or bronchopulmonary dysplasia, modify the brain-SES associations we observed.

We cannot comment on whether SES patterning of brain structure is dynamic through childhood after preterm birth. The consensus for associations between SES and brain structure in older children and adolescents is much greater^47,74^ than it is for neonates.^46,56,57,58,59,61,63,64,65^ By inference, SES associations, which were relatively modest in neonates in comparison with low GA, might accumulate through childhood. Of note, functional outcome data from preterm infants suggest the importance of SES increases after the age of 5 years while the importance of birth events as determinants diminishes.^75^ To address this question, longitudinal imaging and neurodevelopmental assessment of participants is planned.^26^

## Conclusions

In comparison with socioeconomic factors, low birth GA was associated with more widely distributed measures of brain structure in preterm infants at term-equivalent age. However, family-level SES measures of parental education and occupation were associated with neonatal brain development, and they interacted with low GA. This suggests that strategies designed to mitigate the adverse effects of family-level disadvantage during neonatal intensive care could improve the brain development of preterm infants. Further research is warranted to understand the biological mechanisms that underlie associations of preterm birth and level-specific social disadvantage with brain development.

## Notes

Supplement 1. **eMethods.** Case Definitions and Sources **eFigure 1.** Flow Diagram of Participants **eFigure 2.** Diagram of Variables and Covariates in Models **eTable 1.** Comparison of Included and Excluded Participants **eTable 2.** Clinical Features of the Preterm Sample **eTable 3.** Regional Brain Volumes: Association With Gestational Age, the Scottish Index of Multiple Deprivation, and Interaction Effect (Fully Adjusted Ridge Regression Model) **eTable 4.** Regional Brain Volumes: Association With Gestational Age, the Scottish Index of Multiple Deprivation, and Interaction Effect (Baseline Unadjusted Linear Regression Model) **eTable 5.** Cortical Measures: Association With Gestational Age and the Scottish Index of Multiple Deprivation (Baseline Unadjusted Linear Regression Model) **eTable 6.** Regional Brain Volumes: Association With Gestational Age, Maternal Final Educational Qualification, and Interaction Effect (Fully Adjusted Ridge Regression Model) **eTable 7. **Regional Brain Volumes: Association With Gestational Age, Paternal Final Educational Qualification, and Interaction Effect (Fully Adjusted Ridge Regression Model) **eTable 8.** Regional Brain Volumes: Association With Gestational Age, Maternal Occupation, and Interaction Effect (Fully Adjusted Ridge Regression Model) **eTable 9.** Regional Brain Volumes: Association With Gestational Age, Paternal Occupation, and Interaction Effect (Fully Adjusted Ridge Regression Model) **eTable 10.** Regional Brain Volumes: Association With Gestational Age, Subjective Socioeconomic Status, and Interaction Effect (Fully Adjusted Ridge Regression Model) **eTable 11.** Cortical Measures: Association With Gestational Age and Maternal Final Educational Qualification (Fully Adjusted Ridge Regression Model) **eTable 12.** Cortical Measures: Association With Gestational Age and Paternal Final Educational Qualification (Fully Adjusted Ridge Regression Model) **eTable 13.** Cortical Measures: Association With Gestational Age, Maternal Occupation, and Interaction Effect (Fully Adjusted Ridge Regression Model) **eTable 14.** Cortical Measures: Association With Gestational Age and Paternal Occupation (Fully Adjusted Ridge Regression Model) **eTable 15.** Cortical Measures: Association With Gestational Age, Subjective Socioeconomic Status, and Interaction Effect (Fully Adjusted Ridge Regression Model) **eTable 16.** Lobar Volumes: Association With Gestational Age and the Scottish Index of Multiple Deprivation (Fully Adjusted Ridge Regression Models) **eTable 17.** Lobar Volumes: Association With Gestational Age and Maternal Education (Fully Adjusted Ridge Regression Models) **eTable 18.** Lobar Volumes: Association With Gestational Age and Paternal Education (Fully Adjusted Ridge Regression Models) **eTable 19.** Lobar Volumes: Association With Gestational Age and Maternal Occupation (Fully Adjusted Ridge Regression Models) **eTable 20.** Lobar Volumes: Association With Gestational Age and Paternal Occupation (Fully Adjusted Ridge Regression Models) **eTable 21.** Lobar Volumes: Association With Gestational Age and Subjective Socioeconomic Status (Fully Adjusted Ridge Regression Models) **eTable 22.** Cortical Measures: Association With Gestational Age, the Scottish Index of Multiple Deprivation, and Interaction Effect (Fully Adjusted Ridge Regression Models) **eTable 23.** Cortical Measures: Association With Gestational Age, Maternal Education, and Interaction Effect (Fully Adjusted Ridge Regression Models) **eTable 24.** Cortical Measures: Association With Gestational Age, Paternal Education, and Interaction Effect (Fully Adjusted Ridge Regression Models) **eTable 25.** Cortical Measures: Association With Gestational Age, Maternal Occupation, and Interaction Effect (Fully Adjusted Ridge Regression Models) **eTable 26.** Cortical Measures: Association With Gestational Age, Paternal Occupation, and Interaction Effect (Fully Adjusted Ridge Regression Models) **eTable 27.** Cortical Measures: Association With Gestational Age, Subjective Socioeconomic Status, and Interaction Effect (Fully Adjusted Ridge Regression Models)
eReferences.

Supplement 2.
Data Sharing Statement
