# Supplementary material for: Association of Preterm Birth and Socioeconomic Status With Neonatal Brain Structure
Source: JAMA Netw Open. 2023 May 31;6(5):e2316067. doi: 10.1001/jamanetworkopen.2023.16067 (PMC10233421; doi:10.1001/jamanetworkopen.2023.16067)
Supplement: Supplement 1. — eMethods. Case Definitions and Sources eFigure 1. Flow Diagram of Participants eFigure 2. Diagram of Variables and Covariates in Models eTable 1. Comparison of Included and Excluded Participants eTable 2. Clinical Features of the Preterm Sample eTable 3. Regional Brain Volumes: Association With Gestational Age, the Scottish Index of Multiple Deprivation, and Interaction Effect (Fully Adjusted Ridge Regression Model) eTable 4. Regional Brain Volumes: Association With Gestational Age, the Scottish Index of Multiple Deprivation, and Interaction Effect (Baseline Unadjusted Linear Regression Model) eTable 5. Cortical Measures: Association With Gestational Age and the Scottish Index of Multiple Deprivation (Baseline Unadjusted Linear Regression Model) eTable 6. Regional Brain Volumes: Association With Gestational Age, Maternal Final Educational Qualification, and Interaction Effect (Fully Adjusted Ridge Regression Model) eTable 7. Regional Brain Volumes: Association With Gestational Age, Paternal Final Educational Qualification, and Interaction Effect (Fully Adjusted Ridge Regression Model) eTable 8. Regional Brain Volumes: Association With Gestational Age, Maternal Occupation, and Interaction Effect (Fully Adjusted Ridge Regression Model) eTable 9. Regional Brain Volumes: Association With Gestational Age, Paternal Occupation, and Interaction Effect (Fully Adjusted Ridge Regression Model) eTable 10. Regional Brain Volumes: Association With Gestational Age, Subjective Socioeconomic Status, and Interaction Effect (Fully Adjusted Ridge Regression Model) eTable 11. Cortical Measures: Association With Gestational Age and Maternal Final Educational Qualification (Fully Adjusted Ridge Regression Model) eTable 12. Cortical Measures: Association With Gestational Age and Paternal Final Educational Qualification (Fully Adjusted Ridge Regression Model) eTable 13. Cortical Measures: Association With Gestational Age, Maternal Occupation, and Interaction Effect (Fully Adjusted Ridge Reg [file jamanetwopen-e2316067-s001.pdf]

Supplemental Online Content

Mckinnon K, Galdi P, Blesa-Cábez M, et al. Association of preterm birth and socioeconomic status with neonatal brain structure. *JAMA Netw Open.* 2023;6(5):e2316067. doi:10.1001/jamanetworkopen.2023.16067

eMethods. Case Definitions and Sources

eFigure 1. Flow Diagram of Participants

eFigure 2. Diagram of Variables and Covariates in Models

eTable 1. Comparison of Included and Excluded Participants

eTable 2. Clinical Features of the Preterm Sample

eTable 3. Regional Brain Volumes: Association With Gestational Age, the Scottish Index of Multiple Deprivation, and Interaction Effect (Fully Adjusted Ridge Regression Model)

eTable 4. Regional Brain Volumes: Association With Gestational Age, the Scottish Index of Multiple Deprivation, and Interaction Effect (Baseline Unadjusted Linear Regression Model)

eTable 5. Cortical Measures: Association With Gestational Age and the Scottish Index of Multiple Deprivation (Baseline Unadjusted Linear Regression Model)

eTable 6. Regional Brain Volumes: Association With Gestational Age, Maternal Final Educational Qualification, and Interaction Effect (Fully Adjusted Ridge Regression Model)

eTable 7. Regional Brain Volumes: Association With Gestational Age, Paternal Final Educational Qualification, and Interaction Effect (Fully Adjusted Ridge Regression Model)

eTable 8. Regional Brain Volumes: Association With Gestational Age, Maternal Occupation, and Interaction Effect (Fully Adjusted Ridge Regression Model)

eTable 9. Regional Brain Volumes: Association With Gestational Age, Paternal Occupation, and Interaction Effect (Fully Adjusted Ridge Regression Model)

eTable 10. Regional Brain Volumes: Association With Gestational Age, Subjective Socioeconomic Status, and Interaction Effect (Fully Adjusted Ridge Regression Model)

eTable 11. Cortical Measures: Association With Gestational Age and Maternal Final Educational Qualification (Fully Adjusted Ridge Regression Model)

eTable 12. Cortical Measures: Association With Gestational Age and Paternal

© 2023 Mckinnon K et al. *JAMA Network Open.*

Final Educational Qualification (Fully Adjusted Ridge Regression Model)

eTable 13. Cortical Measures: Association With Gestational Age, Maternal Occupation, and Interaction Effect (Fully Adjusted Ridge Regression Model)

eTable 14. Cortical Measures: Association With Gestational Age and Paternal Occupation (Fully Adjusted Ridge Regression Model)

eTable 15. Cortical Measures: Association With Gestational Age, Subjective Socioeconomic Status, and Interaction Effect (Fully Adjusted Ridge Regression Model)

eTable 16. Lobar Volumes: Association With Gestational Age and the Scottish Index of Multiple Deprivation (Fully Adjusted Ridge Regression Models)

eTable 17. Lobar Volumes: Association With Gestational Age and Maternal Education (Fully Adjusted Ridge Regression Models)

eTable 18. Lobar Volumes: Association With Gestational Age and Paternal Education (Fully Adjusted Ridge Regression Models)

eTable 19. Lobar Volumes: Association With Gestational Age and Maternal Occupation (Fully Adjusted Ridge Regression Models)

eTable 20. Lobar Volumes: Association With Gestational Age and Paternal Occupation (Fully Adjusted Ridge Regression Models)

eTable 21. Lobar Volumes: Association With Gestational Age and Subjective Socioeconomic Status (Fully Adjusted Ridge Regression Models)

eTable 22. Cortical Measures: Association With Gestational Age, the Scottish Index of Multiple Deprivation, and Interaction Effect (Fully Adjusted Ridge Regression Models)

eTable 23. Cortical Measures: Association With Gestational Age, Maternal Education, and Interaction Effect (Fully Adjusted Ridge Regression Models)

eTable 24. Cortical Measures: Association With Gestational Age, Paternal Education, and Interaction Effect (Fully Adjusted Ridge Regression Models)

eTable 25. Cortical Measures: Association With Gestational Age, Maternal Occupation, and Interaction Effect (Fully Adjusted Ridge Regression Models)

eTable 26. Cortical Measures: Association With Gestational Age, Paternal Occupation, and Interaction Effect (Fully Adjusted Ridge Regression Models)

eTable 27. Cortical Measures: Association With Gestational Age, Subjective Socioeconomic Status, and Interaction Effect (Fully Adjusted Ridge Regression Models)

eReferences.

This supplemental material has been provided by the authors to give readers additional information about their work.

© 2023 Mckinnon K et al. *JAMA Network Open.*

eMethods. Case Definitions and Sources

Gestational age at birth: from medical records, based on dating ultrasound scan at 12 weeks' gestation if available, or first scan beyond this point.
Birthweight: from medical records, measured at birth.
Head circumference: from medical records, measured at birth.
Maternal smoking during pregnancy: yes/no, at any point during pregnancy, parental questionnaire after birth.
Multiple pregnancy: twins or higher multiples, from medical records.
Breast milk at discharge: breast fed or mixed fed at discharge from hospital, parental questionnaire.

Child ethnicity: parental questionnaire after birth.
- Asian: Indian, Pakistani, Bangladeshi, Chinese, any other Asian background.
- Black: Caribbean, African, any other African background.
- Mixed ethnicity: White and Black Caribbean, White and Black African, White and Asian, any other mixed background.
- White: any White background.
- Other ethnic group: any other ethnic group, with blank space to complete. This included Arab, Iraqi, Bulgarian/Turkish, Fijian.

Scottish Index of Multiple Deprivation: derived from postcode at birth. This is developed using information in the following domains[1]; geography (data zone, council area); population (total population, working age population), income (rate and count), employment (rate and count); health (comparative illness factor, hospital stays related to drug/alcohol use, standardized mortality ratio, population prescribed drugs for anxiety/depression/psychosis, rates of low birth weight, emergency stays in hospital); education, skills and training (school attendance, attainment of school leavers, working age people without qualifications, people 16-19 not in education/employment/training, people 17-21 entering university); geographic access to services (drive time to petrol station/GP/post office/primary school/retail center/secondary school, public transport travel time to GP/post office/retail center, percentage without access to superfast broadband); crime (recorded crimes of violence, sexual offences, domestic housebreaking, vandalism, drug offences, common assault); housing (rates of overcrowded households, households without central heating).

Parent age leaving education: parental questionnaire after birth.

Parent final educational qualification: parental questionnaire after birth.
- None: no qualifications obtained.
- Basic high school qualification: National 5s, Standard Grades, GCSEs (General Certificates of Secondary Education) or equivalent. For the purposes of analysis, this was divided into 1-4 and >5 qualifications.
- Advanced high school qualification: Highers, A levels (Advanced levels) or equivalent.
- College qualification: e.g. National Certificate, Higher National Diploma, Higher National Certificate, vocational qualifications.
- University undergraduate degree.
- University postgraduate degree.
- Not applicable.

Parent current/recent job: parental questionnaire after birth.
- Professional: e.g. doctors, lawyers, teachers, managers.
- Non-manual skilled: e.g. typist, police officer, fireman.
- Manual skilled: e.g. toolmaker, foreman, ambulance man.
- Partly skilled: e.g. bus conductor, postman.
- Unskilled: cleaners, porters, messengers.
- Unemployed: for the majority of adult life.
- Homemaker.
- Still in full time education.

World Health Organization Quality of Life – environment domain: parental questionnaire at term-corrected age (at time of MRI). This includes questions on the following domains[2]; financial resources; freedom, physical safety and security; health and social care – accessibility and quality; home environment; opportunities for acquiring new information and skills; participation in and opportunities for recreation/leisure activities; physical environment (pollution, noise, traffic, climate); transport.

© 2023 Mckinnon K et al. *JAMA Network Open.*

Preterm sample clinical features: from medical records.

- Sepsis: positive blood culture and/or physician decision to treat with five days of antibiotics; early-onset is <72 hours after birth and late-onset is after 72 hours.
- Retinopathy of prematurity: requiring treatment.
- Bronchopulmonary dysplasia: requirement for respiratory support and/or supplemental oxygen after 36 weeks' gestation.
- Necrotising enterocolitis: medical (7 days nil by mouth) or surgical management.
- Antenatal steroids: at least one dose of maternal steroids given for fetal lung maturation.
- Antenatal magnesium sulphate: magnesium sulphate given to mother in the antenatal period.
- Postnatal steroids: dexamethasone given to ventilator-dependent infants to facilitate extubation.

© 2023 Mckinnon K et al. *JAMA Network Open.*

eFigure 1. Flow Diagram of Participants

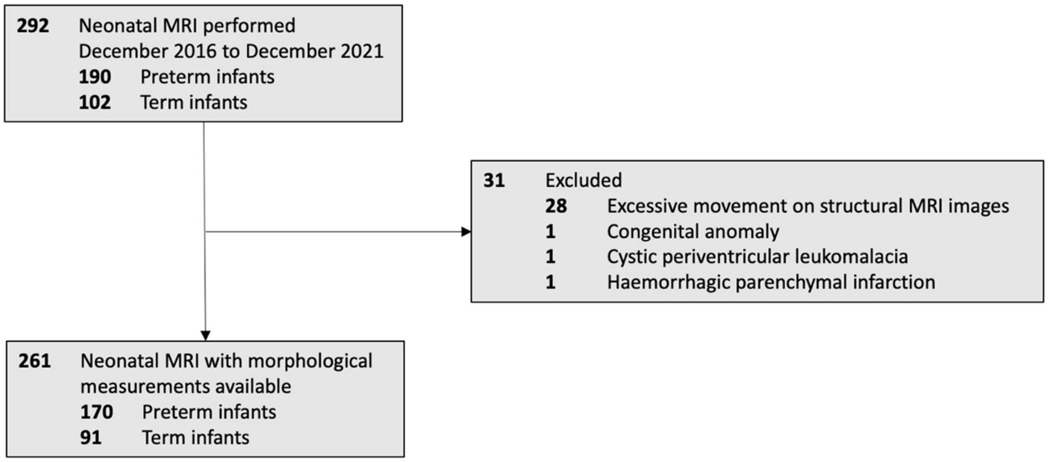

Study exclusion criteria[3] were major congenital malformation, chromosomal abnormality, congenital infection, cystic periventricular leukomalacia, hemorrhagic parenchymal infarction, and post-hemorrhagic ventricular dilatation. In individuals excluded following MRI, these lesions had not been identified on prior routine ultrasound in the neonatal unit.

© 2023 Mckinnon K et al. *JAMA Network Open.*

eFigure 2. Diagram of Variables and Covariates in Models

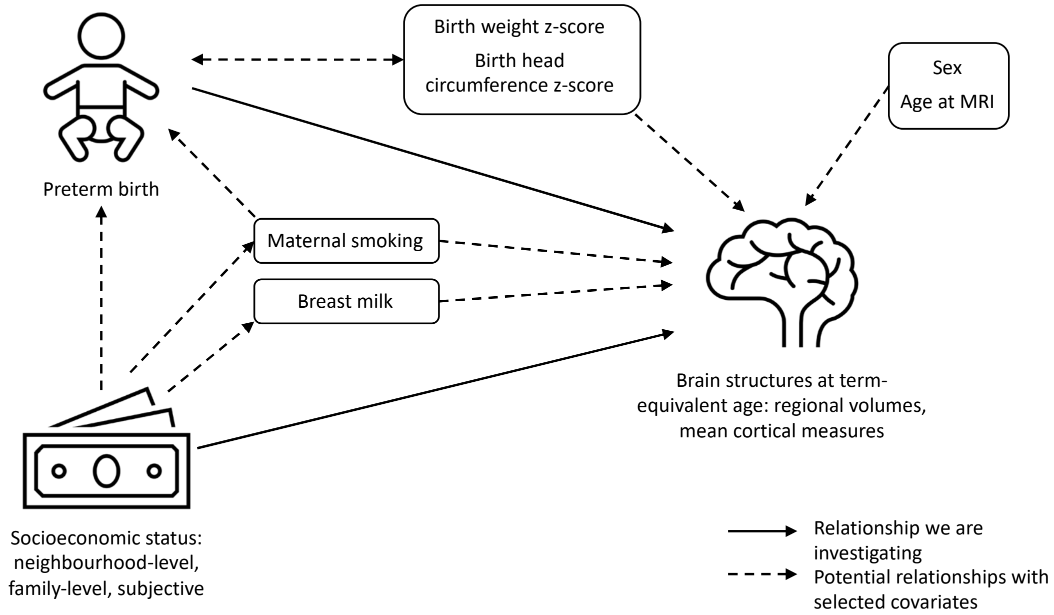

Diagram demonstrating the potential interrelationships between preterm birth, socioeconomic status and brain structure, with covariates. This study aims to investigate the relationship between preterm birth, SES (at neighborhood-, family-, and individual-levels), and brain structures – shown with a solid arrow. The dashed arrows show the potential relationships with selected covariates.

© 2023 Mckinnon K et al. *JAMA Network Open.*

eTable 1. Comparison of Included and Excluded Participants

**Measure**	**Included in study (n=261)**	**Excluded from study (n=31)**	***p* value**
Gestational age at birth (weeks) - median (range)	31+2 (22+1 - 42+1)	30+0 (24+0 - 41+4)	.56
Gestational age at MRI (weeks) - median (range)	41+1 (36+2 - 46+1)	41+5 (37+7 - 45+1)	.41
Birthweight (grams) - median (range)	1620 (370 - 4560)	1445 (454 - 4170)	.89
Birthweight z-score - median (range)	0.25 (-3.13 - 2.57)	0.25 (-5.07 - 2.18)	.40
Head circumference (cm) - median (range)[a]	28.9 (17.5 - 39)	27.5 (22.1 - 37)	.22
Head circumference z-score - median (range)[a]	0.14 (-3.13 - 2.57)	0.077 (-29 - 2.07)	.45
Sex (M:F)	145:116	15:16	.90
Maternal smoking during pregnancy	33/258 (12.8%)	4/31 (12.9%)	.99
Multiple pregnancy	53/261 (20.3%)	3/31 (9.7%)	.15
Any breast milk at discharge	207/258 (80.2%)	28/31 (90.3%)	.90
Child ethnicity			
Asian	7/252 (2.8%)	3/30 (10.0%)	.08
Black	2/252 (0.8%)	1/30 (3.3%)	
Mixed ethnicity	14/252 (5.6%)	4/30 (13.3%)	
White	224/252 (88.9%)	21/30 (70.0%)	
Other ethnic group[b]	5/252 (2.0%)	1/30 (3.3%)	
SIMD rank - median (range)	4510 (6-6967)	3720 (51 - 6966)	.56
Mother age leaving education (years) - median (range)[c]	21 (14 - 36)	22 (16 - 32)	.15
Father age leaving education (years) - median (range)[d]	21 (14 - 36)	21 (16 - 36)	.14
Mother highest educational qualification			
None	5/256 (2.0%)	0/31 (0.0%)	.74
Basic high school qualification	23/256 (9.0%)	3/31 (9.7%)	
Advanced high school qualification	15/256 (5.9%)	1/31 (3.2%)	
College qualification	51/256 (19.9%)	8/31 (25.8%)	
University undergraduate degree	84/256 (32.8%)	10/31 (32.3%)	
University postgraduate degree	69/256 (27.0%)	9/31 (29.0%)	
Not applicable	9/256 (3.5%)	1/31 (3.2%)	
Father highest educational qualification			
None	4/238 (1.7%)	0/30 (0.0%)	.50
Basic high school qualification	40/238 (16.8%)	4/30 (13.3%)	
Advanced high school qualification	18/238 (7.6%)	3/30 (10.0%)	
College qualification	45/238 (18.9%)	5/30 (16.7%)	
University undergraduate degree	74/238 (31.1%)	8/30 (26.7%)	
University postgraduate degree	47/238 (19.7%)	10/30 (33.3%)	
Not applicable	10/238 (4.2%)	0/30 (0.0%)	

© 2023 Mckinnon K et al. *JAMA Network Open.*

Measure	Included in study (n=261)	Excluded from study (n=31)	*p* value
Mother current/recent job			
Professional	151/257 (58.8%)	17/31 (54.8%)	.85
Non-manual skilled	38/257 (14.8%)	9/31 (29.0%)	
Manual skilled	17/257 (6.6%)	0/31 (0.0%)	
Partly skilled	12/257 (4.7%)	1/31 (3.2%)	
Unskilled	16/257 (6.2%)	1/31 (3.2%)	
Unemployed	4/257 (1.6%)	1/31 (3.2%)	
Homemaker	10/257 (3.9%)	1/31 (3.2%)	
Still in full time education	8/257 (3.1%)	0/31 (0.0%)	
Partner current/ recent job			
Professional	122/248 (49.2%)	19/30 (63.3%)	.33
Non-manual skilled	29/248 (11.7%)	1/30 (3.3%)	
Manual skilled	44/248 (17.7%)	6/30 (20%)	
Partly skilled	28/248 (11.3%)	3/30 (10.0%)	
Unskilled	9/248 (3.6%)	1/30 (3.3%)	
Unemployed	3/248 (1.2%)	0/30 (0.0%)	
Homemaker	3/248 (1.2%)	0/30 (0.0%)	
Still in full time education	9/248 (3.6%)	0/30 (0.0%)	
WHO QoL environment score - median (range)[e]	78.1 (21.9 - 100.0)	81.3 (53.1 - 100)	.48

P values compare individuals included in this study with those excluded following MRI.
For binary data, we used Chi-square testing for *p* values. For continuous data, we used Mann-Whitney U testing for *p* values.
Case definitions are available in eMethods.
[a]For head circumference, and head circumference z-score, included n=240 and excluded n=30.
[b]This included Arab, Iraqi, Bulgarian/Turkish, Fijian.
[c]For maternal age leaving education, included n=241 and excluded n=28.
[d]For partner age leaving education, included n=216 and excluded n=25.
[e]For WHO QoL environment score, included n=242 and excluded n=28.

© 2023 Mckinnon K et al. *JAMA Network Open.*

eTable 2. Clinical Features of the Preterm Sample

Characteristic	Preterm (total n=170)
Early-onset sepsis	14/169 (8.3%)
Late-onset sepsis	27/167 (16.2%)
Retinopathy of prematurity	7/160 (4.4%)
Bronchopulmonary dysplasia	41/168 (24.4%)
Necrotizing enterocolitis	7/166 (4.2%)
Antenatal steroids	162/170 (95.3%)
Antenatal magnesium sulfate	134/170 (78.8%)
Postnatal steroids	6/168 (3.6%)

Case definitions are available in eMethods. Information not available for all children, for example if transferred to another unit prior to discharge home.

© 2023 Mckinnon K et al. *JAMA Network Open.*

eTable 3. Regional Brain Volumes: Association With Gestational Age, the Scottish Index of Multiple Deprivation, and Interaction Effect (Fully Adjusted Ridge Regression Model)

Regional volume	Standardized β coefficient (95% confidence intervals)	Raw *p* value	BH corrected *p* value
Gestation			
Amygdala left	0.08 (-0.02-0.17)	.06	.20
Amygdala right	0.02 (-0.07-0.12)	.57	.70
Anterior temporal lobe lateral part left gray matter	0.14 (0.06-0.23)	<.001	.003
Anterior temporal lobe lateral part left white matter	0.11 (0.01-0.21)	.01	.08
Anterior temporal lobe lateral part right gray matter	0.07 (-0.01-0.15)	.08	.22
Anterior temporal lobe lateral part right white matter	0.09 (-0.004-0.19)	.02	.11
Anterior temporal lobe medial part left gray matter	0.05 (-0.04-0.14)	.26	.47
Anterior temporal lobe medial part left white matter	0.03 (-0.07-0.14)	.37	.53
Anterior temporal lobe medial part right gray matter	0.05 (-0.04-0.15)	.20	.42
Anterior temporal lobe medial part right white matter	0.05 (-0.05-0.15)	.24	.47
Brainstem	-0.03 (-0.11-0.06)	.52	.66
Caudate nucleus left	0.16 (0.07-0.25)	<.001	<.001
Caudate nucleus right	0.13 (0.04-0.22)	<.001	.01
Cerebellum left	-0.01 (-0.08-0.06)	.80	.87
Cerebellum right	-0.03 (-0.10-0.04)	.38	.54
Cerebrospinal fluid	-0.11 (-0.21- -0.005)	<.001	<.001
Cingulate gyrus anterior part left gray matter	0.01 (-0.09-0.10)	.87	.92
Cingulate gyrus anterior part left white matter	0.06 (-0.04-0.16)	.09	.25
Cingulate gyrus anterior part right gray matter	-0.07 (-0.16-0.03)	.10	.26
Cingulate gyrus anterior part right white matter	0.04 (-0.06-0.14)	.27	.48
Cingulate gyrus posterior part left gray matter	-0.08 (-0.17-0.01)	.051	.17
Cingulate gyrus posterior part left white matter	0.05 (-0.04-0.14)	.20	.42
Cingulate gyrus posterior part right gray matter	-0.12 (-0.21- -0.02)	.01	.047
Cingulate gyrus posterior part right white matter	0.07 (-0.01-0.16)	.06	.20
Corpus callosum	0.08 (-0.01-0.17)	.046	.16
Frontal lobe left gray matter	0.02 (-0.06-0.09)	.63	.74
Frontal lobe left white matter	0.20 (0.12-0.28)	<.001	<.001
Frontal lobe right gray matter	0.02 (-0.05-0.10)	.52	.66
Frontal lobe right white matter	0.16 (0.08-0.25)	<.001	<.001
Gyri parahippocampalis et ambiens anterior part left gray matter	0.07 (-0.03-0.17)	.08	.24
Gyri parahippocampalis et ambiens anterior part left white matter	0.16 (0.06-0.26)	<.001	.001
Gyri parahippocampalis et ambiens anterior part right gray matter	0.07 (-0.03-0.17)	.09	.25
Gyri parahippocampalis et ambiens anterior part right white matter	0.09 (-0.01-0.19)	.03	.13
Gyri parahippocampalis et ambiens posterior part left gray matter	0.11 (0.02-0.20)	.01	.06
Gyri parahippocampalis et ambiens posterior part left white matter	0.07 (-0.04-0.17)	.07	.20

© 2023 Mckinnon K et al. *JAMA Network Open.*

**Regional volume**	**Standardized β coefficient (95% confidence intervals)**	**Raw *p* value**	**BH corrected *p* value**
Gyri parahippocampalis et ambiens posterior part right gray matter	0.02 (-0.07-0.12)	.59	.71
Gyri parahippocampalis et ambiens posterior part right white matter	0.14 (0.04-0.24)	<.001	.01
Hippocampus left	-0.08 (-0.17-0.02)	.06	.20
Hippocampus right	-0.06 (-0.16-0.04)	.16	.34
Insula left gray matter	0.04 (-0.04-0.12)	.28	.48
Insula left white matter	0.17 (0.08-0.26)	<.001	<.001
Insula right gray matter	-0.02 (-0.10-0.05)	.53	.67
Insula right white matter	0.16 (0.08-0.25)	<.001	<.001
Lateral occipitotemporal gyrus gyrus fusiformis anterior part left gray matter	0.20 (0.12-0.29)	<.001	<.001
Lateral occipitotemporal gyrus gyrus fusiformis anterior part left white matter	-0.08 (-0.18-0.02)	.06	.20
Lateral occipitotemporal gyrus gyrus fusiformis anterior part right gray matter	0.15 (0.07-0.24)	<.001	.001
Lateral occipitotemporal gyrus gyrus fusiformis anterior part right white matter	-0.04 (-0.14-0.06)	.38	.54
Lateral occipitotemporal gyrus gyrus fusiformis posterior part left gray matter	0.11 (0.02-0.20)	.01	.06
Lateral occipitotemporal gyrus gyrus fusiformis posterior part left white matter	-0.04 (-0.14-0.05)	.29	.49
Lateral occipitotemporal gyrus gyrus fusiformis posterior part right gray matter	0.09 (-0.01-0.18)	.03	.13
Lateral occipitotemporal gyrus gyrus fusiformis posterior part right white matter	0.005 (-0.10-0.11)	.89	.92
Lateral ventricle left	-0.12 (-0.23- -0.02)	<.001	.001
Lateral ventricle right	-0.10 (-0.20-0.004)	<.001	.002
Lentiform nucleus left	-0.07 (-0.15-0.02)	.10	.25
Lentiform nucleus right	-0.10 (-0.18- -0.02)	.02	.09
Medial and inferior temporal gyri anterior part left gray matter	0.22 (0.15-0.29)	<.001	<.001
Medial and inferior temporal gyri anterior part left white matter	0.04 (-0.05-0.13)	.31	.50
Medial and inferior temporal gyri anterior part right gray matter	0.22 (0.16-0.29)	<.001	<.001
Medial and inferior temporal gyri anterior part right white matter	0.01 (-0.09-0.11)	.78	.86
Medial and inferior temporal gyri posterior part left gray matter	0.11 (0.05-0.18)	<.001	.005
Medial and inferior temporal gyri posterior part left white matter	-0.06 (-0.16-0.04)	.17	.36
Medial and inferior temporal gyri posterior part right gray matter	0.14 (0.07-0.20)	<.001	<.001
Medial and inferior temporal gyri posterior part right white matter	-0.04 (-0.14-0.06)	.30	.50
Occipital lobe left gray matter	-0.02 (-0.09-0.04)	.48	.64
Occipital lobe left white matter	-0.08 (-0.18-0.03)	.09	.24
Occipital lobe right gray matter	0.01 (-0.07-0.08)	.85	.92
Occipital lobe right white matter	-0.06 (-0.16-0.04)	.16	.34
Parietal lobe left gray matter	0.07 (0.01-0.12)	.02	.09
Parietal lobe left white matter	0.11 (0.02-0.20)	.01	.06
Parietal lobe right gray matter	0.09 (0.03-0.15)	.001	.01

© 2023 Mckinnon K et al. *JAMA Network Open.*

Regional volume	Standardized β coefficient (95% confidence intervals)	Raw *p* value	BH corrected *p* value
Parietal lobe right white matter	0.07 (-0.03-0.16)	.11	.26
Subthalamic nucleus left	-0.13 (-0.23- -0.03)	.003	.03
Subthalamic nucleus right	-0.10 (-0.19-0.002)	.01	.06
Superior temporal gyrus middle part left gray matter	0.08 (-0.0002-0.16)	.04	.14
Superior temporal gyrus middle part left white matter	0.06 (-0.04-0.16)	.13	.29
Superior temporal gyrus middle part right gray matter	0.11 (0.04-0.18)	.001	.01
Superior temporal gyrus middle part right white matter	0.05 (-0.05-0.14)	.27	.47
Superior temporal gyrus posterior part left gray matter	0.06 (-0.02-0.14)	.10	.25
Superior temporal gyrus posterior part left white matter	0.03 (-0.07-0.13)	.36	.52
Superior temporal gyrus posterior part right gray matter	0.04 (-0.04-0.12)	.26	.47
Superior temporal gyrus posterior part right white matter	0.04 (-0.06-0.13)	.35	.52
Thalamus left high intensity part in T2	0.005 (-0.07-0.08)	.91	.92
Thalamus left low intensity part in T2	0.01 (-0.10-0.12)	.27	.47
Thalamus right high intensity part in T2	-0.03 (-0.12-0.05)	.39	.55
Thalamus right low intensity part in T2	0.02 (-0.08-0.12)	.67	.79
Interaction			
Cingulate gyrus anterior part left white matter	0.03 (-0.06-0.12)	.68	.79
Gyri parahippocampalis et ambiens anterior part left white matter	0.04 (-0.05-0.12)	.03	.12
Lateral occipitotemporal gyrus gyrus fusiformis anterior part right white matter	0.04 (-0.05-0.12)	.04	.16
Lentiform nucleus right	-0.06 (-0.13-0.01)	.03	.12
Medial and inferior temporal gyri anterior part right white matter	-0.04 (-0.12-0.05)	.02	.10
Occipital lobe left gray matter	-0.06 (-0.12- -0.001)	.01	.08
SIMD			
Amygdala left	0.05 (-0.07-0.18)	.36	.52
Amygdala right	-0.06 (-0.18-0.07)	.33	.51
Anterior temporal lobe lateral part left gray matter	-0.004 (-0.11-0.10)	.93	.94
Anterior temporal lobe lateral part left white matter	0.04 (-0.09-0.17)	.46	.62
Anterior temporal lobe lateral part right gray matter	0.05 (-0.06-0.15)	.38	.54
Anterior temporal lobe lateral part right white matter	-0.01 (-0.14-0.12)	.89	.92
Anterior temporal lobe medial part left gray matter	0.02 (-0.10-0.14)	.77	.85
Anterior temporal lobe medial part left white matter	0.02 (-0.12-0.16)	.63	.75
Anterior temporal lobe medial part right gray matter	0.01 (-0.11-0.13)	.91	.92
Anterior temporal lobe medial part right white matter	0.07 (-0.06-0.20)	.22	.44
Brainstem	0.12 (0.003-0.23)	.03	.13
Caudate nucleus left	0.08 (-0.03-0.20)	.10	.26
Caudate nucleus right	0.10 (-0.02-0.22)	.06	.20
Cerebellum left	0.04 (-0.05-0.14)	.34	.52
Cerebellum right	0.06 (-0.03-0.15)	.19	.41

© 2023 Mckinnon K et al. *JAMA Network Open.*

Regional volume	Standardized β coefficient (95% confidence intervals)	Raw *p* value	BH corrected *p* value
Cerebrospinal fluid	0.01 (-0.12-0.15)	.52	.66
Cingulate gyrus anterior part left gray matter	0.05 (-0.07-0.18)	.31	.50
Cingulate gyrus anterior part left white matter	0.02 (-0.32-0.35)	.02	.09
Cingulate gyrus anterior part right gray matter	-0.03 (-0.16-0.10)	.59	.71
Cingulate gyrus anterior part right white matter	0.05 (-0.08-0.18)	.31	.50
Cingulate gyrus posterior part left gray matter	0.01 (-0.10-0.12)	.89	.92
Cingulate gyrus posterior part left white matter	0.11 (-0.005-0.22)	.04	.14
Cingulate gyrus posterior part right gray matter	0.02 (-0.10-0.14)	.75	.85
Cingulate gyrus posterior part right white matter	0.10 (-0.02-0.21)	.07	.20
Corpus callosum	0.12 (-0.001-0.24)	.03	.12
Frontal lobe left gray matter	0.07 (-0.02-0.17)	.12	.29
Frontal lobe left white matter	0.06 (-0.05-0.16)	.26	.47
Frontal lobe right gray matter	0.03 (-0.07-0.13)	.55	.69
Frontal lobe right white matter	0.12 (0.01-0.23)	.02	.10
Gyri parahippocampalis et ambiens anterior part left gray matter	0.03 (-0.11-0.16)	.58	.71
Gyri parahippocampalis et ambiens anterior part left white matter	-0.10 (-0.43-0.23)	.11	.26
Gyri parahippocampalis et ambiens anterior part right gray matter	-0.02 (-0.15-0.11)	.74	.84
Gyri parahippocampalis et ambiens anterior part right white matter	0.03 (-0.09-0.16)	.55	.69
Gyri parahippocampalis et ambiens posterior part left gray matter	0.13 (0.01-0.26)	.02	.10
Gyri parahippocampalis et ambiens posterior part left white matter	0.06 (-0.08-0.20)	.23	.45
Gyri parahippocampalis et ambiens posterior part right gray matter	0.06 (-0.07-0.19)	.32	.51
Gyri parahippocampalis et ambiens posterior part right white matter	-0.02 (-0.15-0.11)	.71	.81
Hippocampus left	0.06 (-0.07-0.02)	.31	.50
Hippocampus right	0.03 (-0.10-0.16)	.59	.71
Insula left gray matter	-0.04 (-0.14-0.07)	.48	.64
Insula left white matter	0.12 (0.01-0.24)	.02	.10
Insula right gray matter	-0.001 (-0.10-0.10)	.98	.98
Insula right white matter	0.09 (-0.02-0.21)	.07	.21
Lateral occipitotemporal gyrus gyrus fusiformis anterior part left gray matter	0.13 (0.01-0.25)	.02	.09
Lateral occipitotemporal gyrus gyrus fusiformis anterior part left white matter	0.09 (-0.03-0.22)	.10	.25
Lateral occipitotemporal gyrus gyrus fusiformis anterior part right gray matter	0.07 (-0.04-0.18)	.17	.36
Lateral occipitotemporal gyrus gyrus fusiformis anterior part right white matter	0.01 (-0.32-0.34)	.87	.92
Lateral occipitotemporal gyrus gyrus fusiformis posterior part left gray matter	0.11 (-0.01-0.23)	.04	.15
Lateral occipitotemporal gyrus gyrus fusiformis posterior part left white matter	0.06 (-0.06-0.19)	.26	.47
Lateral occipitotemporal gyrus gyrus fusiformis posterior part right gray matter	0.05 (-0.07-0.17)	.39	.54

© 2023 Mckinnon K et al. *JAMA Network Open.*

Regional volume	Standardized β coefficient (95% confidence intervals)	Raw *p* value	BH corrected *p* value
Lateral occipitotemporal gyrus gyrus fusiformis posterior part right white matter	0.05 (-0.09-0.18)	.35	.52
Lateral ventricle left	0.08 (-0.06-0.22)	.051	.17
Lateral ventricle right	0.06 (-0.08-0.19)	.11	.26
Lentiform nucleus left	-0.08 (-0.19-0.03)	.13	.29
Lentiform nucleus right	0.10 (-0.17-0.37)	.33	.52
Medial and inferior temporal gyri anterior part left gray matter	0.04 (-0.05-0.14)	.34	.52
Medial and inferior temporal gyri anterior part left white matter	0.07 (-0.05-0.19)	.22	.44
Medial and inferior temporal gyri anterior part right gray matter	0.03 (-0.05-0.11)	.49	.65
Medial and inferior temporal gyri anterior part right white matter	0.17 (-0.16-0.50)	.004	.03
Medial and inferior temporal gyri posterior part left gray matter	0.02 (-0.07-0.10)	.73	.82
Medial and inferior temporal gyri posterior part left white matter	-0.01 (-0.14-0.12)	.88	.92
Medial and inferior temporal gyri posterior part right gray matter	0.005 (-0.08-0.09)	.91	.92
Medial and inferior temporal gyri posterior part right white matter	0.08 (-0.05-0.21)	.13	.29
Occipital lobe left gray matter	0.20 (-0.02-0.42)	.03	.12
Occipital lobe left white matter	0.05 (-0.08-0.19)	.36	.53
Occipital lobe right gray matter	0.02 (-0.07-0.11)	.65	.76
Occipital lobe right white matter	0.07 (-0.06-0.20)	.23	.45
Parietal lobe left gray matter	0.01 (-0.06-0.09)	.69	.80
Parietal lobe left white matter	0.06 (-0.06-0.18)	.26	.47
Parietal lobe right gray matter	0.01 (-0.06-0.09)	.76	.85
Parietal lobe right white matter	0.06 (-0.06-0.17)	.29	.49
Subthalamic nucleus left	-0.03 (-0.17-0.10)	.56	.69
Subthalamic nucleus right	0.06 (-0.07-0.18)	.25	.47
Superior temporal gyrus middle part left gray matter	0.01 (-0.10-0.11)	.91	.92
Superior temporal gyrus middle part left white matter	-0.04 (-0.16-0.09)	.50	.65
Superior temporal gyrus middle part right gray matter	-0.01 (-0.10-0.09)	.90	.92
Superior temporal gyrus middle part right white matter	0.07 (-0.06-0.19)	.21	.42
Superior temporal gyrus posterior part left gray matter	0.05 (-0.05-0.16)	.28	.48
Superior temporal gyrus posterior part left white matter	0.04 (-0.09-0.17)	.43	.59
Superior temporal gyrus posterior part right gray matter	0.05 (-0.06-0.15)	.35	.52
Superior temporal gyrus posterior part right white matter	0.01 (-0.11-0.14)	.80	.87
Thalamus left high intensity part in T2	-0.11 (-0.21- -0.001)	.04	.14
Thalamus left low intensity part in T2	0.02 (-0.13-0.16)	.41	.57
Thalamus right high intensity part in T2	-0.09 (-0.20-0.02)	.09	.25
Thalamus right low intensity part in T2	-0.04 (-0.17-0.09)	.47	.63

Fully adjusted ridge regression model, including gestation at birth, SIMD, gestation at MRI, the interaction term (where significant), birth weight z-score, birth head circumference z-score, sex, smoking in pregnancy, and breast milk at discharge. Corrected for false discovery rate.
BH = Benjamini-Hochberg correction, SIMD = Scottish Index of Multiple Deprivation.

© 2023 Mckinnon K et al. *JAMA Network Open.*

eTable 4. Regional Brain Volumes: Association With Gestational Age, the Scottish Index of Multiple Deprivation, and Interaction Effect (Baseline Unadjusted Linear Regression Model)

Regional volume	Standardized β coefficient (95% confidence intervals)	Raw *p* value	BH corrected *p* value
Gestation			
Amygdala left	0.12 (0.03-0.21)	.01	.03
Amygdala right	0.07 (-0.03-0.16)	.16	.28
Anterior temporal lobe lateral part left gray matter	0.19 (0.11-0.26)	<.001	<.001
Anterior temporal lobe lateral part left white matter	0.15 (0.07-0.24)	<.001	.003
Anterior temporal lobe lateral part right gray matter	0.13 (0.05-0.20)	.002	.01
Anterior temporal lobe lateral part right white matter	0.16 (0.07-0.25)	<.001	.002
Anterior temporal lobe medial part left gray matter	0.10 (0.02-0.19)	.02	.051
Anterior temporal lobe medial part left white matter	0.11 (0.01-0.20)	.03	.08
Anterior temporal lobe medial part right gray matter	0.10 (0.02-0.19)	.02	.051
Anterior temporal lobe medial part right white matter	0.12 (0.03-0.22)	.01	.03
Brainstem	0.06 (-0.02-0.15)	.13	.24
Caudate nucleus left	0.26 (0.18-0.34)	<.001	<.001
Caudate nucleus right	0.23 (0.15-0.32)	<.001	<.001
Cerebellum left	0.07 (0.002-0.14)	.045	.10
Cerebellum right	0.04 (-0.02-0.11)	.19	.32
Cerebrospinal fluid	-0.33 (-0.42- -0.24)	<.001	<.001
Cingulate gyrus anterior part left gray matter	0.05 (-0.04-0.15)	.27	.40
Cingulate gyrus anterior part left white matter	0.17 (0.08-0.27)	<.001	.002
Cingulate gyrus anterior part right gray matter	-0.03 (-0.12-0.07)	.60	.71
Cingulate gyrus anterior part right white matter	0.13 (0.04-0.23)	.01	.03
Cingulate gyrus posterior part left gray matter	0.01 (-0.08-0.09)	.88	.91
Cingulate gyrus posterior part left white matter	0.17 (0.09-0.26)	<.001	<.001
Cingulate gyrus posterior part right gray matter	-0.04 (-0.13-0.05)	.35	.47
Cingulate gyrus posterior part right white matter	0.20 (0.11-0.28)	<.001	<.001
Corpus callosum	0.16 (0.07-0.24)	<.001	.002
Frontal lobe left gray matter	0.09 (0.01-0.16)	.03	.07
Frontal lobe left white matter	0.34 (0.25-0.42)	<.001	<.001
Frontal lobe right gray matter	0.09 (0.01-0.17)	.02	.06
Frontal lobe right white matter	0.30 (0.21-0.38)	<.001	<.001
Gyri parahippocampalis et ambiens anterior part left gray matter	0.11 (0.02-0.21)	.02	.049
Gyri parahippocampalis et ambiens anterior part left white matter	0.23 (0.14-0.31)	<.001	<.001
Gyri parahippocampalis et ambiens anterior part right gray matter	0.13 (0.04-0.22)	.01	.02
Gyri parahippocampalis et ambiens anterior part right white matter	0.14 (0.05-0.23)	.002	.01
Gyri parahippocampalis et ambiens posterior part left gray matter	0.13 (0.04-0.21)	.003	.01
Gyri parahippocampalis et ambiens posterior part left white matter	0.12 (0.03-0.22)	.01	.04

© 2023 Mckinnon K et al. *JAMA Network Open.*

Regional volume	Standardized β coefficient (95% confidence intervals)	Raw *p* value	BH corrected *p* value
Gyri parahippocampalis et ambiens posterior part right gray matter	0.06 (-0.03-0.15)	.18	.30
Gyri parahippocampalis et ambiens posterior part right white matter	0.23 (0.14-0.32)	.002	.01
Hippocampus left	-0.02 (-0.11-0.07)	.67	.75
Hippocampus right	-0.002 (-0.10-0.09)	.97	.98
Insula left gray matter	0.13 (0.05-0.21)	.001	.01
Insula left white matter	0.29 (0.20-0.38)	<.001	<.001
Insula right gray matter	0.07 (-0.01-0.15)	.08	.16
Insula right white matter	0.29 (0.20-0.38)	<.001	<.001
Lateral occipitotemporal gyrus gyrus fusiformis anterior part left gray matter	0.25 (0.17-0.34)	<.001	<.001
Lateral occipitotemporal gyrus gyrus fusiformis anterior part left white matter	-0.02 (-0.11-0.07)	.72	.80
Lateral occipitotemporal gyrus gyrus fusiformis anterior part right gray matter	0.22 (0.14-0.30)	<.001	<.001
Lateral occipitotemporal gyrus gyrus fusiformis anterior part right white matter	0.04 (-0.05-0.13)	.37	.49
Lateral occipitotemporal gyrus gyrus fusiformis posterior part left gray matter	0.14 (0.05-0.22)	.001	.01
Lateral occipitotemporal gyrus gyrus fusiformis posterior part left white matter	0.02 (-0.07-0.12)	.62	.72
Lateral occipitotemporal gyrus gyrus fusiformis posterior part right gray matter	0.14 (0.05-0.22)	.001	.01
Lateral occipitotemporal gyrus gyrus fusiformis posterior part right white matter	0.07 (-0.03-0.16)	.17	.30
Lateral ventricle left	-0.22 (-0.31- -0.12)	<.001	<.001
Lateral ventricle right	-0.20 (-0.29- -0.10)	<.001	<.001
Lentiform nucleus left	-0.01 (-0.09-0.07)	.79	.85
Lentiform nucleus right	-0.04 (-0.12-0.03)	.28	.40
Medial and inferior temporal gyri anterior part left gray matter	0.29 (0.22-0.35)	<.001	<.001
Medial and inferior temporal gyri anterior part left white matter	0.16 (0.07-0.25)	<.001	.003
Medial and inferior temporal gyri anterior part right gray matter	0.28 (0.23-0.34)	<.001	<.001
Medial and inferior temporal gyri anterior part right white matter	0.11 (0.01-0.20)	.02	.07
Medial and inferior temporal gyri posterior part left gray matter	0.18 (0.12-0.24)	<.001	<.001
Medial and inferior temporal gyri posterior part left white matter	0.04 (-0.06-0.13)	.46	.59
Medial and inferior temporal gyri posterior part right gray matter	0.20 (0.14-0.26)	<.001	<.001
Medial and inferior temporal gyri posterior part right white matter	0.04 (-0.06-0.13)	.42	.55
Occipital lobe left gray matter	0.05 (-0.01-0.11)	.12	.23
Occipital lobe left white matter	-0.003 (-0.10-0.09)	.95	.96
Occipital lobe right gray matter	0.08 (0.01-0.15)	.02	.06
Occipital lobe right white matter	0.03 (-0.07-0.12)	.60	.71
Parietal lobe left gray matter	0.17 (0.11-0.23)	<.001	<.001
Parietal lobe left white matter	0.24 (0.14-0.33)	<.001	<.001
Parietal lobe right gray matter	0.18 (0.12-0.24)	<.001	<.001

© 2023 Mckinnon K et al. *JAMA Network Open.*

Regional volume	Standardized β coefficient (95% confidence intervals)	Raw *p* value	BH corrected *p* value
Parietal lobe right white matter	0.20 (0.10-0.29)	<.001	<.001
Subthalamic nucleus left	-0.12 (-0.21- -0.02)	.01	.04
Subthalamic nucleus right	-0.09 (-0.18-0.003)	.06	.13
Superior temporal gyrus middle part left gray matter	0.16 (0.08-0.24)	<.001	<.001
Superior temporal gyrus middle part left white matter	0.17 (0.08-0.26)	<.001	.003
Superior temporal gyrus middle part right gray matter	0.18 (0.11-0.25)	<.001	<.001
Superior temporal gyrus middle part right white matter	0.14 (0.04-0.23)	.004	.02
Superior temporal gyrus posterior part left gray matter	0.14 (0.06-0.21)	<.001	.003
Superior temporal gyrus posterior part left white matter	0.13 (0.03-0.22)	.01	.03
Superior temporal gyrus posterior part right gray matter	0.13 (0.05-0.20)	.001	.01
Superior temporal gyrus posterior part right white matter	0.15 (0.05-0.25)	.002	.01
Thalamus left high intensity part in T2	0.09 (0.01-0.16)	.02	.06
Thalamus left low intensity part in T2	0.06 (-0.04-0.15)	.24	.37
Thalamus right high intensity part in T2	0.05 (-0.03-0.13)	.24	.37
Thalamus right low intensity part in T2	0.03 (-0.06-0.11)	.56	.68
Interaction			
Cerebrospinal fluid	-0.11 (-0.20- -0.03)	.01	.03
Corpus callosum	-0.09 (-0.17- -0.01)	.03	.08
Gyri parahippocampalis et ambiens anterior part left white matter	0.09 (0.01-0.17)	.04	.09
Lateral ventricle left	-0.09 (-0.18- -0.0002)	.049	.11
Medial and inferior temporal gyri anterior part right white matter	-0.11 (-0.19- -0.02)	.02	.051
Occipital lobe left gray matter	-0.06 (-0.12- -0.002)	.04	.10
SIMD			
Amygdala left	0.04 (-0.07-0.16)	.47	.59
Amygdala right	-0.06 (-0.18-0.06)	.33	.45
Anterior temporal lobe lateral part left gray matter	-0.01 (-0.11-0.08)	.78	.83
Anterior temporal lobe lateral part left white matter	0.05 (-0.06-0.16)	.37	.49
Anterior temporal lobe lateral part right gray matter	0.02 (-0.08-0.12)	.69	.77
Anterior temporal lobe lateral part right white matter	-0.03 (-0.14-0.09)	.61	.71
Anterior temporal lobe medial part left gray matter	0.00002 (-0.11-0.11)	>.99	>.99
Anterior temporal lobe medial part left white matter	0.03 (-0.10-0.15)	.67	.75
Anterior temporal lobe medial part right gray matter	-0.02 (-0.13-0.09)	.76	.83
Anterior temporal lobe medial part right white matter	0.07 (-0.05-0.19)	.28	.40
Brainstem	0.11 (0.004-0.22)	.04	.10
Caudate nucleus left	0.11 (0.002-0.21)	.04	.10
Caudate nucleus right	0.12 (0.01-0.23)	.04	.10
Cerebellum left	0.06 (-0.03-0.15)	.18	.30
Cerebellum right	0.08 (-0.01-0.16)	.09	.18
Cerebrospinal fluid	0.46 (0.16-0.76)	.003	.01

© 2023 Mckinnon K et al. *JAMA Network Open.*

Regional volume	Standardized β coefficient (95% confidence intervals)	Raw *p* value	BH corrected *p* value
Cingulate gyrus anterior part left gray matter	0.001 (-0.12-0.12)	.99	>.99
Cingulate gyrus anterior part left white matter	0.08 (-0.05-0.20)	.23	.37
Cingulate gyrus anterior part right gray matter	-0.05 (-0.17-0.07)	.39	.51
Cingulate gyrus anterior part right white matter	0.06 (-0.06-0.18)	.35	.47
Cingulate gyrus posterior part left gray matter	0.02 (-0.08-0.13)	.65	.75
Cingulate gyrus posterior part left white matter	0.17 (0.06-0.28)	.003	.01
Cingulate gyrus posterior part right gray matter	0.01 (-0.10-0.13)	.83	.87
Cingulate gyrus posterior part right white matter	0.11 (0.001-0.23)	.047	.11
Corpus callosum	0.43 (0.14-0.73)	.004	.02
Frontal lobe left gray matter	0.03 (-0.07-0.13)	.53	.65
Frontal lobe left white matter	0.07 (-0.03-0.18)	.18	.31
Frontal lobe right gray matter	-0.02 (-0.11-0.08)	.75	.82
Frontal lobe right white matter	0.13 (0.02-0.24)	.02	.053
Gyri parahippocampalis et ambiens anterior part left gray matter	0.02 (-0.10-0.14)	.75	.82
Gyri parahippocampalis et ambiens anterior part left white matter	-0.28 (-0.58-0.02)	.06	.13
Gyri parahippocampalis et ambiens anterior part right gray matter	-0.04 (-0.16-0.08)	.46	.59
Gyri parahippocampalis et ambiens anterior part right white matter	0.06 (-0.06-0.17)	.32	.45
Gyri parahippocampalis et ambiens posterior part left gray matter	0.10 (-0.01-0.21)	.06	.13
Gyri parahippocampalis et ambiens posterior part left white matter	0.07 (-0.05-0.19)	.25	.39
Gyri parahippocampalis et ambiens posterior part right gray matter	0.03 (-0.08-0.14)	.61	.71
Gyri parahippocampalis et ambiens posterior part right white matter	0.01 (-0.11-0.12)	.93	.95
Hippocampus left	0.08 (-0.04-0.20)	.20	.33
Hippocampus right	0.06 (-0.06-0.19)	.30	.43
Insula left gray matter	-0.03 (-0.13-0.08)	.60	.71
Insula left white matter	0.13 (0.02-0.25)	.02	.07
Insula right gray matter	-0.01 (-0.11-0.09)	.86	.89
Insula right white matter	0.12 (0.01-0.23)	.04	.10
Lateral occipitotemporal gyrus gyrus fusiformis anterior part left gray matter	0.09 (-0.02-0.19)	.11	.21
Lateral occipitotemporal gyrus gyrus fusiformis anterior part left white matter	0.12 (0.0003-0.24)	.049	.11
Lateral occipitotemporal gyrus gyrus fusiformis anterior part right gray matter	0.05 (-0.05-0.16)	.30	.42
Lateral occipitotemporal gyrus gyrus fusiformis anterior part right white matter	0.14 (0.02-0.26)	.02	.06
Lateral occipitotemporal gyrus gyrus fusiformis posterior part left gray matter	0.08 (-0.03-0.18)	.15	.28
Lateral occipitotemporal gyrus gyrus fusiformis posterior part left white matter	0.07 (-0.05-0.19)	.24	.37
Lateral occipitotemporal gyrus gyrus fusiformis posterior part right gray matter	0.06 (-0.05-0.17)	.27	.40
Lateral occipitotemporal gyrus gyrus fusiformis posterior part right white matter	0.09 (-0.03-0.21)	.15	.28

© 2023 Mckinnon K et al. *JAMA Network Open.*

Regional volume	Standardized β coefficient (95% confidence intervals)	Raw *p* value	BH corrected *p* value
Lateral ventricle left	0.44 (0.11-0.76)	.01	.03
Lateral ventricle right	0.11 (-0.01-0.23)	.09	.17
Lentiform nucleus left	-0.07 (-0.17-0.03)	.19	.31
Lentiform nucleus right	-0.10 (-0.20- -0.01)	.04	.09
Medial and inferior temporal gyri anterior part left gray matter	0.06 (-0.02-0.15)	.14	.25
Medial and inferior temporal gyri anterior part left white matter	0.10 (-0.02-0.21)	.11	.21
Medial and inferior temporal gyri anterior part right gray matter	0.04 (-0.03-0.12)	.27	.40
Medial and inferior temporal gyri anterior part right white matter	0.47 (0.14-0.79)	.005	.02
Medial and inferior temporal gyri posterior part left gray matter	0.03 (-0.05-0.11)	.42	.55
Medial and inferior temporal gyri posterior part left white matter	0.03 (-0.10-0.15)	.66	.75
Medial and inferior temporal gyri posterior part right gray matter	0.03 (-0.05-0.10)	.49	.61
Medial and inferior temporal gyri posterior part right white matter	0.10 (-0.02-0.22)	.11	.21
Occipital lobe left gray matter	0.21 (-0.01-0.43)	.06	.13
Occipital lobe left white matter	0.04 (-0.09-0.17)	.53	.65
Occipital lobe right gray matter	0.01 (-0.08-0.10)	.82	.86
Occipital lobe right white matter	0.05 (-0.08-0.17)	.46	.59
Parietal lobe left gray matter	0.02 (-0.05-0.10)	.53	.65
Parietal lobe left white matter	0.07 (-0.05-0.19)	.23	.37
Parietal lobe right gray matter	0.02 (-0.06-0.09)	.66	.75
Parietal lobe right white matter	0.07 (-0.05-0.19)	.24	.37
Subthalamic nucleus left	-0.07 (-0.19-0.05)	.27	.40
Subthalamic nucleus right	0.04 (-0.07-0.16)	.49	.61
Superior temporal gyrus middle part left gray matter	0.02 (-0.08-0.12)	.73	.80
Superior temporal gyrus middle part left white matter	0.02 (-0.11-0.14)	.80	.85
Superior temporal gyrus middle part right gray matter	0.01 (-0.08-0.09)	.85	.88
Superior temporal gyrus middle part right white matter	0.10 (-0.02-0.22)	.11	.21
Superior temporal gyrus posterior part left gray matter	0.06 (-0.04-0.16)	.23	.37
Superior temporal gyrus posterior part left white matter	0.08 (-0.04-0.21)	.19	.32
Superior temporal gyrus posterior part right gray matter	0.07 (-0.03-0.17)	.15	.28
Superior temporal gyrus posterior part right white matter	0.06 (-0.07-0.18)	.36	.49
Thalamus left high intensity part in T2	-0.09 (-0.19-0.004)	.06	.13
Thalamus left low intensity part in T2	0.04 (-0.09-0.16)	.58	.70
Thalamus right high intensity part in T2	-0.07 (-0.17-0.03)	.16	.28
Thalamus right low intensity part in T2	-0.06 (-0.17-0.06)	.31	.44

Baseline unadjusted linear regression model, including gestation at birth, SIMD, gestation at MRI, and the interaction term (where significant). Corrected for false discovery rate.
BH = Benjamini-Hochberg correction, SIMD = Scottish Index of Multiple Deprivation.

© 2023 Mckinnon K et al. *JAMA Network Open.*

eTable 5. Cortical Measures: Association With Gestational Age and the Scottish Index of Multiple Deprivation (Baseline Unadjusted Linear Regression Model)

Cortical measure	Standardized β coefficient (95% confidence intervals)	Raw *p* value	BH corrected *p* value
Gestational age			
Mean cortical curvature	-0.02 (-0.09-0.05)	.57	.57
Mean cortical surface area	0.19 (0.11-0.26)	<.001	<.001
Mean cortical thickness	-0.07 (-0.16-0.02)	.15	.36
Mean gyrification index	0.18 (0.10-0.26)	<.001	<.001
Mean sulcal depth	0.08 (-0.02-0.18)	.11	.36
SIMD			
Mean cortical curvature	-0.06 (-0.15-0.04)	.22	.36
Mean cortical surface area	0.05 (-0.05-0.15)	.29	.42
Mean cortical thickness	-0.08 (-0.19-0.04)	.37	.46
Mean gyrification index	0.05 (-0.06-0.15)	.22	.36
Mean sulcal depth	0.05 (-0.08-0.17)	.46	.51

Baseline unadjusted linear regression model, including gestation at birth, SIMD, gestation at MRI, and the interaction term (where significant – none were, so not included). Corrected for false discovery rate.
BH = Benjamini-Hochberg correction, SIMD = Scottish Index of Multiple Deprivation.

© 2023 Mckinnon K et al. *JAMA Network Open.*

eTable 6. Regional Brain Volumes: Association With Gestational Age, Maternal Final Educational Qualification, and Interaction Effect (Fully Adjusted Ridge Regression Model)

Regional volume	Standardized β coefficient (95% confidence intervals)	Raw *p* value	BH corrected *p* value
Gestation			
Amygdala left	0.03 (-0.29-0.34)	.53	.66
Amygdala right	0.01 (-0.08-0.11)	.74	.83
Anterior temporal lobe lateral part left gray matter	0.16 (0.08-0.24)	<.001	.002
Anterior temporal lobe lateral part left white matter	0.11 (0.01-0.21)	.01	.07
Anterior temporal lobe lateral part right gray matter	0.07 (-0.02-0.15)	.10	.26
Anterior temporal lobe lateral part right white matter	0.10 (0.0003-0.21)	.02	.09
Anterior temporal lobe medial part left gray matter	0.04 (-0.06-0.13)	.39	.58
Anterior temporal lobe medial part left white matter	0.03 (-0.08-0.14)	.46	.61
Anterior temporal lobe medial part right gray matter	0.04 (-0.06-0.14)	.37	.56
Anterior temporal lobe medial part right white matter	0.04 (-0.06-0.15)	.35	.54
Brainstem	-0.02 (-0.11-0.07)	.63	.73
Caudate nucleus left	0.07 (-0.21-0.36)	.08	.22
Caudate nucleus right	0.07 (-0.24-0.37)	.08	.23
Cerebellum left	-0.04 (-0.11-0.03)	.23	.41
Cerebellum right	-0.06 (-0.12-0.01)	.10	.25
Cerebrospinal fluid	-0.15 (-0.46-0.16)	<.001	<.001
Cingulate gyrus anterior part left gray matter	0.03 (-0.07-0.12)	.53	.66
Cingulate gyrus anterior part left white matter	0.07 (-0.03-0.18)	.07	.20
Cingulate gyrus anterior part right gray matter	-0.06 (-0.16-0.04)	.16	.34
Cingulate gyrus anterior part right white matter	0.04 (-0.06-0.15)	.28	.47
Cingulate gyrus posterior part left gray matter	-0.07 (-0.15-0.02)	.11	.28
Cingulate gyrus posterior part left white matter	0.05 (-0.03-0.14)	.19	.36
Cingulate gyrus posterior part right gray matter	-0.10 (-0.20- -0.01)	.02	.09
Cingulate gyrus posterior part right white matter	0.08 (-0.01-0.17)	.047	.16
Corpus callosum	0.08 (-0.02-0.18)	.06	.19
Frontal lobe left gray matter	0.02 (-0.06-0.10)	.61	.73
Frontal lobe left white matter	0.20 (0.12-0.29)	<.001	<.001
Frontal lobe right gray matter	0.02 (-0.06-0.09)	.62	.73
Frontal lobe right white matter	0.17 (0.09-0.26)	<.001	<.001
Gyri parahippocampalis et ambiens anterior part left gray matter	0.08 (-0.02-0.19)	.046	.16
Gyri parahippocampalis et ambiens anterior part left white matter	0.08 (-0.24-0.40)	.04	.16
Gyri parahippocampalis et ambiens anterior part right gray matter	0.09 (-0.01-0.19)	.048	.16
Gyri parahippocampalis et ambiens anterior part right white matter	0.03 (-0.29-0.35)	.49	.63
Gyri parahippocampalis et ambiens posterior part left gray matter	0.14 (0.04-0.24)	.002	.02
Gyri parahippocampalis et ambiens posterior part left white matter	0.04 (-0.31-0.38)	.18	.36

© 2023 Mckinnon K et al. *JAMA Network Open.*

Regional volume	Standardized β coefficient (95% confidence intervals)	Raw *p* value	BH corrected *p* value
Gyri parahippocampalis et ambiens posterior part right gray matter	0.05 (-0.05-0.15)	.29	.47
Gyri parahippocampalis et ambiens posterior part right white matter	0.13 (0.03-0.24)	.002	.02
Hippocampus left	-0.08 (-0.18-0.02)	.06	.18
Hippocampus right	-0.04 (-0.15-0.07)	.30	.48
Insula left gray matter	0.03 (-0.05-0.11)	.44	.60
Insula left white matter	0.15 (0.06-0.24)	<.001	.004
Insula right gray matter	-0.04 (-0.12-0.04)	.32	.50
Insula right white matter	0.15 (0.06-0.24)	<.001	.004
Lateral occipitotemporal gyrus gyrus fusiformis anterior part left gray matter	0.16 (-0.14-0.46)	<.001	.004
Lateral occipitotemporal gyrus gyrus fusiformis anterior part left white matter	-0.10 (-0.20- -0.003)	.03	.11
Lateral occipitotemporal gyrus gyrus fusiformis anterior part right gray matter	0.09 (-0.19-0.37)	.08	.22
Lateral occipitotemporal gyrus gyrus fusiformis anterior part right white matter	-0.04 (-0.14-0.06)	.40	.59
Lateral occipitotemporal gyrus gyrus fusiformis posterior part left gray matter	0.11 (0.02-0.21)	.01	.06
Lateral occipitotemporal gyrus gyrus fusiformis posterior part left white matter	-0.07 (-0.17-0.02)	.10	.26
Lateral occipitotemporal gyrus gyrus fusiformis posterior part right gray matter	0.03 (-0.29-0.34)	.55	.68
Lateral occipitotemporal gyrus gyrus fusiformis posterior part right white matter	-0.01 (-0.12-0.09)	.75	.83
Lateral ventricle left	-0.08 (-0.42-0.25)	.002	.02
Lateral ventricle right	-0.08 (-0.41-0.24)	<.001	.01
Lentiform nucleus left	-0.08 (-0.17-0.004)	.047	.16
Lentiform nucleus right	-0.11 (-0.19- -0.02)	.01	.06
Medial and inferior temporal gyri anterior part left gray matter	0.12 (-0.11-0.35)	.03	.11
Medial and inferior temporal gyri anterior part left white matter	0.03 (-0.06-0.13)	.43	.60
Medial and inferior temporal gyri anterior part right gray matter	0.23 (0.16-0.29)	<.001	<.001
Medial and inferior temporal gyri anterior part right white matter	0.06 (-0.26-0.38)	.20	.38
Medial and inferior temporal gyri posterior part left gray matter	0.12 (0.06-0.19)	<.001	.004
Medial and inferior temporal gyri posterior part left white matter	-0.08 (-0.18-0.02)	.06	.19
Medial and inferior temporal gyri posterior part right gray matter	0.002 (-0.21-0.22)	.97	.98
Medial and inferior temporal gyri posterior part right white matter	-0.05 (-0.15-0.05)	.25	.43
Occipital lobe left gray matter	-0.03 (-0.10-0.04)	.42	.60
Occipital lobe left white matter	-0.08 (-0.18-0.02)	.08	.22
Occipital lobe right gray matter	0.002 (-0.07-0.08)	.97	.98
Occipital lobe right white matter	-0.07 (-0.17-0.04)	.15	.33
Parietal lobe left gray matter	0.06 (0.01-0.12)	.02	.10
Parietal lobe left white matter	0.10 (0.003-0.19)	.02	.10
Parietal lobe right gray matter	0.08 (0.02-0.14)	.004	.04

© 2023 Mckinnon K et al. *JAMA Network Open.*

Regional volume	Standardized β coefficient (95% confidence intervals)	Raw *p* value	BH corrected *p* value
Parietal lobe right white matter	0.06 (-0.04-0.15)	.19	.37
Subthalamic nucleus left	-0.08 (-0.31-0.16)	.051	.16
Subthalamic nucleus right	-0.08 (-0.18-0.01)	.02	.09
Superior temporal gyrus middle part left gray matter	0.09 (0.01-0.18)	.02	.09
Superior temporal gyrus middle part left white matter	0.03 (-0.07-0.13)	.42	.60
Superior temporal gyrus middle part right gray matter	0.11 (0.04-0.19)	.002	.02
Superior temporal gyrus middle part right white matter	0.04 (-0.06-0.14)	.39	.58
Superior temporal gyrus posterior part left gray matter	0.08 (-0.01-0.16)	.049	.16
Superior temporal gyrus posterior part left white matter	0.03 (-0.07-0.13)	.43	.60
Superior temporal gyrus posterior part right gray matter	0.04 (-0.05-0.12)	.35	.54
Superior temporal gyrus posterior part right white matter	0.04 (-0.06-0.14)	.35	.54
Thalamus left high intensity part in T2	-0.02 (-0.10-0.06)	.69	.78
Thalamus left low intensity part in T2	0.09 (-0.03-0.20)	.051	.16
Thalamus right high intensity part in T2	-0.05 (-0.13-0.03)	.23	.41
Thalamus right low intensity part in T2	0.02 (-0.08-0.12)	.60	.73
Interaction			
Amygdala left	0.01 (-0.04-0.07)	.93	.96
Caudate nucleus left	0.02 (-0.03-0.07)	.004	.03
Caudate nucleus right	0.01 (-0.04-0.07)	.01	.06
Cerebrospinal fluid	-0.01 (-0.07-0.04)	<.001	<.001
Gyri parahippocampalis et ambiens anterior part left white matter	0.01 (-0.04-0.07)	.01	.04
Gyri parahippocampalis et ambiens anterior part right white matter	0.01 (-0.05-0.07)	.04	.14
Gyri parahippocampalis et ambiens posterior part left white matter	0.01 (-0.05-0.07)	.04	.16
Lateral occipitotemporal gyrus gyrus fusiformis anterior part left gray matter	0.02 (-0.04-0.07)	.01	.06
Lateral occipitotemporal gyrus gyrus fusiformis anterior part right gray matter	0.02 (-0.03-0.07)	.01	.08
Lateral occipitotemporal gyrus gyrus fusiformis posterior part right gray matter	0.01 (-0.04-0.07)	.02	.09
Lateral ventricle left	-0.01 (-0.07-0.05)	.001	.02
Lateral ventricle right	-0.01 (-0.06-0.05)	.003	.03
Medial and inferior temporal gyri anterior part left gray matter	0.02 (-0.02-0.06)	.01	.07
Medial and inferior temporal gyri anterior part right white matter	-0.01 (-0.07-0.04)	.03	.11
Medial and inferior temporal gyri posterior part right gray matter	0.02 (-0.01-0.06)	.02	.09
Subthalamic nucleus left	-0.01 (-0.05-0.03)	.04	.16
Maternal education			
Amygdala left	0.00 (-0.21-0.21)	.02	.10
Amygdala right	0.08 (-0.01-0.18)	.049	.16
Anterior temporal lobe lateral part left gray matter	-0.003 (-0.09-0.08)	.93	.96
Anterior temporal lobe lateral part left white matter	0.05 (-0.05-0.15)	.21	.39
Anterior temporal lobe lateral part right gray matter	0.03 (-0.06-0.11)	.47	.62

© 2023 Mckinnon K et al. *JAMA Network Open.*

Regional measure	Standardized β coefficient (95% confidence intervals)	Raw *p* value	BH corrected *p* value
Anterior temporal lobe lateral part right white matter	0.01 (-0.09-0.11)	.77	.84
Anterior temporal lobe medial part left gray matter	0.04 (-0.06-0.13)	.36	.54
Anterior temporal lobe medial part left white matter	0.01 (-0.10-0.12)	.82	.88
Anterior temporal lobe medial part right gray matter	0.08 (-0.02-0.17)	.07	.21
Anterior temporal lobe medial part right white matter	0.02 (-0.09-0.12)	.65	.75
Brainstem	0.03 (-0.06-0.12)	.41	.60
Caudate nucleus left	0.03 (-0.16-0.22)	.49	.63
Caudate nucleus right	-0.02 (-0.22-0.18)	.61	.73
Cerebellum left	0.11 (0.04-0.18)	.001	.02
Cerebellum right	0.09 (0.02-0.16)	.01	.049
Cerebrospinal fluid	0.05 (-0.16-0.26)	.02	.09
Cingulate gyrus anterior part left gray matter	-0.05 (-0.14-0.05)	.23	.41
Cingulate gyrus anterior part left white matter	0.05 (-0.05-0.16)	.14	.31
Cingulate gyrus anterior part right gray matter	-0.05 (-0.14-0.05)	.22	.41
Cingulate gyrus anterior part right white matter	0.05 (-0.05-0.16)	.15	.32
Cingulate gyrus posterior part left gray matter	-0.01 (-0.10-0.07)	.74	.83
Cingulate gyrus posterior part left white matter	0.05 (-0.03-0.14)	.16	.34
Cingulate gyrus posterior part right gray matter	0.001 (-0.09-0.09)	.99	.99
Cingulate gyrus posterior part right white matter	0.03 (-0.06-0.12)	.44	.60
Corpus callosum	0.06 (-0.04-0.15)	.16	.34
Frontal lobe left gray matter	-0.01 (-0.08-0.07)	.88	.93
Frontal lobe left white matter	0.06 (-0.03-0.14)	.11	.28
Frontal lobe right gray matter	0.01 (-0.07-0.08)	.85	.91
Frontal lobe right white matter	0.04 (-0.05-0.13)	.31	.50
Gyri parahippocampalis et ambiens anterior part left gray matter	-0.02 (-0.13-0.09)	.62	.73
Gyri parahippocampalis et ambiens anterior part left white matter	0.02 (-0.19-0.24)	.52	.66
Gyri parahippocampalis et ambiens anterior part right gray matter	0.01 (-0.09-0.11)	.78	.85
Gyri parahippocampalis et ambiens anterior part right white matter	0.03 (-0.18-0.25)	.41	.60
Gyri parahippocampalis et ambiens posterior part left gray matter	0.02 (-0.08-0.12)	.58	.71
Gyri parahippocampalis et ambiens posterior part left white matter	0.03 (-0.20-0.26)	.30	.48
Gyri parahippocampalis et ambiens posterior part right gray matter	0.01 (-0.09-0.11)	.89	.94
Gyri parahippocampalis et ambiens posterior part right white matter	0.03 (-0.07-0.13)	.42	.60
Hippocampus left	0.06 (-0.04-0.16)	.14	.32
Hippocampus right	0.05 (-0.05-0.16)	.13	.31
Insula left gray matter	-0.003 (-0.08-0.08)	.94	.96
Insula left white matter	0.10 (0.01-0.19)	.01	.06
Insula right gray matter	0.03 (-0.05-0.11)	.47	.62
Insula right white matter	0.05 (-0.04-0.14)	.22	.41

© 2023 Mckinnon K et al. *JAMA Network Open.*

Regional volume	Standardized β coefficient (95% confidence intervals)	Raw *p* value	BH corrected *p* value
Lateral occipitotemporal gyrus gyrus fusiformis anterior part left gray matter	-0.04 (-0.24-0.16)	.28	.47
Lateral occipitotemporal gyrus gyrus fusiformis anterior part left white matter	0.15 (0.05-0.25)	<.001	.01
Lateral occipitotemporal gyrus gyrus fusiformis anterior part right gray matter	-0.02 (-0.21-0.17)	.64	.74
Lateral occipitotemporal gyrus gyrus fusiformis anterior part right white matter	0.10 (0.001-0.20)	.02	.09
Lateral occipitotemporal gyrus gyrus fusiformis posterior part left gray matter	0.03 (-0.07-0.13)	.49	.63
Lateral occipitotemporal gyrus gyrus fusiformis posterior part left white matter	0.05 (-0.05-0.15)	.24	.41
Lateral occipitotemporal gyrus gyrus fusiformis posterior part right gray matter	-0.003 (-0.21-0.20)	.93	.96
Lateral occipitotemporal gyrus gyrus fusiformis posterior part right white matter	0.02 (-0.08-0.12)	.59	.72
Lateral ventricle left	0.04 (-0.18-0.26)	.09	.25
Lateral ventricle right	0.04 (-0.18-0.25)	.10	.26
Lentiform nucleus left	-0.01 (-0.09-0.08)	.90	.95
Lentiform nucleus right	0.03 (-0.05-0.11)	.42	.60
Medial and inferior temporal gyri anterior part left gray matter	-0.07 (-0.22-0.09)	.12	.28
Medial and inferior temporal gyri anterior part left white matter	0.07 (-0.02-0.16)	.08	.23
Medial and inferior temporal gyri anterior part right gray matter	0.02 (-0.05-0.08)	.63	.73
Medial and inferior temporal gyri anterior part right white matter	0.08 (-0.13-0.29)	.053	.17
Medial and inferior temporal gyri posterior part left gray matter	-0.02 (-0.09-0.05)	.51	.65
Medial and inferior temporal gyri posterior part left white matter	0.06 (-0.04-0.16)	.16	.34
Medial and inferior temporal gyri posterior part right gray matter	-0.05 (-0.19-0.09)	.28	.47
Medial and inferior temporal gyri posterior part right white matter	0.05 (-0.05-0.15)	.23	.41
Occipital lobe left gray matter	0.05 (-0.02-0.11)	.18	.36
Occipital lobe left white matter	0.02 (-0.08-0.13)	.58	.71
Occipital lobe right gray matter	0.05 (-0.02-0.12)	.15	.33
Occipital lobe right white matter	0.06 (-0.04-0.16)	.14	.32
Parietal lobe left gray matter	0.04 (-0.02-0.09)	.18	.36
Parietal lobe left white matter	0.03 (-0.06-0.12)	.44	.60
Parietal lobe right gray matter	0.03 (-0.02-0.09)	.24	.41
Parietal lobe right white matter	0.03 (-0.06-0.12)	.45	.61
Subthalamic nucleus left	0.06 (-0.09-0.21)	.10	.26
Subthalamic nucleus right	0.04 (-0.05-0.14)	.20	.38
Superior temporal gyrus middle part left gray matter	0.01 (-0.07-0.09)	.80	.86
Superior temporal gyrus middle part left white matter	0.11 (0.01-0.21)	.01	.048
Superior temporal gyrus middle part right gray matter	0.01 (-0.06-0.08)	.74	.83
Superior temporal gyrus middle part right white matter	0.06 (-0.04-0.16)	.12	.29
Superior temporal gyrus posterior part left gray matter	-0.01 (-0.09-0.07)	.75	.83
Superior temporal gyrus posterior part left white matter	0.04 (-0.06-0.14)	.23	.41

© 2023 Mckinnon K et al. *JAMA Network Open.*

Regional volume	Standardized β coefficient (95% confidence intervals)	Raw *p* value	BH corrected *p* value
Superior temporal gyrus posterior part right gray matter	0.04 (-0.04-0.12)	.32	.51
Superior temporal gyrus posterior part right white matter	0.002 (-0.10-0.10)	.96	.98
Thalamus left high intensity part in T2	0.03 (-0.05-0.11)	.45	.60
Thalamus left low intensity part in T2	-0.06 (-0.17-0.06)	.18	.36
Thalamus right high intensity part in T2	0.01 (-0.07-0.10)	.70	.80
Thalamus right low intensity part in T2	-0.004 (-0.10-0.09)	.92	.96

Fully adjusted ridge regression model, including gestation at birth, maternal final educational qualification, gestation at MRI, the interaction term (where significant), birth weight z-score, birth head circumference z-score, sex, smoking in pregnancy, and breast milk at discharge. Corrected for false discovery rate.
BH = Benjamini-Hochberg correction.

© 2023 Mckinnon K et al. *JAMA Network Open.*

eTable 7. Regional Brain Volumes: Association With Gestational Age, Paternal Final Educational Qualification, and Interaction Effect (Fully Adjusted Ridge Regression Model)

Regional volume	Standardized β coefficient (95% confidence intervals)	Raw *p* value	BH corrected *p* value
Gestation			
Amygdala left	0.07 (-0.23-0.37)	.07	.22
Amygdala right	0.04 (-0.06-0.14)	.37	.61
Anterior temporal lobe lateral part left gray matter	0.13 (0.04-0.23)	.002	.02
Anterior temporal lobe lateral part left white matter	0.10 (-0.01-0.20)	.04	.16
Anterior temporal lobe lateral part right gray matter	0.04 (-0.05-0.13)	.29	.51
Anterior temporal lobe lateral part right white matter	0.10 (-0.01-0.20)	.04	.16
Anterior temporal lobe medial part left gray matter	0.04 (-0.06-0.14)	.41	.61
Anterior temporal lobe medial part left white matter	0.03 (-0.08-0.15)	.38	.61
Anterior temporal lobe medial part right gray matter	0.03 (-0.07-0.13)	.47	.67
Anterior temporal lobe medial part right white matter	0.05 (-0.06-0.16)	.24	.50
Brainstem	-0.01 (-0.10-0.09)	.90	.93
Caudate nucleus left	0.11 (-0.16-0.38)	.004	.03
Caudate nucleus right	0.15 (0.06-0.25)	<.001	.004
Cerebellum left	-0.02 (-0.10-0.06)	.61	.75
Cerebellum right	-0.04 (-0.11-0.04)	.31	.54
Cerebrospinal fluid	-0.20 (-0.51-0.11)	<.001	<.001
Cingulate gyrus anterior part left gray matter	0.03 (-0.07-0.13)	.41	.61
Cingulate gyrus anterior part left white matter	0.04 (-0.27-0.36)	.18	.43
Cingulate gyrus anterior part right gray matter	-0.04 (-0.14-0.06)	.32	.54
Cingulate gyrus anterior part right white matter	0.06 (-0.05-0.17)	.12	.33
Cingulate gyrus posterior part left gray matter	-0.07 (-0.16-0.03)	.13	.34
Cingulate gyrus posterior part left white matter	0.05 (-0.04-0.15)	.20	.44
Cingulate gyrus posterior part right gray matter	-0.11 (-0.21- -0.01)	.01	.08
Cingulate gyrus posterior part right white matter	0.08 (-0.01-0.18)	.051	.17
Corpus callosum	0.07 (-0.03-0.17)	.09	.27
Frontal lobe left gray matter	0.03 (-0.06-0.11)	.51	.70
Frontal lobe left white matter	0.22 (0.13-0.31)	<.001	<.001
Frontal lobe right gray matter	0.03 (-0.05-0.11)	.39	.61
Frontal lobe right white matter	0.18 (0.09-0.27)	<.001	<.001
Gyri parahippocampalis et ambiens anterior part left gray matter	0.05 (-0.28-0.39)	.15	.38
Gyri parahippocampalis et ambiens anterior part left white matter	0.11 (-0.20-0.41)	.01	.047
Gyri parahippocampalis et ambiens anterior part right gray matter	0.04 (-0.27-0.35)	.32	.54
Gyri parahippocampalis et ambiens anterior part right white matter	0.03 (-0.28-0.33)	.45	.65
Gyri parahippocampalis et ambiens posterior part left gray matter	0.14 (0.03-0.24)	.002	.02
Gyri parahippocampalis et ambiens posterior part left white matter	0.07 (-0.04-0.19)	.049	.17

© 2023 Mckinnon K et al. *JAMA Network Open.*

Regional volume	Standardized β coefficient (95% confidence intervals)	Raw *p* value	BH corrected *p* value
Gyri parahippocampalis et ambiens posterior part right gray matter	0.05 (-0.05-0.16)	.25	.50
Gyri parahippocampalis et ambiens posterior part right white matter	0.15 (0.04-0.25)	<.001	.01
Hippocampus left	-0.04 (-0.15-0.06)	.25	.50
Hippocampus right	-0.02 (-0.13-0.09)	.56	.74
Insula left gray matter	0.04 (-0.05-0.12)	.34	.57
Insula left white matter	0.10 (-0.18-0.38)	.02	.11
Insula right gray matter	-0.02 (-0.10-0.06)	.62	.76
Insula right white matter	0.12 (-0.16-0.40)	.004	.03
Lateral occipitotemporal gyrus gyrus fusiformis anterior part left gray matter	0.24 (0.14-0.34)	<.001	<.001
Lateral occipitotemporal gyrus gyrus fusiformis anterior part left white matter	-0.07 (-0.18-0.03)	.10	.29
Lateral occipitotemporal gyrus gyrus fusiformis anterior part right gray matter	0.09 (-0.18-0.36)	.06	.20
Lateral occipitotemporal gyrus gyrus fusiformis anterior part right white matter	-0.04 (-0.35-0.27)	.27	.50
Lateral occipitotemporal gyrus gyrus fusiformis posterior part left gray matter	0.13 (0.03-0.23)	.004	.03
Lateral occipitotemporal gyrus gyrus fusiformis posterior part left white matter	-0.03 (-0.13-0.07)	.52	.70
Lateral occipitotemporal gyrus gyrus fusiformis posterior part right gray matter	0.09 (-0.01-0.19)	.04	.17
Lateral occipitotemporal gyrus gyrus fusiformis posterior part right white matter	0.01 (-0.09-0.12)	.69	.81
Lateral ventricle left	-0.09 (-0.41-0.23)	<.001	.004
Lateral ventricle right	-0.09 (-0.39-0.22)	<.001	.01
Lentiform nucleus left	-0.09 (-0.18-0.002)	.04	.17
Lentiform nucleus right	-0.12 (-0.21- -0.04)	.003	.03
Medial and inferior temporal gyri anterior part left gray matter	0.23 (0.16-0.31)	<.001	<.001
Medial and inferior temporal gyri anterior part left white matter	0.05 (-0.06-0.15)	.29	.51
Medial and inferior temporal gyri anterior part right gray matter	0.23 (0.16-0.30)	<.001	<.001
Medial and inferior temporal gyri anterior part right white matter	0.02 (-0.08-0.13)	.58	.74
Medial and inferior temporal gyri posterior part left gray matter	0.12 (0.05-0.19)	<.001	.01
Medial and inferior temporal gyri posterior part left white matter	-0.03 (-0.14-0.07)	.41	.61
Medial and inferior temporal gyri posterior part right gray matter	0.13 (0.06-0.20)	<.001	.002
Medial and inferior temporal gyri posterior part right white matter	-0.01 (-0.12-0.09)	.72	.82
Occipital lobe left gray matter	-0.02 (-0.09-0.05)	.57	.74
Occipital lobe left white matter	-0.04 (-0.14-0.06)	.34	.57
Occipital lobe right gray matter	0.01 (-0.07-0.09)	.72	.82
Occipital lobe right white matter	-0.02 (-0.13-0.08)	.60	.75
Parietal lobe left gray matter	0.06 (-0.001-0.12)	.047	.17
Parietal lobe left white matter	0.13 (0.03-0.23)	.002	.02
Parietal lobe right gray matter	0.09 (0.03-0.15)	.004	.03

© 2023 Mckinnon K et al. *JAMA Network Open.*

Regional volume	Standardized β coefficient (95% confidence intervals)	Raw *p* value	BH corrected *p* value
Parietal lobe right white matter	0.09 (-0.01-0.19)	.04	.16
Subthalamic nucleus left	-0.07 (-0.22-0.09)	.048	.17
Subthalamic nucleus right	-0.03 (-0.31-0.24)	.16	.40
Superior temporal gyrus middle part left gray matter	0.09 (-0.002-0.17)	.04	.16
Superior temporal gyrus middle part left white matter	0.06 (-0.05-0.16)	.18	.42
Superior temporal gyrus middle part right gray matter	0.10 (0.02-0.18)	.01	.047
Superior temporal gyrus middle part right white matter	0.06 (-0.05-0.16)	.17	.42
Superior temporal gyrus posterior part left gray matter	0.08 (-0.01-0.17)	.06	.20
Superior temporal gyrus posterior part left white matter	0.04 (-0.07-0.15)	.34	.57
Superior temporal gyrus posterior part right gray matter	0.03 (-0.06-0.12)	.43	.64
Superior temporal gyrus posterior part right white matter	0.04 (-0.06-0.15)	.33	.56
Thalamus left high intensity part in T2	-0.002 (-0.09-0.08)	.96	.97
Thalamus left low intensity part in T2	0.005 (-0.004-0.01)	.25	.50
Thalamus right high intensity part in T2	-0.05 (-0.13-0.04)	.25	.50
Thalamus right low intensity part in T2	-0.01 (-0.11-0.10)	.88	.92
Interaction			
Amygdala left	0.01 (-0.05-0.06)	.04	.17
Caudate nucleus left	0.01 (-0.04-0.06)	.01	.049
Cerebrospinal fluid	-0.02 (-0.08-0.04)	<.001	<.001
Cingulate gyrus anterior part left white matter	0.01 (-0.05-0.07)	.048	.17
Gyri parahippocampalis et ambiens anterior part left gray matter	0.01 (-0.05-0.07)	.03	.16
Gyri parahippocampalis et ambiens anterior part left white matter	0.02 (-0.04-0.07)	.001	.01
Gyri parahippocampalis et ambiens anterior part right gray matter	0.01 (-0.05-0.07)	.04	.17
Gyri parahippocampalis et ambiens anterior part right white matter	0.02 (-0.04-0.07)	.001	.02
Insula left white matter	0.02 (-0.17-0.20)	.01	.06
Insula right white matter	0.01 (-0.04-0.06)	.04	.17
Lateral occipitotemporal gyrus gyrus fusiformis anterior part right gray matter	0.02 (-0.03-0.07)	.02	.12
Lateral occipitotemporal gyrus gyrus fusiformis anterior part right white matter	0.01 (-0.05-0.07)	.046	.17
Lateral ventricle left	-0.01 (-0.07-0.05)	.002	.02
Lateral ventricle right	-0.05 (-0.07-0.05)	.002	.02
Subthalamic nucleus left	-0.01 (-0.04-0.02)	.003	.03
Subthalamic nucleus right	-0.01 (-0.06-0.05)	.046	.17
Paternal education			
Amygdala left	-0.02 (-0.23-0.18)	.48	.67
Amygdala right	0.01 (-0.07-0.10)	.69	.81
Anterior temporal lobe lateral part left gray matter	-0.01 (-0.08-0.07)	.81	.88
Anterior temporal lobe lateral part left white matter	0.04 (-0.05-0.13)	.28	.50
Anterior temporal lobe lateral part right gray matter	0.05 (-0.03-0.12)	.17	.42

© 2023 Mckinnon K et al. *JAMA Network Open.*

Regional volume	Standardized β coefficient (95% confidence intervals)	Raw *p* value	BH corrected *p* value
Anterior temporal lobe lateral part right white matter	0.02 (-0.07-0.11)	.69	.81
Anterior temporal lobe medial part left gray matter	0.03 (-0.05-0.11)	.40	.61
Anterior temporal lobe medial part left white matter	0.01 (-0.09-0.10)	.84	.89
Anterior temporal lobe medial part right gray matter	0.04 (-0.04-0.13)	.28	.50
Anterior temporal lobe medial part right white matter	0.02 (-0.07-0.11)	.57	.74
Brainstem	0.01 (-0.06-0.09)	.69	.81
Caudate nucleus left	0.04 (-0.14-0.23)	.20	.44
Caudate nucleus right	0.08 (0.01-0.16)	.02	.09
Cerebellum left	0.06 (-0.004-0.12)	.054	.18
Cerebellum right	0.05 (-0.01-0.11)	.09	.27
Cerebrospinal fluid	0.04 (-0.17-0.25)	.19	.44
Cingulate gyrus anterior part left gray matter	0.002 (-0.08-0.09)	.94	.96
Cingulate gyrus anterior part left white matter	0.03 (-0.18-0.25)	.25	.50
Cingulate gyrus anterior part right gray matter	-0.01 (-0.10-0.07)	.63	.76
Cingulate gyrus anterior part right white matter	0.02 (-0.07-0.11)	.52	.70
Cingulate gyrus posterior part left gray matter	-0.01 (-0.08-0.07)	.83	.89
Cingulate gyrus posterior part left white matter	0.05 (-0.02-0.13)	.14	.36
Cingulate gyrus posterior part right gray matter	0.02 (-0.06-0.11)	.52	.70
Cingulate gyrus posterior part right white matter	0.02 (-0.06-0.10)	.57	.74
Corpus callosum	0.02 (-0.06-0.11)	.51	.70
Frontal lobe left gray matter	-0.01 (-0.08-0.05)	.67	.80
Frontal lobe left white matter	0.03 (-0.04-0.10)	.36	.58
Frontal lobe right gray matter	-0.02 (-0.08-0.05)	.58	.74
Frontal lobe right white matter	0.03 (-0.05-0.10)	.41	.61
Gyri parahippocampalis et ambiens anterior part left gray matter	-0.04 (-0.26-0.19)	.27	.50
Gyri parahippocampalis et ambiens anterior part left white matter	-0.07 (-0.29-0.14)	.03	.15
Gyri parahippocampalis et ambiens anterior part right gray matter	-0.03 (-0.24-0.18)	.44	.64
Gyri parahippocampalis et ambiens anterior part right white matter	-0.06 (-0.26-0.15)	.10	.28
Gyri parahippocampalis et ambiens posterior part left gray matter	0.01 (-0.07-0.10)	.73	.83
Gyri parahippocampalis et ambiens posterior part left white matter	0.02 (-0.07-0.11)	.52	.70
Gyri parahippocampalis et ambiens posterior part right gray matter	-0.0003 (-0.09-0.09)	.99	.99
Gyri parahippocampalis et ambiens posterior part right white matter	-0.02 (-0.11-0.07)	.59	.74
Hippocampus left	0.01 (-0.08-0.10)	.78	.88
Hippocampus right	0.01 (-0.09-0.10)	.79	.88
Insula left gray matter	-0.04 (-0.11-0.03)	.26	.50
Insula left white matter	0.02 (-0.04-0.07)	.67	.80
Insula right gray matter	-0.04 (-0.10-0.03)	.27	.50
Insula right white matter	0.001 (-0.19-0.19)	.97	.98

© 2023 Mckinnon K et al. *JAMA Network Open.*

Regional volume	Standardized β coefficient (95% confidence intervals)	Raw *p* value	BH corrected *p* value
Lateral occipitotemporal gyrus gyrus fusiformis anterior part left gray matter	0.03 (-0.05-0.11)	.38	.61
Lateral occipitotemporal gyrus gyrus fusiformis anterior part left white matter	0.08 (-0.01-0.17)	.03	.16
Lateral occipitotemporal gyrus gyrus fusiformis anterior part right gray matter	-0.01 (-0.19-0.17)	.79	.88
Lateral occipitotemporal gyrus gyrus fusiformis anterior part right white matter	0.05 (-0.16-0.26)	.10	.29
Lateral occipitotemporal gyrus gyrus fusiformis posterior part left gray matter	0.06 (-0.02-0.15)	.09	.27
Lateral occipitotemporal gyrus gyrus fusiformis posterior part left white matter	0.05 (-0.04-0.13)	.19	.44
Lateral occipitotemporal gyrus gyrus fusiformis posterior part right gray matter	0.06 (-0.02-0.14)	.09	.27
Lateral occipitotemporal gyrus gyrus fusiformis posterior part right white matter	0.04 (-0.05-0.13)	.21	.45
Lateral ventricle left	0.05 (-0.17-0.26)	.04	.16
Lateral ventricle right	-0.01 (-0.16-0.26)	.02	.10
Lentiform nucleus left	-0.003 (-0.08-0.07)	.94	.96
Lentiform nucleus right	0.004 (-0.07-0.07)	.91	.93
Medial and inferior temporal gyri anterior part left gray matter	0.03 (-0.03-0.09)	.26	.50
Medial and inferior temporal gyri anterior part left white matter	0.05 (-0.04-0.13)	.19	.44
Medial and inferior temporal gyri anterior part right gray matter	0.01 (-0.04-0.07)	.60	.75
Medial and inferior temporal gyri anterior part right white matter	-0.01 (-0.10-0.08)	.82	.88
Medial and inferior temporal gyri posterior part left gray matter	0.01 (-0.05-0.07)	.65	.79
Medial and inferior temporal gyri posterior part left white matter	0.05 (-0.04-0.14)	.14	.37
Medial and inferior temporal gyri posterior part right gray matter	0.05 (-0.004-0.11)	.06	.19
Medial and inferior temporal gyri posterior part right white matter	0.04 (-0.05-0.13)	.24	.50
Occipital lobe left gray matter	-0.004 (-0.06-0.06)	.88	.92
Occipital lobe left white matter	-0.03 (-0.12-0.06)	.41	.61
Occipital lobe right gray matter	0.01 (-0.06-0.07)	.81	.88
Occipital lobe right white matter	-0.03 (-0.12-0.06)	.46	.66
Parietal lobe left gray matter	0.02 (-0.03-0.07)	.49	.68
Parietal lobe left white matter	0.005 (-0.08-0.09)	.90	.93
Parietal lobe right gray matter	0.02 (-0.03-0.07)	.48	.68
Parietal lobe right white matter	-0.01 (-0.09-0.07)	.81	.88
Subthalamic nucleus left	0.01 (-0.09-0.11)	.83	.89
Subthalamic nucleus right	0.01 (-0.18-0.19)	.78	.88
Superior temporal gyrus middle part left gray matter	0.02 (-0.05-0.09)	.57	.74
Superior temporal gyrus middle part left white matter	0.04 (-0.04-0.13)	.20	.44
Superior temporal gyrus middle part right gray matter	0.05 (-0.01-0.11)	.11	.29
Superior temporal gyrus middle part right white matter	0.05 (-0.04-0.13)	.16	.41
Superior temporal gyrus posterior part left gray matter	-0.02 (-0.10- -0.10)	.50	.70
Superior temporal gyrus posterior part left white matter	0.04 (-0.05-0.13)	.27	.50

© 2023 Mckinnon K et al. *JAMA Network Open.*

Regional volume	Standardized β coefficient (95% confidence intervals)	Raw *p* value	BH corrected *p* value
Superior temporal gyrus posterior part right gray matter	0.04 (-0.03-0.12)	.23	.50
Superior temporal gyrus posterior part right white matter	-0.01 (-0.09-0.08)	.85	.90
Thalamus left high intensity part in T2	-0.04 (-0.10-0.03)	.29	.51
Thalamus left low intensity part in T2	0.001 (-0.01-0.01)	.71	.82
Thalamus right high intensity part in T2	-0.04 (-0.11-0.03)	.26	.50
Thalamus right low intensity part in T2	0.03 (-0.05-0.12)	.39	.61

Fully adjusted ridge regression model, including gestation at birth, paternal final educational qualification, gestation at MRI, the interaction term (where significant), birth weight z-score, birth head circumference z-score, sex, smoking in pregnancy, and breast milk at discharge. Corrected for false discovery rate.
BH = Benjamini-Hochberg correction.

© 2023 Mckinnon K et al. *JAMA Network Open.*

eTable 8. Regional Brain Volumes: Association With Gestational Age, Maternal Occupation, and Interaction Effect (Fully Adjusted Ridge Regression Model)

Regional volume	Standardized β coefficient (95% confidence intervals)	Raw *p* value	BH corrected *p* value
Gestation			
Amygdala left	0.08 (-0.01-0.18)	.04	.17
Amygdala right	0.03 (-0.07-0.12)	.52	.72
Anterior temporal lobe lateral part left gray matter	0.14 (0.06-0.22)	<.001	.01
Anterior temporal lobe lateral part left white matter	0.11 (0.01-0.21)	.01	.07
Anterior temporal lobe lateral part right gray matter	0.06 (-0.02-0.15)	.11	.28
Anterior temporal lobe lateral part right white matter	0.10 (-0.003-0.19)	.02	.11
Anterior temporal lobe medial part left gray matter	0.04 (-0.05-0.13)	.38	.62
Anterior temporal lobe medial part left white matter	0.03 (-0.07-0.14)	.36	.60
Anterior temporal lobe medial part right gray matter	0.05 (-0.04-0.14)	.25	.50
Anterior temporal lobe medial part right white matter	0.06 (-0.05-0.16)	.19	.42
Brainstem	-0.03 (-0.11-0.06)	.52	.72
Caudate nucleus left	0.06 (-0.24-0.36)	.18	.40
Caudate nucleus right	0.07 (-0.24-0.39)	.06	.21
Cerebellum left	-0.02 (-0.09-0.05)	.57	.75
Cerebellum right	-0.04 (-0.11-0.03)	.28	.53
Cerebrospinal fluid	-0.16 (-0.48-0.16)	.002	.02
Cingulate gyrus anterior part left gray matter	0.02 (-0.08-0.11)	.69	.83
Cingulate gyrus anterior part left white matter	0.07 (-0.03-0.17)	.06	.21
Cingulate gyrus anterior part right gray matter	-0.06 (-0.16-0.04)	.12	.30
Cingulate gyrus anterior part right white matter	0.04 (-0.06-0.14)	.32	.57
Cingulate gyrus posterior part left gray matter	-0.07 (-0.16-0.01)	.07	.22
Cingulate gyrus posterior part left white matter	0.06 (-0.03-0.14)	.14	.34
Cingulate gyrus posterior part right gray matter	-0.11 (-0.20- -0.02)	.01	.07
Cingulate gyrus posterior part right white matter	0.08 (-0.01-0.16)	.048	.18
Corpus callosum	0.10 (0.004-0.19)	.02	.09
Frontal lobe left gray matter	0.02 (-0.06-0.09)	.61	.78
Frontal lobe left white matter	0.14 (-0.15-0.42)	<.001	.01
Frontal lobe right gray matter	0.02 (-0.05-0.10)	.55	.74
Frontal lobe right white matter	0.17 (0.09-0.26)	<.001	.001
Gyri parahippocampalis et ambiens anterior part left gray matter	0.07 (-0.03-0.18)	.07	.22
Gyri parahippocampalis et ambiens anterior part left white matter	0.07 (-0.27-0.41)	.051	.18
Gyri parahippocampalis et ambiens anterior part right gray matter	0.07 (-0.03-0.17)	.10	.28
Gyri parahippocampalis et ambiens anterior part right white matter	0.02 (-0.31-0.35)	.67	.82
Gyri parahippocampalis et ambiens posterior part left gray matter	0.05 (-0.28-0.38)	.23	.47
Gyri parahippocampalis et ambiens posterior part left white matter	0.03 (-0.33-0.38)	.39	.62

© 2023 Mckinnon K et al. *JAMA Network Open.*

Regional volume	Standardized β coefficient (95% confidence intervals)	Raw *p* value	BH corrected *p* value
Gyri parahippocampalis et ambiens posterior part right gray matter	0.02 (-0.08-0.12)	.60	.77
Gyri parahippocampalis et ambiens posterior part right white matter	0.09 (-0.25-0.44)	.01	.07
Hippocampus left	-0.08 (-0.18-0.01)	.049	.18
Hippocampus right	-0.06 (-0.16-0.04)	.18	.39
Insula left gray matter	0.03 (-0.04-0.11)	.37	.60
Insula left white matter	0.09 (-0.21-0.39)	.03	.13
Insula right gray matter	-0.03 (-0.10-0.05)	.44	.64
Insula right white matter	0.17 (0.08-0.25)	<.001	.002
Lateral occipitotemporal gyrus gyrus fusiformis anterior part left gray matter	0.14 (-0.18-0.45)	.001	.02
Lateral occipitotemporal gyrus gyrus fusiformis anterior part left white matter	-0.08 (-0.17-0.02)	.08	.23
Lateral occipitotemporal gyrus gyrus fusiformis anterior part right gray matter	0.07 (-0.22-0.37)	.12	.30
Lateral occipitotemporal gyrus gyrus fusiformis anterior part right white matter	-0.02 (-0.12-0.07)	.58	.76
Lateral occipitotemporal gyrus gyrus fusiformis posterior part left gray matter	0.12 (0.02-0.21)	.01	.049
Lateral occipitotemporal gyrus gyrus fusiformis posterior part left white matter	-0.04 (-0.13-0.06)	.35	.60
Lateral occipitotemporal gyrus gyrus fusiformis posterior part right gray matter	0.08 (-0.01-0.17)	.052	.18
Lateral occipitotemporal gyrus gyrus fusiformis posterior part right white matter	0.002 (-0.10-0.10)	.96	.96
Lateral ventricle left	-0.08 (-0.44-0.27)	<.001	.01
Lateral ventricle right	-0.07 (-0.41-0.27)	<.001	.01
Lentiform nucleus left	-0.07 (-0.16-0.01)	.06	.21
Lentiform nucleus right	0.03 (-0.24-0.30)	.63	.80
Medial and inferior temporal gyri anterior part left gray matter	0.07 (-0.17-0.31)	.22	.45
Medial and inferior temporal gyri anterior part left white matter	0.04 (-0.05-0.13)	.34	.59
Medial and inferior temporal gyri anterior part right gray matter	0.09 (-0.13-0.31)	.11	.28
Medial and inferior temporal gyri anterior part right white matter	0.01 (-0.09-0.10)	.86	.91
Medial and inferior temporal gyri posterior part left gray matter	0.12 (0.05-0.18)	<.001	.01
Medial and inferior temporal gyri posterior part left white matter	-0.06 (-0.16-0.03)	.14	.33
Medial and inferior temporal gyri posterior part right gray matter	0.03 (-0.20-0.25)	.65	.82
Medial and inferior temporal gyri posterior part right white matter	-0.04 (-0.13-0.06)	.38	.62
Occipital lobe left gray matter	-0.04 (-0.10-0.03)	.28	.53
Occipital lobe left white matter	-0.08 (-0.18-0.02)	.06	.21
Occipital lobe right gray matter	-0.01 (-0.08-0.06)	.83	.90
Occipital lobe right white matter	-0.06 (-0.16-0.03)	.14	.34
Parietal lobe left gray matter	0.06 (0.01-0.12)	.03	.13
Parietal lobe left white matter	0.11 (0.02-0.20)	.01	.06
Parietal lobe right gray matter	0.08 (0.03-0.14)	.003	.03

© 2023 Mckinnon K et al. *JAMA Network Open.*

Regional volume	Standardized β coefficient (95% confidence intervals)	Raw *p* value	BH corrected *p* value
Parietal lobe right white matter	0.07 (-0.02-0.16)	.10	.28
Subthalamic nucleus left	-0.07 (-0.31-0.17)	.09	.27
Subthalamic nucleus right	-0.09 (-0.19-0.01)	.01	.08
Superior temporal gyrus middle part left gray matter	0.08 (-0.004-0.16)	.048	.18
Superior temporal gyrus middle part left white matter	0.05 (-0.05-0.14)	.24	.48
Superior temporal gyrus middle part right gray matter	0.10 (0.03-0.17)	.003	.03
Superior temporal gyrus middle part right white matter	0.05 (-0.05-0.14)	.25	.50
Superior temporal gyrus posterior part left gray matter	0.07 (-0.01-0.15)	.09	.26
Superior temporal gyrus posterior part left white matter	0.03 (-0.07-0.13)	.36	.60
Superior temporal gyrus posterior part right gray matter	0.04 (-0.04-0.12)	.27	.51
Superior temporal gyrus posterior part right white matter	0.04 (-0.06-0.14)	.34	.59
Thalamus left high intensity part in T2	-0.01 (-0.09-0.07)	.80	.89
Thalamus left low intensity part in T2	0.02 (-0.10-0.13)	.27	.51
Thalamus right high intensity part in T2	-0.05 (-0.13-0.03)	.20	.42
Thalamus right low intensity part in T2	0.01 (-0.08-0.11)	.76	.88
Interaction			
Caudate nucleus left	0.01 (-0.02-0.05)	.001	.02
Caudate nucleus right	0.01 (-0.03-0.05)	.002	.03
Cerebrospinal fluid	-0.03 (-0.07-0.01)	<.001	<.001
Frontal lobe left white matter	0.01 (-0.03-0.05)	.02	.09
Gyri parahippocampalis et ambiens anterior part left white matter	0.01 (-0.03-0.06)	<.001	.003
Gyri parahippocampalis et ambiens anterior part right white matter	0.01 (-0.03-0.05)	.01	.07
Gyri parahippocampalis et ambiens posterior part left gray matter	0.01 (-0.03-0.05)	.02	.09
Gyri parahippocampalis et ambiens posterior part left white matter	0.01 (-0.04-0.05)	.02	.09
Gyri parahippocampalis et ambiens posterior part right white matter	0.01 (-0.04-0.05)	.03	.13
Insula left white matter	0.01 (-0.03-0.05)	.003	.03
Lateral occipitotemporal gyrus gyrus fusiformis anterior part left gray matter	0.01 (-0.03-0.05)	.003	.03
Lateral occipitotemporal gyrus gyrus fusiformis anterior part right gray matter	0.01 (-0.02-0.05)	.01	.06
Lateral ventricle left	-0.01 (-0.05-0.04)	.001	.02
Lateral ventricle right	-0.01 (-0.05-0.04)	<.001	.01
Lentiform nucleus right	-0.02 (-0.05-0.02)	.02	.09
Medial and inferior temporal gyri anterior part left gray matter	0.02 (-0.01-0.05)	.002	.02
Medial and inferior temporal gyri anterior part right gray matter	0.02 (-0.01-0.04)	.01	.09
Medial and inferior temporal gyri posterior part right gray matter	0.01 (-0.01-0.04)	.049	.18
Subthalamic nucleus left	-0.01 (-0.04-0.02)	.004	.04
Maternal occupation			
Amygdala left	0.02 (-0.04-0.08)	.46	.67
Amygdala right	-0.02 (-0.08-0.04)	.42	.64

© 2023 Mckinnon K et al. *JAMA Network Open.*

Regional volume	Standardized β coefficient (95% confidence intervals)	Raw *p* value	BH corrected *p* value
Anterior temporal lobe lateral part left gray matter	0.02 (-0.03-0.07)	.44	.64
Anterior temporal lobe lateral part left white matter	0.03 (-0.03-0.10)	.25	.50
Anterior temporal lobe lateral part right gray matter	0.04 (-0.01-0.10)	.10	.28
Anterior temporal lobe lateral part right white matter	-0.01 (-0.07-0.05)	.73	.86
Anterior temporal lobe medial part left gray matter	0.05 (-0.01-0.11)	.09	.27
Anterior temporal lobe medial part left white matter	0.01 (-0.06-0.07)	.82	.90
Anterior temporal lobe medial part right gray matter	0.03 (-0.03-0.09)	.35	.59
Anterior temporal lobe medial part right white matter	0.01 (-0.06-0.08)	.74	.87
Brainstem	0.02 (-0.04-0.07)	.51	.72
Caudate nucleus left	-0.01 (-0.14-0.12)	.75	.87
Caudate nucleus right	-0.04 (-0.17-0.10)	.17	.39
Cerebellum left	0.04 (-0.01-0.08)	.12	.30
Cerebellum right	0.02 (-0.03-0.06)	.41	.64
Cerebrospinal fluid	0.08 (-0.07-0.22)	.02	.09
Cingulate gyrus anterior part left gray matter	0.01 (-0.05-0.07)	.64	.81
Cingulate gyrus anterior part left white matter	0.01 (-0.05-0.08)	.55	.74
Cingulate gyrus anterior part right gray matter	0.02 (-0.04-0.08)	.39	.62
Cingulate gyrus anterior part right white matter	0.02 (-0.04-0.09)	.32	.57
Cingulate gyrus posterior part left gray matter	0.01 (-0.05-0.06)	.78	.88
Cingulate gyrus posterior part left white matter	0.01 (-0.05-0.06)	.73	.86
Cingulate gyrus posterior part right gray matter	0.01 (-0.05-0.07)	.83	.90
Cingulate gyrus posterior part right white matter	0.02 (-0.04-0.08)	.41	.63
Corpus callosum	-0.01 (-0.07-0.05)	.77	.88
Frontal lobe left gray matter	0.01 (-0.03-0.06)	.54	.74
Frontal lobe left white matter	-0.004 (-0.13-0.12)	.88	.92
Frontal lobe right gray matter	0.01 (-0.04-0.06)	.77	.88
Frontal lobe right white matter	0.01 (-0.04-0.07)	.57	.75
Gyri parahippocampalis et ambiens anterior part left gray matter	0.003 (-0.06-0.07)	.90	.93
Gyri parahippocampalis et ambiens anterior part left white matter	-0.003 (-0.15-0.15)	.92	.94
Gyri parahippocampalis et ambiens anterior part right gray matter	0.02 (-0.04-0.08)	.50	.71
Gyri parahippocampalis et ambiens anterior part right white matter	0.001 (-0.14-0.15)	.98	.98
Gyri parahippocampalis et ambiens posterior part left gray matter	0.01 (-0.13-0.16)	.60	.77
Gyri parahippocampalis et ambiens posterior part left white matter	0.04 (-0.11-0.20)	.049	.18
Gyri parahippocampalis et ambiens posterior part right gray matter	0.04 (-0.03-0.10)	.18	.39
Gyri parahippocampalis et ambiens posterior part right white matter	-0.01 (-0.16-0.14)	.67	.82
Hippocampus left	0.04 (-0.02-0.10)	.14	.33
Hippocampus right	0.002 (-0.06-0.07)	.94	.95
Insula left gray matter	-0.02 (-0.07-0.03)	.50	.71

© 2023 Mckinnon K et al. *JAMA Network Open.*

Regional volume	Standardized β coefficient (95% confidence intervals)	Raw *p* value	BH corrected *p* value
Insula left white matter	-0.03 (-0.16-0.11)	.33	.59
Insula right gray matter	0.01 (-0.04-0.05)	.83	.90
Insula right white matter	0.01 (-0.05-0.06)	.84	.90
Lateral occipitotemporal gyrus gyrus fusiformis anterior part left gray matter	-0.01 (-0.15-0.13)	.67	.82
Lateral occipitotemporal gyrus gyrus fusiformis anterior part left white matter	0.04 (-0.03-0.10)	.19	.42
Lateral occipitotemporal gyrus gyrus fusiformis anterior part right gray matter	-0.02 (-0.15-0.11)	.49	.70
Lateral occipitotemporal gyrus gyrus fusiformis anterior part right white matter	-0.01 (-0.08-0.05)	.67	.82
Lateral occipitotemporal gyrus gyrus fusiformis posterior part left gray matter	-0.01 (-0.07-0.05)	.77	.88
Lateral occipitotemporal gyrus gyrus fusiformis posterior part left white matter	-0.02 (-0.08-0.04)	.43	.64
Lateral occipitotemporal gyrus gyrus fusiformis posterior part right gray matter	0.06 (-0.004-0.12)	.04	.16
Lateral occipitotemporal gyrus gyrus fusiformis posterior part right white matter	-0.01 (-0.07-0.06)	.81	.89
Lateral ventricle left	0.04 (-0.12-0.19)	.04	.16
Lateral ventricle right	0.02 (-0.13-0.17)	.14	.33
Lentiform nucleus left	0.01 (-0.05-0.06)	.80	.89
Lentiform nucleus right	0.07 (-0.05-0.19)	.03	.14
Medial and inferior temporal gyri anterior part left gray matter	-0.01 (-0.12-0.09)	.66	.82
Medial and inferior temporal gyri anterior part left white matter	0.05 (-0.01-0.11)	.07	.21
Medial and inferior temporal gyri anterior part right gray matter	0.01 (-0.09-0.10)	.83	.90
Medial and inferior temporal gyri anterior part right white matter	0.03 (-0.03-0.09)	.29	.55
Medial and inferior temporal gyri posterior part left gray matter	0.002 (-0.04-0.05)	.92	.94
Medial and inferior temporal gyri posterior part left white matter	-0.004 (-0.07-0.06)	.90	.93
Medial and inferior temporal gyri posterior part right gray matter	-0.02 (-0.12-0.08)	.58	.76
Medial and inferior temporal gyri posterior part right white matter	0.004 (-0.06-0.07)	.89	.93
Occipital lobe left gray matter	0.05 (0.01-0.09)	.02	.10
Occipital lobe left white matter	0.02 (-0.04-0.09)	.41	.63
Occipital lobe right gray matter	0.06 (0.02-0.11)	.01	.049
Occipital lobe right white matter	0.02 (-0.04-0.09)	.43	.64
Parietal lobe left gray matter	0.03 (-0.01-0.07)	.11	.28
Parietal lobe left white matter	0.004 (-0.05-0.06)	.88	.92
Parietal lobe right gray matter	0.04 (0.001-0.08)	.04	.17
Parietal lobe right white matter	0.01 (-0.05-0.07)	.79	.89
Subthalamic nucleus left	0.07 (-0.02-0.17)	.01	.07
Subthalamic nucleus right	0.02 (-0.05-0.08)	.51	.72
Superior temporal gyrus middle part left gray matter	0.02 (-0.03-0.07)	.43	.64
Superior temporal gyrus middle part left white matter	0.03 (-0.03-0.10)	.22	.45
Superior temporal gyrus middle part right gray matter	0.04 (-0.01-0.08)	.10	.27

© 2023 Mckinnon K et al. *JAMA Network Open.*

Regional volume	Standardized β coefficient (95% confidence intervals)	Raw *p* value	BH corrected *p* value
Superior temporal gyrus middle part right white matter	0.01 (-0.05-0.07)	.70	.84
Superior temporal gyrus posterior part left gray matter	0.03 (-0.02-0.09)	.17	.39
Superior temporal gyrus posterior part left white matter	0.02 (-0.04-0.09)	.36	.60
Superior temporal gyrus posterior part right gray matter	0.03 (-0.03-0.08)	.31	.57
Superior temporal gyrus posterior part right white matter	-0.003 (-0.07-0.06)	.92	.94
Thalamus left high intensity part in T2	0.02 (-0.03-0.08)	.32	.57
Thalamus left low intensity part in T2	0.01 (-0.07-0.08)	.56	.75
Thalamus right high intensity part in T2	0.03 (-0.02-0.08)	.23	.46
Thalamus right low intensity part in T2	0.02 (-0.04-0.09)	.37	.60

Fully adjusted ridge regression model, including gestation at birth, maternal occupation, gestation at MRI, the interaction term (where significant), birth weight z-score, birth head circumference z-score, sex, smoking in pregnancy, and breast milk at discharge. Corrected for false discovery rate.
BH = Benjamini-Hochberg correction.

© 2023 Mckinnon K et al. *JAMA Network Open.*

eTable 9. Regional Brain Volumes: Association With Gestational Age, Paternal Occupation, and Interaction Effect (Fully Adjusted Ridge Regression Model)

**Regional volume**	**Standardized β coefficient (95% confidence intervals)**	**Raw *p* value**	**BH corrected *p* value**
Gestation			
Amygdala left	0.09 (-0.01-0.18)	.04	.17
Amygdala right	0.02 (-0.07-0.12)	.59	.76
Anterior temporal lobe lateral part left gray matter	0.14 (0.05-0.22)	<.001	.01
Anterior temporal lobe lateral part left white matter	0.11 (0.01-0.21)	.01	.08
Anterior temporal lobe lateral part right gray matter	0.06 (-0.02-0.15)	.12	.31
Anterior temporal lobe lateral part right white matter	0.09 (-0.01-0.19)	.04	.18
Anterior temporal lobe medial part left gray matter	0.03 (-0.06-0.12)	.44	.65
Anterior temporal lobe medial part left white matter	0.03 (-0.08-0.14)	.41	.64
Anterior temporal lobe medial part right gray matter	0.05 (-0.05-0.14)	.27	.50
Anterior temporal lobe medial part right white matter	0.05 (-0.05-0.16)	.22	.45
Brainstem	-0.02 (-0.11-0.07)	.60	.76
Caudate nucleus left	0.18 (0.09-0.26)	<.001	<.001
Caudate nucleus right	0.10 (-0.28-0.48)	.01	.04
Cerebellum left	-0.01 (-0.08-0.07)	.88	.94
Cerebellum right	-0.02 (-0.09-0.04)	.48	.69
Cerebrospinal fluid	-0.20 (-0.57-0.17)	<.001	<.001
Cingulate gyrus anterior part left gray matter	0.02 (-0.07-0.11)	.60	.76
Cingulate gyrus anterior part left white matter	0.03 (-0.37-0.43)	.27	.49
Cingulate gyrus anterior part right gray matter	-0.06 (-0.16-0.03)	.14	.35
Cingulate gyrus anterior part right white matter	0.04 (-0.07-0.14)	.34	.57
Cingulate gyrus posterior part left gray matter	-0.07 (-0.16-0.01)	.08	.23
Cingulate gyrus posterior part left white matter	0.06 (-0.03-0.14)	.16	.38
Cingulate gyrus posterior part right gray matter	-0.12 (-0.21- -0.02)	.01	.051
Cingulate gyrus posterior part right white matter	0.08 (-0.01-0.17)	.049	.18
Corpus callosum	0.09 (-0.01-0.18)	.03	.17
Frontal lobe left gray matter	0.02 (-0.05-0.10)	.56	.75
Frontal lobe left white matter	0.21 (0.12-0.29)	<.001	<.001
Frontal lobe right gray matter	0.03 (-0.05-0.10)	.47	.69
Frontal lobe right white matter	0.17 (0.09-0.25)	<.001	<.001
Gyri parahippocampalis et ambiens anterior part left gray matter	0.08 (-0.02-0.19)	.049	.18
Gyri parahippocampalis et ambiens anterior part left white matter	0.07 (-0.34-0.48)	.06	.20
Gyri parahippocampalis et ambiens anterior part right gray matter	0.08 (-0.02-0.18)	.053	.19
Gyri parahippocampalis et ambiens anterior part right white matter	0.02 (-0.38-0.43)	.53	.73
Gyri parahippocampalis et ambiens posterior part left gray matter	0.12 (0.02-0.22)	.004	.03
Gyri parahippocampalis et ambiens posterior part left white matter	0.06 (-0.05-0.17)	.12	.31
Gyri parahippocampalis et ambiens posterior part right gray matter	0.04 (-0.06-0.14)	.37	.59

© 2023 Mckinnon K et al. *JAMA Network Open.*

**Regional volume**	**Standardized β coefficient (95% confidence intervals)**	**Raw *p* value**	**BH corrected *p* value**
Gyri parahippocampalis et ambiens posterior part right white matter	0.14 (0.04-0.24)	<.001	.01
Hippocampus left	-0.06 (-0.16-0.03)	.12	.31
Hippocampus right	-0.05 (-0.16-0.05)	.21	.45
Insula left gray matter	0.03 (-0.04-0.11)	.36	.58
Insula left white matter	0.11 (-0.25-0.47)	.004	.03
Insula right gray matter	-0.03 (-0.10-0.05)	.49	.70
Insula right white matter	0.11 (-0.25-0.47)	.003	.03
Lateral occipitotemporal gyrus gyrus fusiformis anterior part left gray matter	0.22 (0.13-0.31)	<.001	<.001
Lateral occipitotemporal gyrus gyrus fusiformis anterior part left white matter	-0.08 (-0.18-0.02)	.06	.20
Lateral occipitotemporal gyrus gyrus fusiformis anterior part right gray matter	0.17 (0.08-0.25)	<.001	<.001
Lateral occipitotemporal gyrus gyrus fusiformis anterior part right white matter	-0.02 (-0.12-0.08)	.57	.76
Lateral occipitotemporal gyrus gyrus fusiformis posterior part left gray matter	0.12 (0.02-0.21)	.01	.04
Lateral occipitotemporal gyrus gyrus fusiformis posterior part left white matter	-0.03 (-0.13-0.06)	.41	.64
Lateral occipitotemporal gyrus gyrus fusiformis posterior part right gray matter	0.08 (-0.01-0.18)	.04	.18
Lateral occipitotemporal gyrus gyrus fusiformis posterior part right white matter	0.01 (-0.09-0.11)	.73	.87
Lateral ventricle left	-0.08 (-0.49-0.33)	.001	.01
Lateral ventricle right	-0.07 (-0.47-0.33)	.001	.01
Lentiform nucleus left	-0.08 (-0.16-0.01)	.06	.20
Lentiform nucleus right	0.01 (-0.31-0.33)	.90	.95
Medial and inferior temporal gyri anterior part left gray matter	0.22 (0.15-0.29)	<.001	<.001
Medial and inferior temporal gyri anterior part left white matter	0.05 (-0.04-0.14)	.24	.46
Medial and inferior temporal gyri anterior part right gray matter	0.12 (-0.15-0.39)	.04	.18
Medial and inferior temporal gyri anterior part right white matter	0.02 (-0.08-0.12)	.59	.76
Medial and inferior temporal gyri posterior part left gray matter	0.11 (0.04-0.17)	.001	.01
Medial and inferior temporal gyri posterior part left white matter	-0.05 (-0.15-0.05)	.22	.45
Medial and inferior temporal gyri posterior part right gray matter	0.13 (0.06-0.19)	<.001	.002
Medial and inferior temporal gyri posterior part right white matter	-0.02 (-0.12-0.08)	.54	.73
Occipital lobe left gray matter	0.10 (-0.17-0.38)	.13	.32
Occipital lobe left white matter	-0.07 (-0.17-0.03)	.12	.31
Occipital lobe right gray matter	0.14 (-0.16-0.44)	.03	.16
Occipital lobe right white matter	-0.05 (-0.15-0.05)	.23	.45
Parietal lobe left gray matter	0.06 (0.002-0.12)	.04	.18
Parietal lobe left white matter	0.12 (0.03-0.21)	.004	.03
Parietal lobe right gray matter	0.09 (0.03-0.14)	.003	.03
Parietal lobe right white matter	0.08 (-0.01-0.17)	.06	.20
Subthalamic nucleus left	-0.08 (-0.25-0.08)	.03	.16

© 2023 Mckinnon K et al. *JAMA Network Open.*

Regional volume	Standardized β coefficient (95% confidence intervals)	Raw *p* value	BH corrected *p* value
Subthalamic nucleus right	-0.05 (-0.36-0.25)	.21	.45
Superior temporal gyrus middle part left gray matter	0.07 (-0.01-0.15)	.07	.21
Superior temporal gyrus middle part left white matter	0.06 (-0.03-0.16)	.12	.31
Superior temporal gyrus middle part right gray matter	0.10 (0.03-0.18)	.003	.03
Superior temporal gyrus middle part right white matter	0.06 (-0.03-0.16)	.12	.31
Superior temporal gyrus posterior part left gray matter	0.07 (-0.01-0.15)	.08	.24
Superior temporal gyrus posterior part left white matter	0.04 (-0.06-0.14)	.34	.57
Superior temporal gyrus posterior part right gray matter	0.04 (-0.04-0.12)	.33	.55
Superior temporal gyrus posterior part right white matter	0.04 (-0.06-0.13)	.38	.60
Thalamus left high intensity part in T2	-0.01 (-0.09-0.07)	.85	.92
Thalamus left low intensity part in T2	0.03 (-0.09-0.14)	.18	.40
Thalamus right high intensity part in T2	-0.05 (-0.13-0.03)	.21	.45
Thalamus right low intensity part in T2	0.02 (-0.08-0.11)	.71	.86
Interaction			
Caudate nucleus right	0.01 (-0.04-0.05)	.04	.17
Cerebrospinal fluid	-0.02 (-0.06-0.03)	<.001	<.001
Cingulate gyrus anterior part left white matter	0.01 (-0.04-0.06)	.04	.18
Gyri parahippocampalis et ambiens anterior part left white matter	0.01 (-0.04-0.07)	<.001	<.001
Gyri parahippocampalis et ambiens anterior part right white matter	0.01 (-0.04-0.06)	.01	.08
Insula left white matter	0.01 (-0.04-0.06)	.01	.08
Insula right white matter	0.01 (-0.04-0.05)	.04	.18
Lateral ventricle left	-0.01 (-0.06-0.05)	<.001	.01
Lateral ventricle right	-0.01 (-0.06-0.04)	<.001	.01
Lentiform nucleus right	-0.01 (-0.06-0.03)	.045	.18
Medial and inferior temporal gyri anterior part right gray matter	0.01 (-0.02-0.05)	.049	.18
Occipital lobe left gray matter	-0.02 (-0.05-0.02)	.04	.18
Occipital lobe right gray matter	-0.02 (-0.06-0.02)	.02	.12
Subthalamic nucleus left	-0.01 (-0.03-0.01)	.004	.03
Subthalamic nucleus right	-0.01 (-0.05-0.03)	.03	.16
Paternal education			
Amygdala left	0.01 (-0.06-0.08)	.78	.91
Amygdala right	-0.01 (-0.07-0.06)	.87	.94
Anterior temporal lobe lateral part left gray matter	-0.02 (-0.08-0.04)	.52	.73
Anterior temporal lobe lateral part left white matter	0.04 (-0.03-0.11)	.24	.46
Anterior temporal lobe lateral part right gray matter	0.02 (-0.04-0.08)	.57	.76
Anterior temporal lobe lateral part right white matter	0.05 (-0.03-0.12)	.15	.35
Anterior temporal lobe medial part left gray matter	0.005 (-0.06-0.07)	.88	.94
Anterior temporal lobe medial part left white matter	0.02 (-0.06-0.09)	.59	.76
Anterior temporal lobe medial part right gray matter	0.04 (-0.02-0.11)	.17	.39

© 2023 Mckinnon K et al. *JAMA Network Open.*

Regional volume	Standardized β coefficient (95% confidence intervals)	Raw *p* value	BH corrected *p* value
Anterior temporal lobe medial part right white matter	0.07 (-0.01-0.14)	.04	.18
Brainstem	-0.02 (-0.08-0.04)	.52	.73
Caudate nucleus left	-0.01 (-0.07-0.05)	.67	.83
Caudate nucleus right	-0.03 (-0.22-0.17)	.36	.58
Cerebellum left	0.001 (-0.05-0.05)	.97	.99
Cerebellum right	-0.01 (-0.06-0.04)	.78	.91
Cerebrospinal fluid	0.04 (-0.15-0.23)	.09	.25
Cingulate gyrus anterior part left gray matter	0.02 (-0.05-0.09)	.49	.70
Cingulate gyrus anterior part left white matter	0.03 (-0.17-0.24)	.20	.43
Cingulate gyrus anterior part right gray matter	0.04 (-0.03-0.10)	.23	.45
Cingulate gyrus anterior part right white matter	0.06 (-0.01-0.13)	.046	.18
Cingulate gyrus posterior part left gray matter	-0.004 (-0.07-0.06)	.91	.95
Cingulate gyrus posterior part left white matter	0.03 (-0.03-0.09)	.31	.53
Cingulate gyrus posterior part right gray matter	0.01 (-0.05-0.08)	.70	.86
Cingulate gyrus posterior part right white matter	0.03 (-0.03-0.10)	.27	.50
Corpus callosum	0.04 (-0.03-0.10)	.22	.45
Frontal lobe left gray matter	0.01 (-0.05-0.06)	.83	.92
Frontal lobe left white matter	0.03 (-0.03-0.09)	.26	.49
Frontal lobe right gray matter	-0.01 (-0.06-0.05)	.83	.92
Frontal lobe right white matter	0.05 (-0.01-0.11)	.08	.23
Gyri parahippocampalis et ambiens anterior part left gray matter	-0.03 (-0.11-0.04)	.30	.51
Gyri parahippocampalis et ambiens anterior part left white matter	-0.06 (-0.27-0.15)	.048	.18
Gyri parahippocampalis et ambiens anterior part right gray matter	-0.05 (-0.12-0.02)	.10	.28
Gyri parahippocampalis et ambiens anterior part right white matter	-0.01 (-0.21-0.20)	.78	.91
Gyri parahippocampalis et ambiens posterior part left gray matter	-0.001 (-0.07-0.07)	.98	.99
Gyri parahippocampalis et ambiens posterior part left white matter	0.02 (-0.06-0.09)	.53	.73
Gyri parahippocampalis et ambiens posterior part right gray matter	-0.001 (-0.07-0.07)	.96	.99
Gyri parahippocampalis et ambiens posterior part right white matter	-0.01 (-0.08-0.06)	.82	.92
Hippocampus left	-0.01 (-0.07-0.06)	.85	.92
Hippocampus right	-0.003 (-0.08-0.07)	.93	.97
Insula left gray matter	-0.05 (-0.10-0.01)	.08	.23
Insula left white matter	0.02 (-0.17-0.20)	.56	.75
Insula right gray matter	-0.03 (-0.08-0.03)	.28	.50
Insula right white matter	0.02 (-0.17-0.20)	.58	.76
Lateral occipitotemporal gyrus gyrus fusiformis anterior part left gray matter	0.01 (-0.06-0.07)	.80	.91
Lateral occipitotemporal gyrus gyrus fusiformis anterior part left white matter	0.04 (-0.03-0.11)	.21	.45
Lateral occipitotemporal gyrus gyrus fusiformis anterior part right gray matter	-0.001 (-0.06-0.06)	.97	.99

© 2023 Mckinnon K et al. *JAMA Network Open.*

**Regional volume**	**Standardized β coefficient (95% confidence intervals)**	**Raw *p* value**	**BH corrected *p* value**
Lateral occipitotemporal gyrus gyrus fusiformis anterior part right white matter	-0.002 (-0.07-0.07)	.95	.99
Lateral occipitotemporal gyrus gyrus fusiformis posterior part left gray matter	-0.03 (-0.10-0.04)	.35	.57
Lateral occipitotemporal gyrus gyrus fusiformis posterior part left white matter	0.02 (-0.04-0.09)	.43	.65
Lateral occipitotemporal gyrus gyrus fusiformis posterior part right gray matter	-0.01 (-0.07-0.06)	.80	.91
Lateral occipitotemporal gyrus gyrus fusiformis posterior part right white matter	-0.02 (-0.09-0.05)	.49	.70
Lateral ventricle left	0.04 (-0.17-0.25)	.06	.20
Lateral ventricle right	0.02 (-0.18-0.22)	.27	.49
Lentiform nucleus left	-0.04 (-0.10-0.02)	.17	.39
Lentiform nucleus right	0.04 (-0.12-0.20)	.29	.51
Medial and inferior temporal gyri anterior part left gray matter	0.03 (-0.02-0.08)	.27	.49
Medial and inferior temporal gyri anterior part left white matter	0.04 (-0.02-0.11)	.15	.35
Medial and inferior temporal gyri anterior part right gray matter	-0.01 (-0.15-0.13)	.78	.91
Medial and inferior temporal gyri anterior part right white matter	0.03 (-0.04-0.10)	.40	.62
Medial and inferior temporal gyri posterior part left gray matter	0.01 (-0.04-0.05)	.81	.92
Medial and inferior temporal gyri posterior part left white matter	-0.02 (-0.09-0.05)	.54	.73
Medial and inferior temporal gyri posterior part right gray matter	0.01 (-0.04-0.05)	.79	.91
Medial and inferior temporal gyri posterior part right white matter	-0.001 (-0.07-0.07)	.96	.99
Occipital lobe left gray matter	0.07 (-0.08-0.21)	.09	.24
Occipital lobe left white matter	0.01 (-0.06-0.08)	.67	.83
Occipital lobe right gray matter	0.07 (-0.09-0.22)	.07	.21
Occipital lobe right white matter	-0.01 (-0.08-0.06)	.69	.84
Parietal lobe left gray matter	0.02 (-0.02-0.06)	.44	.65
Parietal lobe left white matter	0.04 (-0.03-0.10)	.22	.45
Parietal lobe right gray matter	0.01 (-0.03-0.05)	.72	.86
Parietal lobe right white matter	0.03 (-0.03-0.10)	.30	.51
Subthalamic nucleus left	0.0002 (-0.06-0.06)	>,99	>.99
Subthalamic nucleus right	-0.02 (-0.18-0.13)	.44	.65
Superior temporal gyrus middle part left gray matter	-0.001 (-0.06-0.06)	.98	.99
Superior temporal gyrus middle part left white matter	0.03 (-0.04-0.10)	.28	.50
Superior temporal gyrus middle part right gray matter	0.01 (-0.05-0.06)	.83	.92
Superior temporal gyrus middle part right white matter	0.04 (-0.03-0.11)	.18	.41
Superior temporal gyrus posterior part left gray matter	-0.004 (-0.06-0.05)	.88	.94
Superior temporal gyrus posterior part left white matter	0.05 (-0.02-0.12)	.07	.21
Superior temporal gyrus posterior part right gray matter	0.01 (-0.05-0.07)	.66	.83
Superior temporal gyrus posterior part right white matter	0.03 (-0.04-0.10)	.34	.57
Thalamus left high intensity part in T2	-0.01 (-0.07-0.04)	.59	.76
Thalamus left low intensity part in T2	-0.003 (-0.08-0.08)	.85	.92

© 2023 Mckinnon K et al. *JAMA Network Open.*

Regional volume	Standardized β coefficient (95% confidence intervals)	Raw *p* value	BH corrected *p* value
Thalamus right high intensity part in T2	-0.02 (-0.08-0.03)	.43	.65
Thalamus right low intensity part in T2	-0.07 (-0.14- -0.01)	.02	.12

Fully adjusted ridge regression model, including gestation at birth, paternal occupation, gestation at MRI, the interaction term (where significant), birth weight z-score, birth head circumference z-score, sex, smoking in pregnancy, and breast milk at discharge. Corrected for false discovery rate.
BH = Benjamini-Hochberg correction.

eTable 10. Regional Brain Volumes: Association With Gestational Age, Subjective Socioeconomic Status, and Interaction Effect (Fully Adjusted Ridge Regression Model)

Regional volume	Standardized β coefficient (95% confidence intervals)	Raw *p* value	BH corrected *p* value
Gestation			
Amygdala left	0.06 (-0.05-0.16)	.21	.46
Amygdala right	0.002 (-0.10-0.10)	.96	.97
Anterior temporal lobe lateral part left gray matter	0.14 (0.06-0.23)	<.001	.01
Anterior temporal lobe lateral part left white matter	0.09 (-0.02-0.19)	.054	.18
Anterior temporal lobe lateral part right gray matter	0.06 (-0.03-0.15)	.19	.45
Anterior temporal lobe lateral part right white matter	0.09 (-0.02-0.19)	.04	.17
Anterior temporal lobe medial part left gray matter	0.06 (-0.04-0.15)	.21	.46
Anterior temporal lobe medial part left white matter	0.02 (-0.10-0.13)	.67	.81
Anterior temporal lobe medial part right gray matter	0.04 (-0.06-0.14)	.40	.67
Anterior temporal lobe medial part right white matter	0.05 (-0.06-0.16)	.27	.55
Brainstem	-0.03 (-0.12-0.06)	.51	.75
Caudate nucleus left	0.16 (0.07-0.26)	<.001	.003
Caudate nucleus right	0.08 (-0.37-0.53)	.02	.10
Cerebellum left	-0.01 (-0.08-0.07)	.88	.95
Cerebellum right	-0.03 (-0.10-0.05)	.48	.72
Cerebrospinal fluid	-0.19 (-0.61-0.23)	<.001	.003
Cingulate gyrus anterior part left gray matter	-0.01 (-0.11-0.09)	.82	.91
Cingulate gyrus anterior part left white matter	0.06 (-0.05-0.17)	.12	.33
Cingulate gyrus anterior part right gray matter	-0.07 (-0.17-0.04)	.13	.36
Cingulate gyrus anterior part right white matter	0.03 (-0.08-0.14)	.42	.67
Cingulate gyrus posterior part left gray matter	-0.06 (-0.15-0.03)	.18	.43
Cingulate gyrus posterior part left white matter	0.05 (-0.04-0.15)	.20	.46
Cingulate gyrus posterior part right gray matter	-0.10 (-0.19-0.002)	.03	.14
Cingulate gyrus posterior part right white matter	0.07 (-0.03-0.16)	.12	.33
Corpus callosum	0.09 (-0.01-0.19)	.03	.13
Frontal lobe left gray matter	0.02 (-0.06-0.10)	.55	.75
Frontal lobe left white matter	0.11 (-0.29-0.51)	.01	.049
Frontal lobe right gray matter	0.02 (-0.06-0.10)	.57	.77
Frontal lobe right white matter	0.09 (-0.33-0.50)	.02	.10
Gyri parahippocampalis et ambiens anterior part left gray matter	0.07 (-0.04-0.18)	.09	.28
Gyri parahippocampalis et ambiens anterior part left white matter	0.09 (-0.39-0.57)	.01	.049
Gyri parahippocampalis et ambiens anterior part right gray matter	0.06 (-0.05-0.16)	.21	.46
Gyri parahippocampalis et ambiens anterior part right white matter	0.09 (-0.01-0.19)	.049	.17
Gyri parahippocampalis et ambiens posterior part left gray matter	0.12 (0.02-0.22)	.01	.06
Gyri parahippocampalis et ambiens posterior part left white matter	0.06 (-0.05-0.17)	.16	.40

© 2023 Mckinnon K et al. *JAMA Network Open.*

**Regional volume**	**Standardized β coefficient (95% confidence intervals)**	**Raw *p* value**	**BH corrected *p* value**
Gyri parahippocampalis et ambiens posterior part right gray matter	-0.003 (-0.11-0.10)	.95	.97
Gyri parahippocampalis et ambiens posterior part right white matter	0.09 (-0.38-0.57)	.004	.046
Hippocampus left	-0.04 (-0.48-0.40)	.33	.59
Hippocampus right	-0.03 (-0.13-0.08)	.52	.75
Insula left gray matter	0.04 (-0.05-0.12)	.36	.62
Insula left white matter	0.18 (0.09-0.27)	<.001	<.001
Insula right gray matter	0.10 (-0.27-0.47)	.11	.33
Insula right white matter	0.10 (-0.34-0.54)	.01	.07
Lateral occipitotemporal gyrus gyrus fusiformis anterior part left gray matter	0.14 (-0.31-0.58)	<.001	.01
Lateral occipitotemporal gyrus gyrus fusiformis anterior part left white matter	-0.08 (-0.18-0.02)	.08	.27
Lateral occipitotemporal gyrus gyrus fusiformis anterior part right gray matter	0.15 (0.06-0.25)	<.001	.005
Lateral occipitotemporal gyrus gyrus fusiformis anterior part right white matter	-0.03 (-0.14-0.08)	.53	.75
Lateral occipitotemporal gyrus gyrus fusiformis posterior part left gray matter	0.09 (-0.004-0.19)	.03	.14
Lateral occipitotemporal gyrus gyrus fusiformis posterior part left white matter	0.002 (-0.44-0.44)	.96	.97
Lateral occipitotemporal gyrus gyrus fusiformis posterior part right gray matter	0.05 (-0.04-0.15)	.22	.48
Lateral occipitotemporal gyrus gyrus fusiformis posterior part right white matter	-0.01 (-0.12-0.10)	.80	.89
Lateral ventricle left	-0.09 (-0.59-0.42)	<.001	.005
Lateral ventricle right	-0.07 (-0.55-0.40)	<.001	.005
Lentiform nucleus left	-0.10 (-0.19- -0.01)	.02	.11
Lentiform nucleus right	0.06 (-0.32-0.44)	.38	.64
Medial and inferior temporal gyri anterior part left gray matter	0.24 (0.16-0.31)	<.001	<.001
Medial and inferior temporal gyri anterior part left white matter	0.04 (-0.06-0.13)	.41	.67
Medial and inferior temporal gyri anterior part right gray matter	0.09 (-0.24-0.41)	.17	.41
Medial and inferior temporal gyri anterior part right white matter	0.02 (-0.08-0.13)	.62	.79
Medial and inferior temporal gyri posterior part left gray matter	0.12 (0.04-0.19)	.001	.01
Medial and inferior temporal gyri posterior part left white matter	-0.07 (-0.18-0.03)	.11	.33
Medial and inferior temporal gyri posterior part right gray matter	0.13 (0.06-0.20)	<.001	.005
Medial and inferior temporal gyri posterior part right white matter	-0.03 (-0.13-0.08)	.54	.75
Occipital lobe left gray matter	-0.05 (-0.12-0.02)	.19	.45
Occipital lobe left white matter	-0.08 (-0.18-0.03)	.10	.32
Occipital lobe right gray matter	-0.02 (-0.10-0.05)	.52	.75
Occipital lobe right white matter	-0.07 (-0.18-0.03)	.11	.33
Parietal lobe left gray matter	0.06 (0.004-0.12)	.03	.14
Parietal lobe left white matter	0.11 (0.02-0.21)	.01	.06
Parietal lobe right gray matter	0.08 (0.02-0.15)	.01	.049

© 2023 Mckinnon K et al. *JAMA Network Open.*

Regional volume	Standardized β coefficient (95% confidence intervals)	Raw *p* value	BH corrected *p* value
Parietal lobe right white matter	0.07 (-0.03-0.17)	.10	.31
Subthalamic nucleus left	-0.18 (-0.28- -0.07)	<.001	.005
Subthalamic nucleus right	-0.12 (-0.22- -0.02)	.003	.03
Superior temporal gyrus middle part left gray matter	0.08 (-0.003-0.17)	.04	.17
Superior temporal gyrus middle part left white matter	0.06 (-0.05-0.16)	.16	.41
Superior temporal gyrus middle part right gray matter	0.10 (0.02-0.18)	.01	.06
Superior temporal gyrus middle part right white matter	0.06 (-0.04-0.17)	.12	.34
Superior temporal gyrus posterior part left gray matter	0.07 (-0.02-0.16)	.10	.31
Superior temporal gyrus posterior part left white matter	0.03 (-0.08-0.14)	.41	.67
Superior temporal gyrus posterior part right gray matter	0.04 (-0.04-0.13)	.28	.56
Superior temporal gyrus posterior part right white matter	0.05 (-0.05-0.16)	.21	.46
Thalamus left high intensity part in T2	-0.03 (-0.12-0.05)	.41	.67
Thalamus left low intensity part in T2	0.02 (-0.11-0.14)	.28	.56
Thalamus right high intensity part in T2	-0.06 (-0.15-0.03)	.17	.41
Thalamus right low intensity part in T2	0.01 (-0.10-0.12)	.77	.87
Interaction			
Caudate nucleus right	0.001 (-0.005-0.01)	.01	.06
Cerebrospinal fluid	-0.003 (-0.01-0.003)	<.001	<.001
Frontal lobe left white matter	0.001 (-0.004-0.01)	.01	.08
Frontal lobe right white matter	0.001 (-0.004-0.01)	.03	.13
Gyri parahippocampalis et ambiens anterior part left white matter	0.001 (-0.01-0.01)	.049	.17
Gyri parahippocampalis et ambiens posterior part right white matter	0.001 (-0.01-0.01)	.045	.17
Hippocampus left	-0.001 (-0.01-0.005)	.04	.15
Insula right gray matter	-0.002 (-0.01-0.002)	.01	.053
Insula right white matter	0.001 (-0.005-0.01)	.04	.15
Lateral occipitotemporal gyrus gyrus fusiformis anterior part left gray matter	0.001 (-0.004-0.01)	.01	.08
Lateral occipitotemporal gyrus gyrus fusiformis posterior part left white matter	-0.001 (-0.01-0.004)	.01	.06
Lateral ventricle left	-0.001 (-0.01-0.01)	.001	.02
Lateral ventricle right	-0.001 (-0.01-0.01)	<.001	.01
Lentiform nucleus right	-0.003 (-0.01-0.002)	.001	.02
Medial and inferior temporal gyri anterior part right gray matter	0.002 (-0.002-0.01)	.03	.13
Subjective socioeconomic status			
Amygdala left	-0.001 (-0.01-0.01)	.87	.94
Amygdala right	0.0003 (-0.01-0.01)	.93	.97
Anterior temporal lobe lateral part left gray matter	-0.01 (-0.01-0.002)	.13	.35
Anterior temporal lobe lateral part left white matter	0.002 (-0.01-0.01)	.65	.80
Anterior temporal lobe lateral part right gray matter	0.002 (-0.01-0.01)	.64	.79
Anterior temporal lobe lateral part right white matter	0.004 (-0.01-0.01)	.34	.60

© 2023 Mckinnon K et al. *JAMA Network Open.*

**Regional volume**	**Standardized β coefficient (95% confidence intervals)**	**Raw *p* value**	**BH corrected *p* value**
Anterior temporal lobe medial part left gray matter	-0.01 (-0.02- -0.002)	.01	.06
Anterior temporal lobe medial part left white matter	-0.001 (-0.01-0.01)	.79	.88
Anterior temporal lobe medial part right gray matter	-0.002 (-0.01-0.01)	.66	.80
Anterior temporal lobe medial part right white matter	-0.00005 (-0.01-0.01)	.99	.99
Brainstem	0.001 (-0.01-0.01)	.77	.87
Caudate nucleus left	0.003 (-0.01-0.01)	.47	.72
Caudate nucleus right	0.003 (-0.02-0.02)	.47	.72
Cerebellum left	0.003 (-0.004-0.01)	.35	.61
Cerebellum right	0.002 (-0.005-0.01)	.61	.78
Cerebrospinal fluid	0.01 (-0.01-0.03)	.02	.12
Cingulate gyrus anterior part left gray matter	-0.0004 (-0.01-0.01)	.92	.97
Cingulate gyrus anterior part left white matter	0.001 (-0.01-0.01)	.83	.91
Cingulate gyrus anterior part right gray matter	-0.002 (-0.01-0.01)	.63	.79
Cingulate gyrus anterior part right white matter	0.001 (-0.01-0.01)	.75	.86
Cingulate gyrus posterior part left gray matter	-0.002 (-0.01-0.01)	.67	.81
Cingulate gyrus posterior part left white matter	0.005 (-0.003-0.01)	.21	.46
Cingulate gyrus posterior part right gray matter	-0.0003 (-0.01-0.01)	.93	.97
Cingulate gyrus posterior part right white matter	-0.001 (-0.01-0.01)	.71	.84
Corpus callosum	0.002 (-0.01-0.01)	.54	.75
Frontal lobe left gray matter	-0.003 (-0.01-0.004)	.37	.63
Frontal lobe left white matter	0.003 (-0.01-0.02)	.34	.60
Frontal lobe right gray matter	-0.004 (-0.01-0.003)	.26	.54
Frontal lobe right white matter	0.004 (-0.02-0.02)	.31	.59
Gyri parahippocampalis et ambiens anterior part left gray matter	-0.001 (-0.01-0.01)	.72	.84
Gyri parahippocampalis et ambiens anterior part left white matter	0.002 (-0.02-0.02)	.68	.82
Gyri parahippocampalis et ambiens anterior part right gray matter	-0.002 (-0.01-0.01)	.53	.75
Gyri parahippocampalis et ambiens anterior part right white matter	-0.004 (-0.01-0.01)	.36	.62
Gyri parahippocampalis et ambiens posterior part left gray matter	0.001 (-0.01-0.01)	.77	.87
Gyri parahippocampalis et ambiens posterior part left white matter	0.003 (-0.01-0.01)	.44	.69
Gyri parahippocampalis et ambiens posterior part right gray matter	0.01 (-0.001-0.02)	.04	.15
Gyri parahippocampalis et ambiens posterior part right white matter	0.004 (-0.02-0.03)	.26	.54
Hippocampus left	0.005 (-0.01-0.02)	.21	.46
Hippocampus right	-0.001 (-0.01-0.01)	.72	.84
Insula left gray matter	-0.005 (-0.01-0.003)	.19	.46
Insula left white matter	0.01 (-0.002-0.01)	.08	.28
Insula right gray matter	0.01 (-0.01-0.03)	.01	.08
Insula right white matter	0.004 (-0.02-0.02)	.30	.59
Lateral occipitotemporal gyrus gyrus fusiformis anterior part left gray matter	-0.01 (-0.03-0.01)	.07	.24

© 2023 Mckinnon K et al. *JAMA Network Open.*

Regional volume	Standardized β coefficient (95% confidence intervals)	Raw *p* value	BH corrected *p* value
Lateral occipitotemporal gyrus gyrus fusiformis anterior part left white matter	-0.004 (-0.01-0.005)	.32	.59
Lateral occipitotemporal gyrus gyrus fusiformis anterior part right gray matter	-0.001 (-0.01-0.01)	.84	.91
Lateral occipitotemporal gyrus gyrus fusiformis anterior part right white matter	-0.003 (-0.01-0.01)	.46	.71
Lateral occipitotemporal gyrus gyrus fusiformis posterior part left gray matter	0.003 (-0.01-0.01)	.44	.70
Lateral occipitotemporal gyrus gyrus fusiformis posterior part left white matter	0.01 (-0.01-0.03)	.01	.06
Lateral occipitotemporal gyrus gyrus fusiformis posterior part right gray matter	0.002 (-0.01-0.01)	.58	.78
Lateral occipitotemporal gyrus gyrus fusiformis posterior part right white matter	0.00005 (-0.01-0.01)	.99	.99
Lateral ventricle left	0.001 (-0.02-0.02)	.60	.78
Lateral ventricle right	0.001 (-0.02-0.02)	.52	.75
Lentiform nucleus left	-0.002 (-0.01-0.01)	.65	.80
Lentiform nucleus right	0.01 (-0.01-0.03)	.048	.17
Medial and inferior temporal gyri anterior part left gray matter	-0.004 (-0.01-0.003)	.25	.52
Medial and inferior temporal gyri anterior part left white matter	0.002 (-0.01-0.01)	.53	.75
Medial and inferior temporal gyri anterior part right gray matter	-0.004 (-0.02-0.01)	.33	.59
Medial and inferior temporal gyri anterior part right white matter	0.0003 (-0.01-0.01)	.94	.97
Medial and inferior temporal gyri posterior part left gray matter	-0.0002 (-0.01-0.01)	.94	.97
Medial and inferior temporal gyri posterior part left white matter	0.01 (-0.003-0.02)	.11	.33
Medial and inferior temporal gyri posterior part right gray matter	0.002 (-0.004-0.01)	.55	.75
Medial and inferior temporal gyri posterior part right white matter	0.003 (-0.01-0.01)	.47	.72
Occipital lobe left gray matter	0.0005 (-0.01-0.01)	.88	.94
Occipital lobe left white matter	0.004 (-0.01-0.01)	.31	.59
Occipital lobe right gray matter	0.002 (-0.005-0.01)	.61	.79
Occipital lobe right white matter	0.003 (-0.01-0.01)	.42	.67
Parietal lobe left gray matter	-0.001 (-0.01-0.004)	.60	.78
Parietal lobe left white matter	0.003 (-0.01-0.01)	.51	.75
Parietal lobe right gray matter	-0.001 (-0.01-0.004)	.60	.78
Parietal lobe right white matter	0.001 (-0.01-0.01)	.72	.84
Subthalamic nucleus left	-0.002 (-0.01-0.01)	.59	.78
Subthalamic nucleus right	-0.01 (-0.01-0.004)	.16	.40
Superior temporal gyrus middle part left gray matter	-0.0005 (-0.01-0.01)	.90	.95
Superior temporal gyrus middle part left white matter	-0.001 (-0.01-0.01)	.76	.87
Superior temporal gyrus middle part right gray matter	0.0004 (-0.01-0.01)	.91	.96
Superior temporal gyrus middle part right white matter	-0.001 (-0.01-0.01)	.69	.82
Superior temporal gyrus posterior part left gray matter	-0.01 (-0.01-0.002)	.14	.37
Superior temporal gyrus posterior part left white matter	0.003 (-0.01-0.01)	.32	.59
Superior temporal gyrus posterior part right gray matter	-0.004 (-0.01-0.004)	.31	.59
Superior temporal gyrus posterior part right white matter	-0.004 (-0.01-0.01)	.33	.59

© 2023 Mckinnon K et al. *JAMA Network Open.*

**Regional volume**	**Standardized β coefficient (95% confidence intervals)**	**Raw *p* value**	**BH corrected *p* value**
Thalamus left high intensity part in T2	0.002 (-0.005-0.01)	.50	.74
Thalamus left low intensity part in T2	0.002 (-0.01-0.01)	.29	.57
Thalamus right high intensity part in T2	0.002 (-0.01-0.01)	.63	.79
Thalamus right low intensity part in T2	0.001 (-0.01-0.01)	.83	.91

Fully adjusted ridge regression model, including gestation at birth, subjective socioeconomic status, gestation at MRI, the interaction term (where significant), birth weight z-score, birth head circumference z-score, sex, smoking in pregnancy, and breast milk at discharge. Corrected for false discovery rate.
BH = Benjamini-Hochberg correction.

© 2023 Mckinnon K et al. *JAMA Network Open.*

eTable 11. Cortical Measures: Association With Gestational Age and Maternal Final Educational Qualification (Fully Adjusted Ridge Regression Model)

Cortical measure	Standardized β coefficient (95% confidence intervals)	Raw *p* value	BH corrected *p* value
Gestation			
Mean cortical curvature	-0.0001 (-0.10-0.10)	.63	.84
Mean cortical surface area	0.01 (-0.11-0.12)	.48	.84
Mean cortical thickness	-0.002 (-0.10-0.09)	.61	.84
Mean gyrification index	0.005 (-0.11-0.12)	.48	.84
Mean sulcal depth	0.003 (-0.12-0.12)	.92	.92
Maternal education			
Mean cortical curvature	0.0001 (-0.09-0.09)	.52	.84
Mean cortical surface area	-0.01 (-0.11-0.09)	.22	.84
Mean cortical thickness	0.004 (-0.08-0.09)	.15	.84
Mean gyrification index	-0.002 (-0.10-0.10)	.77	.85
Mean sulcal depth	0.01 (-0.11-0.13)	.67	.84

Fully adjusted ridge regression model, including gestation at birth, maternal final educational qualification, gestation at MRI, the interaction term (where significant – none were, so not included), birth weight z-score, birth head circumference z-score, sex, smoking in pregnancy, and breast milk at discharge. Corrected for false discovery rate.
BH = Benjamini-Hochberg correction.

© 2023 Mckinnon K et al. *JAMA Network Open.*

eTable 12. Cortical Measures: Association With Gestational Age and Paternal Final Educational Qualification (Fully Adjusted Ridge Regression Model)

Cortical measure	Standardized β coefficient (95% confidence intervals)	Raw *p* value	BH corrected *p* value
Gestation			
Mean cortical curvature	-0.01 (-0.13-0.10)	.67	.74
Mean cortical surface area	0.01 (-0.12-0.13)	.63	.74
Mean cortical thickness	-0.01 (-0.12-0.10)	.38	.74
Mean gyrification index	0.01 (-0.11-0.13)	.49	.74
Mean sulcal depth	0.01 (-0.12-0.14)	.76	.76
Paternal education			
Mean cortical curvature	-0.04 (-0.14-0.06)	.18	.74
Mean cortical surface area	0.01 (-0.09-0.11)	.60	.74
Mean cortical thickness	0.01 (-0.08-0.10)	.48	.74
Mean gyrification index	0.01 (-0.09-0.11)	.41	.74
Mean sulcal depth	-0.02 (-0.13-0.09)	.59	.74

Fully adjusted ridge regression model, including gestation at birth, paternal final educational qualification, gestation at MRI, the interaction term (where significant – none were, so not included), birth weight z-score, birth head circumference z-score, sex, smoking in pregnancy, and breast milk at discharge. Corrected for false discovery rate.
BH = Benjamini-Hochberg correction.

© 2023 Mckinnon K et al. *JAMA Network Open.*

eTable 13. Cortical Measures: Association With Gestational Age, Maternal Occupation, and Interaction Effect (Fully Adjusted Ridge Regression Model)

Cortical measure	Standardized β coefficient (95% confidence intervals)	Raw *p* value	BH corrected *p* value
Gestation			
Mean cortical curvature	-0.004 (-0.11-0.10)	.41	.74
Mean cortical surface area	0.01 (-0.10-0.12)	.46	.74
Mean cortical thickness	-0.001 (-0.11-0.11)	.51	.74
Mean gyrification index	0.01 (-0.10-0.12)	.54	.74
Mean sulcal depth	0.005 (-0.11-0.12)	.88	.88
Interaction			
Mean gyrification index	0.01 (-0.06-0.07)	.02	.27
Maternal occupation			
Mean cortical curvature	0.001 (-0.06-0.06)	.81	.88
Mean cortical surface area	-0.01 (-0.07-0.06)	.44	.74
Mean cortical thickness	0.0004 (-0.06-0.06)	.75	.88
Mean gyrification index	-0.01 (-0.08-0.06)	.40	.74
Mean sulcal depth	0.03 (-0.05-0.10)	.20	.74

Fully adjusted ridge regression model, including gestation at birth, maternal occupation, gestation at MRI, the interaction term (where significant), birth weight z-score, birth head circumference z-score, sex, smoking in pregnancy, and breast milk at discharge. Corrected for false discovery rate.
BH = Benjamini-Hochberg correction.

© 2023 Mckinnon K et al. *JAMA Network Open.*

eTable 14. Cortical Measures: Association With Gestational Age and Paternal Occupation (Fully Adjusted Ridge Regression Model)

Cortical measure	Standardized β coefficient (95% confidence intervals)	Raw *p* value	BH corrected *p* value
Gestation			
Mean cortical curvature	-0.02 (-0.13-0.09)	.56	.92
Mean cortical surface area	0.01 (-0.10-0.12)	.67	.92
Mean cortical thickness	-0.001 (-0.11-0.11)	.61	.92
Mean gyrification index	0.01 (-0.10-0.12)	.53	.92
Mean sulcal depth	0.01 (-0.11-0.13)	.79	.92
Paternal occupation			
Mean cortical curvature	-0.002 (-0.07-0.07)	.92	.92
Mean cortical surface area	0.01 (-0.06-0.09)	.45	.92
Mean cortical thickness	-0.001 (-0.06-0.06)	.66	.92
Mean gyrification index	0.01 (-0.06-0.09)	.46	.92
Mean sulcal depth	-0.01 (-0.09-0.08)	.83	.92

Fully adjusted ridge regression model, including gestation at birth, paternal occupation, gestation at MRI, the interaction term (where significant – none were, so not included), birth weight z-score, birth head circumference z-score, sex, smoking in pregnancy, and breast milk at discharge. Corrected for false discovery rate.
BH = Benjamini-Hochberg correction.

© 2023 Mckinnon K et al. *JAMA Network Open.*

eTable 15. Cortical Measures: Association With Gestational Age, Subjective Socioeconomic Status, and Interaction Effect (Fully Adjusted Ridge Regression Model)

Cortical measure	Standardized β coefficient (95% confidence intervals)	Raw *p* value	BH corrected *p* value
Gestation			
Mean cortical curvature	0.004 (-0.08-0.09)	.92	.92
Mean cortical surface area	0.09 (0.003-0.17)	.03	.11
Mean cortical thickness	-0.01 (-0.12-0.09)	.75	.92
Mean gyrification index	0.16 (0.07-0.26)	<.001	.002
Mean sulcal depth	0.04 (-0.40-0.48)	.10	.27
Interaction			
Mean sulcal depth	0.0005 (-0.01-0.01)	.02	.11
Subjective socioeconomic status			
Mean cortical curvature	-0.002 (-0.01-0.01)	.60	.92
Mean cortical surface area	0.001 (-0.01-0.01)	.81	.92
Mean cortical thickness	-0.002 (-0.01-0.005)	.67	.92
Mean gyrification index	-0.001 (-0.01-0.01)	.86	.92
Mean sulcal depth	0.004 (-0.01-0.02)	.15	.33

Fully adjusted ridge regression model, including gestation at birth, subjective socioeconomic status (as World Health Organisation Quality of Life, environment domain), gestation at MRI, the interaction term (where significant), birth weight z-score, birth head circumference z-score, sex, smoking in pregnancy, and breast milk at discharge. Corrected for false discovery rate.
BH = Benjamini-Hochberg correction.

© 2023 Mckinnon K et al. *JAMA Network Open.*

eTable 16. Lobar Volumes: Association With Gestational Age and the Scottish Index of Multiple Deprivation (Fully Adjusted Ridge Regression Models)

Regional volume	Standardized β coefficient (95% confidence intervals)	Raw *p* value	BH corrected *p* value
Gestation			
Deep grey matter	0.06 (-0.01-0.13)	.08	.23
Frontal lobe left	0.02 (-0.05-0.10)	.57	.62
Frontal lobe right	0.11 (0.04-0.18)	.001	.01
Occipital lobe left	-0.05 (-0.13-0.03)	.20	.38
Occipital lobe right	-0.02 (-0.10-0.06)	.59	.62
Parietal lobe left	0.10 (0.03-0.17)	.002	.01
Parietal lobe right	0.10 (0.03-0.16)	.004	.02
Temporal lobe left	0.10 (0.03-0.17)	.002	.01
Temporal lobe right	0.09 (0.03-0.16)	.005	.02
Whole brain	0.08 (0.01-0.14)	.01	.04
SIMD			
Deep grey matter	-0.03 (-0.12-0.06)	.48	.57
Frontal lobe left	0.07 (-0.03-0.16)	.16	.36
Frontal lobe right	0.08 (-0.02-0.17)	.10	.24
Occipital lobe left	0.01 (-0.09-0.12)	.76	.76
Occipital lobe right	0.04 (-0.07-0.15)	.45	.57
Parietal lobe left	0.11 (-0.12-0.34)	.21	.38
Parietal lobe right	0.03 (-0.06-0.12)	.49	.57
Temporal lobe left	0.04 (-0.05-0.13)	.39	.56
Temporal lobe right	0.04 (-0.05-0.13)	.33	.51
Whole brain	0.05 (-0.03-0.13)	.23	.38

Fully adjusted ridge regression model, including gestation at birth, Scottish Index of Multiple Deprivation, gestation at MRI, the interaction term (where significant – none were, so not included), birth weight z-score, birth head circumference z-score, sex, smoking in pregnancy, and breast milk at discharge. Corrected for false discovery rate.
BH = Benjamini-Hochberg correction, SIMD = Scottish Index of Multiple Deprivation.

© 2023 Mckinnon K et al. *JAMA Network Open.*

eTable 17. Lobar Volumes: Association With Gestational Age and Maternal Education (Fully Adjusted Ridge Regression Models)

Regional volume	Standardized β coefficient (95% confidence intervals)	Raw *p* value	BH corrected *p* value
Gestation			
Deep grey matter	0.04 (-0.03-0.11)	.27	.34
Frontal lobe left	0.02 (-0.06-0.10)	.61	.64
Frontal lobe right	0.11 (0.04-0.19)	.002	.03
Occipital lobe left	-0.06 (-0.14-0.02)	.15	.26
Occipital lobe right	-0.03 (-0.11-0.06)	.50	.55
Parietal lobe left	0.09 (0.02-0.16)	.01	.045
Parietal lobe right	0.08 (0.01-0.15)	.02	.06
Temporal lobe left	0.10 (0.03-0.17)	.004	.04
Temporal lobe right	0.08 (0.02-0.15)	.01	.06
Whole brain	0.07 (0.004-0.14)	.03	.11
Maternal education			
Deep grey matter	0.04 (-0.03-0.12)	.19	.32
Frontal lobe left	-0.01 (-0.09-0.07)	.88	.88
Frontal lobe right	0.03 (-0.05-0.10)	.47	.55
Occipital lobe left	0.04 (-0.04-0.12)	.25	.34
Occipital lobe right	0.07 (-0.02-0.15)	.10	.24
Parietal lobe left	0.04 (-0.03-0.11)	.23	.34
Parietal lobe right	0.04 (-0.03-0.11)	.26	.34
Temporal lobe left	0.05 (-0.02-0.13)	.11	.24
Temporal lobe right	0.06 (-0.01-0.12)	.08	.23
Whole brain	0.05 (-0.02-0.12)	.12	.24

Fully adjusted ridge regression model, including gestation at birth, maternal final educational qualification, gestation at MRI, the interaction term (where significant – none were, so not included), birth weight z-score, birth head circumference z-score, sex, smoking in pregnancy, and breast milk at discharge. Corrected for false discovery rate.
BH = Benjamini-Hochberg correction.

© 2023 Mckinnon K et al. *JAMA Network Open.*

eTable 18. Lobar Volumes: Association With Gestational Age and Paternal Education (Fully Adjusted Ridge Regression Models)

Regional volume	Standardized β coefficient (95% confidence intervals)	Raw *p* value	BH corrected *p* value
Gestation			
Deep grey matter	0.06 (-0.02-0.13)	.13	.31
Frontal lobe left	0.03 (-0.06-0.11)	.51	.86
Frontal lobe right	0.13 (0.05-0.20)	.001	.01
Occipital lobe left	-0.03 (-0.12-0.05)	.40	.81
Occipital lobe right	0.001 (-0.09-0.09)	.99	.99
Parietal lobe left	0.11 (0.04-0.18)	.002	.01
Parietal lobe right	0.10 (0.03-0.18)	.004	.01
Temporal lobe left	0.11 (0.04-0.19)	.002	.01
Temporal lobe right	0.10 (0.03-0.18)	.003	.01
Whole brain	0.09 (0.02-0.16)	.01	.03
Paternal education			
Deep grey matter	0.002 (-0.06-0.06)	.93	.98
Frontal lobe left	-0.01 (-0.08-0.05)	.67	.89
Frontal lobe right	0.004 (-0.06-0.07)	.88	.98
Occipital lobe left	-0.02 (-0.09-0.05)	.59	.89
Occipital lobe right	-0.01 (-0.08-0.07)	.82	.98
Parietal lobe left	0.01 (-0.05-0.07)	.67	.89
Parietal lobe right	0.01 (-0.06-0.07)	.85	.98
Temporal lobe left	0.05 (-0.02-0.11)	.14	.31
Temporal lobe right	0.05 (-0.01-0.11)	.10	.29
Whole brain	0.02 (-0.04-0.08)	.52	.86

Fully adjusted ridge regression model, including gestation at birth, paternal final educational qualification, gestation at MRI, the interaction term (where significant – none were, so not included), birth weight z-score, birth head circumference z-score, sex, smoking in pregnancy, and breast milk at discharge. Corrected for false discovery rate.
BH = Benjamini-Hochberg correction.

© 2023 Mckinnon K et al. *JAMA Network Open.*

eTable 19. Lobar Volumes: Association With Gestational Age and Maternal Occupation (Fully Adjusted Ridge Regression Models)

Regional volume	Standardized β coefficient (95% confidence intervals)	Raw *p* value	BH corrected *p* value
Gestation			
Deep grey matter	0.05 (-0.02-0.12)	.14	.26
Frontal lobe left	0.02 (-0.06-0.09)	.61	.61
Frontal lobe right	0.11 (0.04-0.18)	.001	.02
Occipital lobe left	-0.06 (-0.14-0.01)	.09	.20
Occipital lobe right	-0.03 (-0.12-0.05)	.38	.45
Parietal lobe left	0.10 (0.03-0.16)	.003	.02
Parietal lobe right	0.09 (0.02-0.16)	.01	.03
Temporal lobe left	0.10 (0.03-0.17)	.003	.02
Temporal lobe right	0.09 (0.02-0.16)	.01	.03
Whole brain	0.07 (0.01-0.14)	.02	.07
Maternal occupation			
Deep grey matter	0.02 (-0.02-0.07)	.33	.43
Frontal lobe left	0.01 (-0.03-0.06)	.54	.60
Frontal lobe right	0.01 (-0.04-0.06)	.61	.61
Occipital lobe left	0.05 (-0.003-0.10)	.06	.14
Occipital lobe right	0.06 (0.002-0.11)	.03	.09
Parietal lobe left	0.02 (-0.02-0.06)	.35	.43
Parietal lobe right	0.03 (-0.02-0.07)	.21	.30
Temporal lobe left	0.03 (-0.01-0.08)	.14	.26
Temporal lobe right	0.03 (-0.01-0.07)	.17	.29
Whole brain	0.03 (-0.01-0.07)	.19	.29

Fully adjusted ridge regression model, including gestation at birth, maternal occupation, gestation at MRI, the interaction term (where significant – none were, so not included), birth weight z-score, birth head circumference z-score, sex, smoking in pregnancy, and breast milk at discharge. Corrected for false discovery rate.
BH = Benjamini-Hochberg correction.

© 2023 Mckinnon K et al. *JAMA Network Open.*

eTable 20. Lobar Volumes: Association With Gestational Age and Paternal Occupation (Fully Adjusted Ridge Regression Models)

**Regional volume**	**Standardized β coefficient (95% confidence intervals)**	**Raw *p* value**	**BH corrected *p* value**
Gestation			
Deep grey matter	0.06 (-0.01-0.13)	.10	.29
Frontal lobe left	0.02 (-0.05-0.10)	.56	.70
Frontal lobe right	0.11 (0.04-0.19)	.001	.02
Occipital lobe left	-0.05 (-0.13-0.03)	.19	.47
Occipital lobe right	-0.02 (-0.11-0.06)	.59	.70
Parietal lobe left	0.10 (0.03-0.17)	.003	.02
Parietal lobe right	0.09 (0.03-0.16)	.005	.02
Temporal lobe left	0.10 (0.03-0.17)	.003	.02
Temporal lobe right	0.09 (0.03-0.16)	.01	.02
Whole brain	0.08 (0.01-0.15)	.01	.045
Paternal occupation			
Deep grey matter	-0.02 (-0.07-0.03)	.45	.65
Frontal lobe left	0.01 (-0.05-0.06)	.83	.87
Frontal lobe right	0.03 (-0.03-0.08)	.32	.63
Occipital lobe left	0.01 (-0.05-0.06)	.77	.85
Occipital lobe right	-0.005 (-0.06-0.06)	.87	.87
Parietal lobe left	0.03 (-0.02-0.08)	.22	.48
Parietal lobe right	0.02 (-0.03-0.07)	.36	.63
Temporal lobe left	0.02 (-0.03-0.07)	.52	.69
Temporal lobe right	0.02 (-0.03-0.07)	.38	.63
Whole brain	0.02(-0.03-0.06)	.45	.65

Fully adjusted ridge regression model, including gestation at birth, paternal occupation, gestation at MRI, the interaction term (where significant – none were, so not included), birth weight z-score, birth head circumference z-score, sex, smoking in pregnancy, and breast milk at discharge. Corrected for false discovery rate.
BH = Benjamini-Hochberg correction.

© 2023 Mckinnon K et al. *JAMA Network Open.*

eTable 21. Lobar Volumes: Association With Gestational Age and Subjective Socioeconomic Status (Fully Adjusted Ridge Regression Models)

Regional volume	Standardized β coefficient (95% confidence intervals)	Raw *p* value	BH corrected *p* value
Gestation			
Deep grey matter	0.03 (-0.04-0.11)	.37	.68
Frontal lobe left	0.02 (-0.06-0.10)	.55	.79
Frontal lobe right	0.10 (0.02-0.18)	.01	.049
Occipital lobe left	-0.07 (-0.15-0.01)	.09	.25
Occipital lobe right	-0.05 (-0.14-0.04)	.22	.56
Parietal lobe left	0.10 (0.03-0.17)	.005	.049
Parietal lobe right	0.09 (0.02-0.16)	.01	.049
Temporal lobe left	0.10 (0.02-0.17)	.01	.049
Temporal lobe right	0.09 (0.01-0.16)	.02	.06
Whole brain	0.07 (-0.001-0.14)	.046	.15
Subjective socioeconomic status			
Deep grey matter	0.003 (-0.004-0.01)	.37	.68
Frontal lobe left	-0.003 (-0.01-0.004)	.37	.68
Frontal lobe right	0.001 (-0.01-0.01)	.69	.87
Occipital lobe left	0.002 (-0.005-0.01)	.50	.77
Occipital lobe right	0.003 (-0.005-0.01)	.44	.73
Parietal lobe left	<0.001 (-0.01-0.01)	.88	.99
Parietal lobe right	<0.001 (-0.01-0.01)	.96	.99
Temporal lobe left	<0.001 (-0.01-0.01)	.99	.99
Temporal lobe right	<0.001 (-0.01-0.01)	.96	.99
Whole brain	0.001 (-0.005-0.01)	.64	.85

Fully adjusted ridge regression model, including gestation at birth, subjective socioeconomic status, gestation at MRI, the interaction term (where significant – none were, so not included), birth weight z-score, birth head circumference z-score, sex, smoking in pregnancy, and breast milk at discharge. Corrected for false discovery rate.
BH = Benjamini-Hochberg correction.

© 2023 Mckinnon K et al. *JAMA Network Open.*

eTable 22. Cortical Measures: Association With Gestational Age, the Scottish Index of Multiple Deprivation, and Interaction Effect (Fully Adjusted Ridge Regression Models)

Cortical measure	Standardized β coefficient (95% confidence intervals)	Raw *p* value	BH corrected *p* value
Gestation			
Frontal lobe left – curvature	0.01 (-0.08-0.11)	.73	.84
Frontal lobe right – curvature	0.03 (-0.05-0.12)	.42	.61
Occipital lobe left – curvature	-0.07 (-0.18-0.03)	.049	.39
Occipital lobe right – curvature	0.01 (-0.10-0.11)	.64	.84
Parietal lobe left – curvature	-0.05 (-0.15-0.05)	.23	.52
Parietal lobe right – curvature	-0.04 (-0.14-0.06)	.32	.56
Temporal lobe left – curvature	-0.003 (-0.09-0.09)	.95	.95
Temporal lobe right – curvature	-0.04 (-0.14-0.06)	.35	.57
Frontal lobe left – surface area	0.16 (0.08-0.24)	<.001	<.001
Frontal lobe right – surface area	0.14 (0.06-0.22)	<.001	<.001
Occipital lobe left – surface area	0.06 (-0.04-0.15)	.18	.30
Occipital lobe right – surface area	0.06 (-0.04-0.15)	.18	.30
Parietal lobe left – surface area	0.15 (0.06-0.23)	<.001	<.001
Parietal lobe right – surface area	0.11 (0.02-0.19)	.01	.02
Temporal lobe left – surface area	0.11 (0.03-0.19)	.004	.02
Temporal lobe right – surface area	0.10 (0.02-0.18)	.01	.03
Frontal lobe left – thickness	-0.08 (-0.18-0.02)	.004	.07
Frontal lobe right – thickness	-0.07 (-0.18-0.03)	.02	.14
Occipital lobe left – thickness	-0.02 (-0.09-0.04)	.55	.67
Occipital lobe right – thickness	-0.01 (-0.10-0.08)	.86	.86
Parietal lobe left – thickness	-0.02 (-0.12-0.07)	.60	.68
Parietal lobe right – thickness	0.02 (-0.08-0.11)	.71	.75
Temporal lobe left – thickness	0.07 (-0.03-0.16)	.11	.44
Temporal lobe right – thickness	0.03 (-0.06-0.13)	.44	.67
Frontal lobe left – gyrification index	0.02 (-0.10-0.13)	.28	.64
Frontal lobe right – gyrification index	0.01 (-0.11-0.12)	.40	.65
Occipital lobe left – gyrification index	-0.02 (-0.14-0.09)	.42	.65
Occipital lobe right – gyrification index	-0.01 (-0.11-0.10)	.23	.64
Parietal lobe left – gyrification index	0.02 (-0.08-0.13)	.07	.57
Parietal lobe right – gyrification index	-0.002 (-0.10-0.09)	.02	.31
Temporal lobe left – gyrification index	0.02 (-0.10-0.15)	.15	.64
Temporal lobe right – gyrification index	0.02 (-0.10-0.13	.19	.64
Frontal lobe left – sulcal depth	0.06 (-0.05-0.17)	.051	.28
Frontal lobe right – sulcal depth	-0.04 (-0.14-0.07)	.18	.50
Occipital lobe left – sulcal depth	-0.05 (-0.15-0.05)	.26	.50

© 2023 Mckinnon K et al. *JAMA Network Open.*

**Cortical measure**	**Standardized β coefficient (95% confidence intervals)**	**Raw *p* value**	**BH corrected *p* value**
Occipital lobe right – sulcal depth	-0.01 (-0.11-0.10)	.85	.88
Parietal lobe left – sulcal depth	-0.02 (-0.12-0.07)	.58	.82
Parietal lobe right – sulcal depth	-0.004 (-0.10-0.09)	.88	.88
Temporal lobe left – sulcal depth	-0.08 (-0.18-0.01)	.03	.26
Temporal lobe right – sulcal depth	-0.05 (-0.15-0.04)	.22	.50
Interaction			
Frontal lobe right – thickness	-0.02 (-0.11-0.07)	.04	.20
Parietal lobe right – sulcal depth	-0.03 (-0.12-0.07)	.001	.01
SIMD			
Frontal lobe left – curvature	-0.09 (-0.21-0.03)	.12	.49
Frontal lobe right – curvature	-0.07 (-0.19-0.04)	.18	.49
Occipital lobe left – curvature	-0.05 (-0.19-0.09)	.30	.56
Occipital lobe right – curvature	-0.03 (-0.17-0.11)	.18	.49
Parietal lobe left – curvature	-0.08 (-0.20-0.05)	.16	.49
Parietal lobe right – curvature	0.02 (-0.11-0.15)	.69	.84
Temporal lobe left – curvature	-0.01 (-0.13-0.11)	.86	.91
Temporal lobe right – curvature	-0.14 (-0.27- -0.01)	.01	.23
Frontal lobe left – surface area	0.04 (-0.06-0.14)	.39	.41
Frontal lobe right – surface area	0.06 (-0.05-0.16)	.25	.33
Occipital lobe left – surface area	0.05 (-0.08-0.17)	.41	.41
Occipital lobe right – surface area	0.06 (-0.06-0.19)	.28	.33
Parietal lobe left – surface area	0.05 (-0.05-0.16)	.29	.33
Parietal lobe right – surface area	0.06 (-0.05-0.17)	.27	.33
Temporal lobe left – surface area	0.07 (-0.04-0.18)	.19	.30
Temporal lobe right – surface area	0.08 (-0.02-0.18)	.11	.26
Frontal lobe left – thickness	-0.03 (-0.17-0.11)	.42	.67
Frontal lobe right – thickness	-0.02 (-0.37-0.32)	.52	.67
Occipital lobe left – thickness	-0.04 (-0.16-0.08)	.47	.67
Occipital lobe right – thickness	-0.04 (-0.16-0.09)	.53	.67
Parietal lobe left – thickness	-0.04 (-0.17-0.09)	.45	.67
Parietal lobe right – thickness	-0.08 (-0.21-0.06)	.19	.54
Temporal lobe left – thickness	-0.09 (-0.21-0.04)	.13	.44
Temporal lobe right – thickness	0.05 (-0.28-0.37	.45	.67
Frontal lobe left – gyrification index	0.02 (-0.13-0.17)	.39	.65
Frontal lobe right – gyrification index	0.01 (-0.15-0.17)	.44	.65
Occipital lobe left – gyrification index	-0.003 (-0.17-0.16)	.93	.95
Occipital lobe right – gyrification index	-0.01 (-0.17-0.16)	.25	.64
Parietal lobe left – gyrification index	0.002 (-0.15-0.15)	.92	.95
Parietal lobe right – gyrification index	<0.001 (-0.16-0.16)	.94	.95

© 2023 Mckinnon K et al. *JAMA Network Open.*

Cortical measure	Standardized β coefficient (95% confidence intervals)	Raw *p* value	BH corrected *p* value
Temporal lobe left – gyrification index	-0.002 (-0.17-0.17)	.95	.95
Temporal lobe right – gyrification index	-0.01 (-0.16-0.14)	.58	.77
Frontal lobe left – sulcal depth	-0.02 (-0.16-0.13)	.70	.82
Frontal lobe right – sulcal depth	-0.02 (-0.16-0.12)	.67	.82
Occipital lobe left – sulcal depth	-0.02 (-0.16-0.12)	.72	.82
Occipital lobe right – sulcal depth	-0.06 (-0.20-0.08)	.23	.50
Parietal lobe left – sulcal depth	0.06 (-0.07-0.20)	.26	.50
Parietal lobe right – sulcal depth	-0.01 (-0.37-0.34)	.63	.82
Temporal lobe left – sulcal depth	-0.07 (-0.20-0.06)	.16	.50
Temporal lobe right – sulcal depth	-0.03 (-0.16-0.10)	.63	.82

Fully adjusted ridge regression model, including gestation at birth, Scottish Index of Multiple Deprivation, gestation at MRI, the interaction term (where significant), birth weight z-score, birth head circumference z-score, sex, smoking in pregnancy, and breast milk at discharge. Corrected for false discovery rate.
BH = Benjamini-Hochberg correction, SIMD = Scottish Index of Multiple Deprivation.

© 2023 Mckinnon K et al. *JAMA Network Open.*

eTable 23. Cortical Measures: Association With Gestational Age, Maternal Education, and Interaction Effect (Fully Adjusted Ridge Regression Models)

Cortical measure	Standardized β coefficient (95% confidence intervals)	Raw *p* value	BH corrected *p* value
Gestation			
Frontal lobe left – curvature	0.03 (-0.07-0.13)	.50	.95
Frontal lobe right – curvature	0.03 (-0.06-0.12)	.51	.95
Occipital lobe left – curvature	-0.05 (-0.34-0.25)	.08	.30
Occipital lobe right – curvature	0.002 (-0.11-0.11)	.84	.95
Parietal lobe left – curvature	-0.07 (-0.17-0.03)	.09	.30
Parietal lobe right – curvature	-0.03 (-0.13-0.08)	.51	.95
Temporal lobe left – curvature	-0.002 (-0.10-0.09)	.97	.97
Temporal lobe right – curvature	-0.05 (-0.15-0.05)	.24	.67
Frontal lobe left – surface area	0.06 (-0.20-0.32)	.24	.45
Frontal lobe right – surface area	0.07 (-0.20-0.34)	.18	.40
Occipital lobe left – surface area	0.06 (-0.04-0.16)	.16	.38
Occipital lobe right – surface area	0.02 (-0.33-0.30)	.78	.86
Parietal lobe left – surface area	0.06 (-0.21-0.33)	.23	.45
Parietal lobe right – surface area	0.03 (-0.25-0.30)	.61	.81
Temporal lobe left – surface area	0.03 (-0.25-0.30)	.62	.81
Temporal lobe right – surface area	0.10 (0.02-0.18)	.01	.09
Frontal lobe left – thickness	-0.06 (-0.38-0.26)	.10	.45
Frontal lobe right – thickness	-0.05 (-0.37-0.27)	.21	.53
Occipital lobe left – thickness	-0.04 (-0.14-0.06)	.33	.59
Occipital lobe right – thickness	-0.02 (-0.12-0.07)	.59	.75
Parietal lobe left – thickness	-0.03 (-0.13-0.06)	.45	.71
Parietal lobe right – thickness	0.002 (-0.09-0.09)	.97	.97
Temporal lobe left – thickness	0.06 (-0.04-0.15)	.18	.53
Temporal lobe right – thickness	0.04 (-0.05-0.14)	.33	.59
Frontal lobe left – gyrification index	0.04 (-0.08-0.15)	.15	.36
Frontal lobe right – gyrification index	<0.001 (-0.11-0.11)	.45	.62
Occipital lobe left – gyrification index	-0.02 (-0.13-0.10)	.56	.72
Occipital lobe right – gyrification index	-0.01 (-0.14-0.11)	.20	.36
Parietal lobe left – gyrification index	0.04 (-0.15-0.24)	.09	.33
Parietal lobe right – gyrification index	-0.06 (-0.30-0.18)	.01	.13
Temporal lobe left – gyrification index	0.02 (-0.11-0.16)	.16	.36
Temporal lobe right – gyrification index	0.02 (-0.11-0.14)	.20	.36
Frontal lobe left – sulcal depth	0.05 (-0.31-0.40)	.10	.26
Frontal lobe right – sulcal depth	-0.09 (-0.19-0.02)	.06	.26
Occipital lobe left – sulcal depth	-0.04 (-0.15-0.06)	.27	.47
Occipital lobe right – sulcal depth	-0.03 (-0.14-0.08)	.48	.55

© 2023 Mckinnon K et al. *JAMA Network Open.*

Cortical measure	Standardized β coefficient (95% confidence intervals)	Raw *p* value	BH corrected *p* value
Parietal lobe left – sulcal depth	-0.03 (-0.13-0.07)	.49	.55
Parietal lobe right – sulcal depth	0.01 (-0.16-0.17)	.71	.71
Temporal lobe left – sulcal depth	-0.02 (-0.13-0.08)	.20	.41
Temporal lobe right – sulcal depth	-0.07 (-0.17-0.03)	.15	.33
Interaction			
Occipital lobe left – curvature	-0.01 (-0.06-0.05)	.04	.30
Frontal lobe left – surface area	0.02 (-0.03-0.07)	.01	.09
Frontal lobe right – surface area	0.02 (-0.03-0.06)	.04	.12
Occipital lobe right – surface area	0.02 (-0.04-0.07)	.003	.08
Parietal lobe left – surface area	0.02 (-0.03-0.07)	.02	.09
Parietal lobe right – surface area	0.02 (-0.03-0.06)	.04	.12
Temporal lobe left – surface area	0.02 (-0.03-0.07)	.03	.12
Frontal lobe left – thickness	-0.02 (-0.07-0.04)	<.001	.01
Frontal lobe right – thickness	-0.02 (-0.07-0.04)	<.001	.01
Parietal lobe left – gyrification index	0.01 (-0.03-0.04)	.02	.13
Parietal lobe right – gyrification index	-0.01 (-0.05-0.04)	.02	.13
Frontal lobe left – sulcal depth	0.01 (-0.06-0.07)	.03	.16
Parietal lobe right – sulcal depth	-0.01 (-0.04-0.03)	.01	.12
Maternal education			
Frontal lobe left – curvature	-0.02 (-0.11-0.08)	.68	.95
Frontal lobe right – curvature	-0.07 (-0.17-0.02)	.08	.30
Occipital lobe left – curvature	-0.01 (-0.20-0.19)	.83	.95
Occipital lobe right – curvature	-0.002 (-0.11-0.10)	.74	.95
Parietal lobe left – curvature	0.01 (-0.08-0.11)	.72	.95
Parietal lobe right – curvature	-0.08 (-0.19-0.02)	.04	.30
Temporal lobe left – curvature	-0.01 (-0.11-0.08)	.80	.95
Temporal lobe right – curvature	-0.003 (-0.10-0.09)	.95	.97
Frontal lobe left – surface area	-0.04 (-0.21-0.14)	.41	.64
Frontal lobe right – surface area	-0.03 (-0.21-0.14)	.45	.65
Occipital lobe left – surface area	0.04 (-0.05-0.14)	.30	.51
Occipital lobe right – surface area	0.02 (-0.19-0.23)	.68	.83
Parietal lobe left – surface area	0.01 (-0.19-0.23)	.90	.90
Parietal lobe right – surface area	0.01 (-0.17-0.20)	.78	.86
Temporal lobe left – surface area	0.01 (-0.17-0.19)	.84	.88
Temporal lobe right – surface area	0.07 (-0.01-0.15)	.07	.20
Frontal lobe left – thickness	-0.01 (-0.22-0.20)	.83	.88
Frontal lobe right – thickness	-0.01 (-0.23-0.20)	.69	.83
Occipital lobe left – thickness	-0.02 (-0.12-0.07)	.56	.75
Occipital lobe right – thickness	-0.01 (-0.09-0.08)	.82	.88
Parietal lobe left – thickness	-0.03 (-0.11-0.05)	.48	.71

© 2023 Mckinnon K et al. *JAMA Network Open.*

**Cortical measure**	**Standardized β coefficient (95% confidence intervals)**	**Raw *p* value**	**BH corrected *p* value**
Parietal lobe right – thickness	-0.04 (-0.12-0.03)	.29	.59
Temporal lobe left – thickness	-0.06 (-0.14-0.03)	.18	.53
Temporal lobe right – thickness	-0.07 (-0.15-0.01)	.08	.45
Frontal lobe left – gyrification index	-0.01 (-0.12-0.10)	.65	.74
Frontal lobe right – gyrification index	<0.001 (-0.10-0.09)	.27	.40
Occipital lobe left – gyrification index	0.03 (-0.06-0.13)	.24	.39
Occipital lobe right – gyrification index	-0.01 (-0.13-0.10)	.07	.33
Parietal lobe left – gyrification index	0.02 (-0.11-0.11)	.94	.94
Parietal lobe right – gyrification index	0.03 (-0.12-0.18)	.14	.36
Temporal lobe left – gyrification index	0.003 (-0.12-0.13)	.86	.91
Temporal lobe right – gyrification index	-0.004 (-0.12-0.11)	.66	.74
Frontal lobe left – sulcal depth	0.05 (-0.19-0.28)	.09	.26
Frontal lobe right – sulcal depth	0.14 (0.03-0.25)	.001	.02
Occipital lobe left – sulcal depth	0.04 (-0.07-0.15)	.29	.47
Occipital lobe right – sulcal depth	0.04 (-0.07-0.15)	.31	.47
Parietal lobe left – sulcal depth	0.03 (-0.06-0.12)	.43	.55
Parietal lobe right – sulcal depth	-0.02 (-0.11-0.07)	.42	.55
Temporal lobe left – sulcal depth	0.01 (-0.09-0.11)	.55	.59
Temporal lobe right – sulcal depth	0.08 (-0.02-0.18)	.08	.26

Fully adjusted ridge regression model, including gestation at birth, maternal final educational qualification, gestation at MRI, the interaction term (where significant), birth weight z-score, birth head circumference z-score, sex, smoking in pregnancy, and breast milk at discharge. Corrected for false discovery rate.
BH = Benjamini-Hochberg correction.

© 2023 Mckinnon K et al. *JAMA Network Open.*

eTable 24. Cortical Measures: Association With Gestational Age, Paternal Education, and Interaction Effect (Fully Adjusted Ridge Regression Models)

Cortical measure	Standardized β coefficient (95% confidence intervals)	Raw *p* value	BH corrected *p* value
Gestation			
Frontal lobe left – curvature	0.03 (-0.07-0.13)	.47	.84
Frontal lobe right – curvature	0.02 (-0.08-0.11)	.68	.84
Occipital lobe left – curvature	-0.04 (-0.15-0.07)	.03	.37
Occipital lobe right – curvature	0.02 (-0.09-0.12)	.40	.84
Parietal lobe left – curvature	-0.09 (-0.19-0.02)	.046	.37
Parietal lobe right – curvature	-0.04 (-0.15-0.07)	.33	.84
Temporal lobe left – curvature	-0.02 (-0.13-0.08)	.59	.84
Temporal lobe right – curvature	-0.01 (-0.11-0.08)	.74	.84
Frontal lobe left – surface area	0.19 (0.11-0.27)	<.001	<.001
Frontal lobe right – surface area	0.18 (0.09-0.26)	<.001	<.001
Occipital lobe left – surface area	0.09 (-0.01-0.19)	.03	.06
Occipital lobe right – surface area	0.09 (-0.01-0.10	.02	.054
Parietal lobe left – surface area	0.17 (0.08-0.26)	<.001	<.001
Parietal lobe right – surface area	0.12 (0.03-0.22)	.003	.01
Temporal lobe left – surface area	0.14 (0.06-0.23)	<.001	.001
Temporal lobe right – surface area	0.13 (0.04-0.21)	.001	.004
Frontal lobe left – thickness	-0.06 (-0.37-0.25)	.01	.07
Frontal lobe right – thickness	-0.06 (-0.37-0.25)	.04	.14
Occipital lobe left – thickness	-0.01 (-0.12-0.08)	.89	.94
Occipital lobe right – thickness	-0.001 (-0.09-0.09)	.98	.98
Parietal lobe left – thickness	-0.02 (-0.11-0.08)	.69	.83
Parietal lobe right – thickness	0.01 (-0.09-0.12)	.78	.88
Temporal lobe left – thickness	0.04 (-0.07-0.15)	.02	.07
Temporal lobe right – thickness	0.03 (-0.07-0.14)	.44	.66
Frontal lobe left – gyrification index	0.03 (-0.10-0.15)	.14	.35
Frontal lobe right – gyrification index	<0.001 (-0.12-0.12)	.40	.56
Occipital lobe left – gyrification index	-0.03 (-0.15-0.10)	.49	.59
Occipital lobe right – gyrification index	-0.001 (-0.12-0.12)	.20	.35
Parietal lobe left – gyrification index	0.04 (-0.06-0.14)	.15	.35
Parietal lobe right – gyrification index	-0.06 (-0.32-0.19)	.01	.13
Temporal lobe left – gyrification index	0.02 (-0.12-0.16)	.18	.35
Temporal lobe right – gyrification index	0.02 (-0.12-0.15)	.23	.36
Frontal lobe left – sulcal depth	0.05 (-0.26-0.37)	.02	.24
Frontal lobe right – sulcal depth	-0.01 (-0.12-0.10)	.54	.73
Occipital lobe left – sulcal depth	0.02 (-0.06-0.10)	.54	.73
Occipital lobe right – sulcal depth	0.04 (-0.04-0.11)	.17	.62

© 2023 Mckinnon K et al. *JAMA Network Open.*

Cortical measure	Standardized β coefficient (95% confidence intervals)	Raw *p* value	BH corrected *p* value
Parietal lobe left – sulcal depth	-0.02 (-0.13-0.09)	.59	.73
Parietal lobe right – sulcal depth	-0.01 (-0.11-0.10)	.84	.89
Temporal lobe left – sulcal depth	-0.03 (-0.20-0.15)	.43	.73
Temporal lobe right – sulcal depth	0.01 (-0.09-0.11)	.75	.85
Interaction			
Frontal lobe left – thickness	-0.01 (-0.07-0.05)	.002	.03
Frontal lobe right – thickness	-0.01 (-0.07-0.05)	.01	.06
Parietal lobe right – gyrification index	-0.01 (-0.05-0.04)	.04	.25
Frontal lobe left – sulcal depth	0.01 (-0.05-0.06)	.03	.24
Temporal lobe left – sulcal depth	-0.01 (-0.04-0.03)	.049	.29
Paternal education			
Frontal lobe left – curvature	-0.01 (-0.10-0.07)	.71	.84
Frontal lobe right – curvature	0.01 (-0.07-0.09)	.83	.88
Occipital lobe left – curvature	0.01 (-0.08-0.10)	.58	.84
Occipital lobe right – curvature	0.02 (-0.07-0.10)	.35	.84
Parietal lobe left – curvature	0.03 (-0.06-0.11)	.49	.84
Parietal lobe right – curvature	-0.01 (-0.10-0.08)	.72	.84
Temporal lobe left – curvature	0.01 (-0.08-0.09)	.89	.89
Temporal lobe right – curvature	-0.03 (-0.11-0.05)	.43	.84
Frontal lobe left – surface area	-0.02 (-0.09-0.05)	.48	.65
Frontal lobe right – surface area	-0.03 (-0.10-0.04)	.44	.65
Occipital lobe left – surface area	-0.02 (-0.11-0.06)	.49	.65
Occipital lobe right – surface area	-0.02 (-0.10-0.07)	.67	.71
Parietal lobe left – surface area	-0.02 (-0.09-0.06)	.62	.71
Parietal lobe right – surface area	-0.01 (-0.08-0.07)	.81	.81
Temporal lobe left – surface area	0.02 (-0.05-0.09)	.54	.66
Temporal lobe right – surface area	0.03 (-0.03-0.10)	.29	.51
Frontal lobe left – thickness	-0.02 (-0.23-0.19)	.35	.66
Frontal lobe right – thickness	-0.03 (-0.24-0.18)	.25	.65
Occipital lobe left – thickness	-0.02 (-0.12-0.04)	.46	.66
Occipital lobe right – thickness	-0.04 (-0.11-0.04)	.26	.65
Parietal lobe left – thickness	-0.02 (-0.10-0.06)	.52	.66
Parietal lobe right – thickness	-0.02 (-0.11-0.06)	.52	.66
Temporal lobe left – thickness	-0.01 (-0.10-0.08)	.36	.66
Temporal lobe right – thickness	-0.04 (-0.12-0.05)	.29	.65
Frontal lobe left – gyrification index	0.003 (-0.10-0.11)	.83	.89
Frontal lobe right – gyrification index	-0.001 (-0.10-0.10)	.15	.35
Occipital lobe left – gyrification index	0.07 (-0.04-0.17)	.03	.25
Occipital lobe right – gyrification index	<0.001 (-0.10-0.10)	.62	.70

© 2023 Mckinnon K et al. *JAMA Network Open.*

Cortical measure	Standardized β coefficient (95% confidence intervals)	Raw *p* value	BH corrected *p* value
Parietal lobe left – gyrification index	0.03 (-0.05-0.11)	.20	.35
Parietal lobe right – gyrification index	0.03 (-0.15-0.20)	.19	.35
Temporal lobe left – gyrification index	0.01 (-0.10-0.13)	.43	.56
Temporal lobe right – gyrification index	-0.001 (-0.11-0.11)	.91	.91
Frontal lobe left – sulcal depth	0.01 (-0.21-0.23)	.61	.73
Frontal lobe right – sulcal depth	0.01 (-0.08-0.10)	.51	.73
Occipital lobe left – sulcal depth	-0.01 (-0.08-0.05)	.58	.73
Occipital lobe right – sulcal depth	-0.02 (-0.08-0.04)	.36	.73
Parietal lobe left – sulcal depth	-0.02 (-0.11-0.07)	.53	.73
Parietal lobe right – sulcal depth	-0.04 (-0.12-0.05)	.11	.47
Temporal lobe left – sulcal depth	-0.02 (-0.14-0.10)	.52	.73
Temporal lobe right – sulcal depth	0.001 (-0.08-0.08)	.99	.99

Fully adjusted ridge regression model, including gestation at birth, paternal final educational qualification, gestation at MRI, the interaction term (where significant), birth weight z-score, birth head circumference z-score, sex, smoking in pregnancy, and breast milk at discharge. Corrected for false discovery rate.
BH = Benjamini-Hochberg correction.

© 2023 Mckinnon K et al. *JAMA Network Open.*

eTable 25. Cortical Measures: Association With Gestational Age, Maternal Occupation, and Interaction Effect (Fully Adjusted Ridge Regression Models)

Cortical measure	Standardized β coefficient (95% confidence intervals)	Raw *p* value	BH corrected *p* value
Gestation			
Frontal lobe left – curvature	0.01 (-0.08-0.10)	.82	.89
Frontal lobe right – curvature	0.03 (-0.06-0.12)	.44	.79
Occipital lobe left – curvature	-0.02 (-0.36-0.32)	.56	.84
Occipital lobe right – curvature	0.01 (-0.10-0.11)	.64	.88
Parietal lobe left – curvature	0.01 (-0.32-0.34)	.89	.89
Parietal lobe right – curvature	-0.04 (-0.14-0.06)	.36	.73
Temporal lobe left – curvature	-0.01 (-0.10-0.08)	.76	.89
Temporal lobe right – curvature	-0.05 (-0.14-0.05)	.28	.63
Frontal lobe left – surface area	0.06 (-0.21-0.33)	.20	.42
Frontal lobe right – surface area	0.05 (-0.22-0.33)	.28	.46
Occipital lobe left – surface area	-0.02 (-0.34-0.31)	.73	.84
Occipital lobe right – surface area	-0.01 (-0.34-0.32)	.84	.88
Parietal lobe left – surface area	0.05 (-0.23-0.34)	.27	.46
Parietal lobe right – surface area	0.03 (-0.26-0.32)	.53	.75
Temporal lobe left – surface area	0.03 (-0.26-0.32)	.56	.75
Temporal lobe right – surface area	0.10 (0.02-0.18)	.01	.11
Frontal lobe left – thickness	-0.06 (-0.39-0.28)	.04	.17
Frontal lobe right – thickness	-0.05 (-0.39-0.29)	.10	.19
Occipital lobe left – thickness	-0.02 (-0.12-0.07)	.58	.68
Occipital lobe right – thickness	-0.01 (-0.10-0.08)	.88	.88
Parietal lobe left – thickness	-0.02 (-0.11-0.07)	.61	.68
Parietal lobe right – thickness	0.02 (-0.08-0.11)	.68	.72
Temporal lobe left – thickness	0.07 (-0.03-0.16)	.12	.19
Temporal lobe right – thickness	0.04 (-0.06-0.13)	.38	.49
Frontal lobe left – gyrification index	0.02 (-0.31-0.35)	.26	.40
Frontal lobe right – gyrification index	0.002 (-0.11-0.12)	.41	.53
Occipital lobe left – gyrification index	0.08 (-0.28-0.44)	.10	.23
Occipital lobe right – gyrification index	-0.01 (-0.13-0.10)	.23	.39
Parietal lobe left – gyrification index	0.05 (-0.12-0.21)	.10	.23
Parietal lobe right – gyrification index	-0.05 (-0.32-0.22)	.01	.051
Temporal lobe left – gyrification index	0.01 (-0.11-0.14)	.14	.28
Temporal lobe right – gyrification index	0.02 (-0.10-0.14)	.23	.39
Frontal lobe left – sulcal depth	0.06 (-0.05-0.17)	.08	.36
Frontal lobe right – sulcal depth	-0.04 (-0.15-0.06)	.15	.36
Occipital lobe left – sulcal depth	-0.04 (-0.15-0.06)	.32	.46
Occipital lobe right – sulcal depth	-0.01 (-0.12-0.09)	.74	.76

© 2023 Mckinnon K et al. *JAMA Network Open.*

Cortical measure	Standardized β coefficient (95% confidence intervals)	Raw *p* value	BH corrected *p* value
Parietal lobe left – sulcal depth	-0.02 (-0.11-0.08)	.65	.76
Parietal lobe right – sulcal depth	-0.01 (-0.11-0.09)	.76	.76
Temporal lobe left – sulcal depth	-0.09 (-0.19-0.01)	.02	.36
Temporal lobe right – sulcal depth	-0.06 (-0.16-0.04)	.16	.36
Interaction			
Occipital lobe left – curvature	-0.01 (-0.05-0.03)	<.001	.005
Parietal lobe left – curvature	-0.01 (-0.05-0.03)	.02	.21
Frontal lobe left – surface area	0.01 (-0.02-0.05)	.02	.11
Frontal lobe right – surface area	0.01 (-0.02-0.05)	.02	.11
Occipital lobe left – surface area	0.01 (-0.03-0.05)	.04	.11
Occipital lobe right – surface area	0.01 (-0.03-0.05)	.03	.11
Parietal lobe left – surface area	0.01 (-0.02-0.05)	.01	.11
Parietal lobe right – surface area	0.01 (-0.03-0.05)	.47	.72
Temporal lobe left – surface area	0.01 (-0.02-0.05)	.03	.11
Frontal lobe left – thickness	-0.01 (-0.05-0.04)	.001	.01
Frontal lobe right – thickness	-0.01 (-0.05-0.04)	.001	.01
Frontal lobe left – gyrification index	0.003 (-0.04-0.05)	.03	.09
Occipital lobe left – gyrification index	-0.01 (-0.06-0.03)	<.001	<.001
Parietal lobe left – gyrification index	0.01 (-0.02-0.03)	.01	.051
Parietal lobe right – gyrification index	-0.003 (-0.04-0.03)	.03	.09
Maternal occupation			
Frontal lobe left – curvature	-0.005 (-0.07-0.06)	.86	.89
Frontal lobe right – curvature	-0.03 (-0.09-0.03)	.27	.63
Occipital lobe left – curvature	-0.03 (-0.18-0.12)	.19	.63
Occipital lobe right – curvature	-0.01 (-0.08-0.06)	.19	.63
Parietal lobe left – curvature	-0.03 (-0.18-0.11)	.25	.63
Parietal lobe right – curvature	-0.01 (-0.08-0.05)	.54	.84
Temporal lobe left – curvature	0.01 (-0.05-0.07)	.72	.89
Temporal lobe right – curvature	-0.03 (-0.10-0.03)	.25	.63
Frontal lobe left – surface area	-0.01 (-0.13-0.11)	.76	.84
Frontal lobe right – surface area	-0.01 (-0.14-0.11)	.60	.77
Occipital lobe left – surface area	0.03 (-0.11-0.18)	.23	.45
Occipital lobe right – surface area	0.04 (-0.11-0.18)	.17	.40
Parietal lobe left – surface area	0.004 (-0.12-0.13)	.88	.88
Parietal lobe right – surface area	0.02 (-0.11-0.15)	.03	.11
Temporal lobe left – surface area	0.01 (-0.11-0.14)	.65	.79
Temporal lobe right – surface area	0.05 (-0.001-0.10)	.04	.11
Frontal lobe left – thickness	-0.03 (-0.18-0.11)	.09	.19
Frontal lobe right – thickness	-0.03 (-0.18-0.12)	.14	.20
Occipital lobe left – thickness	-0.07 (-0.12- -0.01)	.02	.10

© 2023 Mckinnon K et al. *JAMA Network Open.*

Cortical measure	Standardized β coefficient (95% confidence intervals)	Raw *p* value	BH corrected *p* value
Occipital lobe right – thickness	-0.05 (-0.09- -0.001)	.09	.19
Parietal lobe left – thickness	-0.05 (-0.10- -0.002)	.07	.19
Parietal lobe right – thickness	-0.05 (-0.11-0.004)	.06	.19
Temporal lobe left – thickness	-0.04 (-0.10-0.02)	.14	.20
Temporal lobe right – thickness	-0.04 (-0.10-0.02)	.11	.19
Frontal lobe left – gyrification index	0.01 (-0.14-0.15)	.46	.53
Frontal lobe right – gyrification index	-0.001 (-0.06-0.06)	.72	.72
Occipital lobe left – gyrification index	-0.17 (-0.33- -0.01)	.01	.051
Occipital lobe right – gyrification index	-0.02 (-0.09-0.05)	.01	.051
Parietal lobe left – gyrification index	0.02 (-0.04-0.08)	.43	.53
Parietal lobe right – gyrification index	0.01 (-0.10-0.12)	.47	.53
Temporal lobe left – gyrification index	-0.01 (-0.08-0.07)	.34	.49
Temporal lobe right – gyrification index	0.01 (-0.07-0.08)	.60	.63
Frontal lobe left – sulcal depth	0.01 (-0.07-0.08)	.76	.76
Frontal lobe right – sulcal depth	0.03 (-0.04-0.10)	.09	.36
Occipital lobe left – sulcal depth	-0.05 (-0.11-0.02)	.12	.36
Occipital lobe right – sulcal depth	-0.03 (-0.10-0.04)	.26	.46
Parietal lobe left – sulcal depth	-0.03 (-0.08-0.02)	.25	.46
Parietal lobe right – sulcal depth	-0.03 (-0.09-0.03)	.15	.36
Temporal lobe left – sulcal depth	-0.02 (-0.08-0.05)	.53	.71
Temporal lobe right – sulcal depth	0.03 (-0.03-0.10)	.29	.46

Fully adjusted ridge regression model, including gestation at birth, maternal occupation, gestation at MRI, the interaction term (where significant), birth weight z-score, birth head circumference z-score, sex, smoking in pregnancy, and breast milk at discharge. Corrected for false discovery rate.
BH = Benjamini-Hochberg correction.

© 2023 Mckinnon K et al. *JAMA Network Open.*

eTable 26. Cortical Measures: Association With Gestational Age, Paternal Occupation, and Interaction Effect (Fully Adjusted Ridge Regression Models)

Cortical measure	Standardized β coefficient (95% confidence intervals)	Raw *p* value	BH corrected *p* value
Gestation			
Frontal lobe left – curvature	0.003 (-0.09-0.10)	.95	.99
Frontal lobe right – curvature	0.02 (-0.06-0.11)	.56	.89
Occipital lobe left – curvature	-0.03 (-0.41-0.35)	.23	.55
Occipital lobe right – curvature	0.01 (-0.10-0.12)	.67	.89
Parietal lobe left – curvature	-0.06 (-0.15-0.04)	.18	.55
Parietal lobe right – curvature	-0.001 (-0.41-0.41)	.99	.99
Temporal lobe left – curvature	-0.02 (-0.11-0.08)	.69	.89
Temporal lobe right – curvature	-0.04 (-0.14-0.06)	.34	.61
Frontal lobe left – surface area	0.16 (0.08-0.24)	<.001	.001
Frontal lobe right – surface area	0.14 (0.06-0.22)	<.001	.002
Occipital lobe left – surface area	0.04 (-0.05-0.14)	.30	.32
Occipital lobe right – surface area	0.05 (-0.05-0.14)	.27	.32
Parietal lobe left – surface area	0.14 (0.05-0.22)	<.001	.002
Parietal lobe right – surface area	0.10 (0.01-0.18)	.01	.04
Temporal lobe left – surface area	0.11 (0.02-0.19)	.01	.03
Temporal lobe right – surface area	0.09 (0.01-0.18)	.01	.04
Frontal lobe left – thickness	-0.06 (-0.41-0.29)	.01	.12
Frontal lobe right – thickness	-0.05 (-0.41-0.31)	.04	.18
Occipital lobe left – thickness	-0.01 (-0.11-0.08)	.72	.76
Occipital lobe right – thickness	0.01 (-0.09-0.12)	.34	.71
Parietal lobe left – thickness	-0.02 (-0.12-0.08)	.66	.76
Parietal lobe right – thickness	0.02 (-0.08-0.12)	.69	.76
Temporal lobe left – thickness	0.06 (-0.03-0.16)	.15	.52
Temporal lobe right – thickness	0.03 (-0.07-0.13)	.43	.71
Frontal lobe left – gyrification index	0.03 (-0.08-0.14)	.12	.31
Frontal lobe right – gyrification index	0.003 (-0.11-0.12)	.36	.53
Occipital lobe left – gyrification index	0.003 (-0.41-0.42)	.91	.91
Occipital lobe right – gyrification index	-0.002 (-0.12-0.11)	.22	.36
Parietal lobe left – gyrification index	0.03 (-0.08-0.15)	.06	.17
Parietal lobe right – gyrification index	-0.06 (-0.35-0.24)	.01	.08
Temporal lobe left – gyrification index	0.05 (-0.07-0.17)	.17	.35
Temporal lobe right – gyrification index	0.02 (-0.10-0.14)	.21	.36
Frontal lobe left – sulcal depth	0.03 (-0.39-0.46)	.19	.63
Frontal lobe right – sulcal depth	-0.04 (-0.14-0.07)	.22	.63
Occipital lobe left – sulcal depth	-0.04 (-0.15-0.06)	.33	.65
Occipital lobe right – sulcal depth	-0.01 (-0.12-0.10)	.77	.82

© 2023 Mckinnon K et al. *JAMA Network Open.*

Cortical measure	Standardized β coefficient (95% confidence intervals)	Raw *p* value	BH corrected *p* value
Parietal lobe left – sulcal depth	-0.01 (-0.11-0.10)	.77	.82
Parietal lobe right – sulcal depth	-0.004 (-0.11-0.10)	.89	.89
Temporal lobe left – sulcal depth	-0.11 (-0.21- -0.01)	.01	.25
Temporal lobe right – sulcal depth	-0.04 (-0.14-0.06)	.34	.65
Interaction			
Occipital lobe left – curvature	-0.01 (-0.06-0.04)	.003	.052
Parietal lobe right – curvature	-0.01 (-0.06-0.05)	.04	.23
Frontal lobe left – thickness	-0.005 (-0.05-0.04)	.01	.12
Frontal lobe right – thickness	-0.01 (-0.05-0.04)	.02	.12
Occipital lobe left – gyrification index	-0.01 (-0.06-0.05)	.03	.15
Parietal lobe right – gyrification index	-0.004 (-0.04-0.03)	.048	.17
Frontal lobe left – sulcal depth	0.004 (-0.05-0.06)	.04	.33
Paternal occupation			
Frontal lobe left – curvature	0.01 (-0.06-0.07)	.86	.97
Frontal lobe right – curvature	0.01 (-0.05-0.08)	.65	.89
Occipital lobe left – curvature	-0.04 (-0.23-0.16)	.15	.55
Occipital lobe right – curvature	-0.02 (-0.09-0.06)	.21	.55
Parietal lobe left – curvature	-0.06 (-0.13-0.005)	.04	.23
Parietal lobe right – curvature	-0.03 (-0.24-0.18)	.25	.55
Temporal lobe left – curvature	0.01 (-0.06-0.07)	.84	.97
Temporal lobe right – curvature	-0.03 (-0.10-0.03)	.28	.55
Frontal lobe left – surface area	0.01 (-0.04-0.07)	.64	.64
Frontal lobe right – surface area	0.03 (-0.03-0.09)	.28	.32
Occipital lobe left – surface area	0.05 (-0.02-0.11)	.15	.21
Occipital lobe right – surface area	0.04 (-0.03-0.11)	.25	.32
Parietal lobe left – surface area	0.05 (-0.01-0.11)	.07	.16
Parietal lobe right – surface area	0.05 (-0.02-0.11)	.11	.21
Temporal lobe left – surface area	0.04 (-0.02-0.10)	.12	.21
Temporal lobe right – surface area	0.04 (-0.02-0.10)	.13	.21
Frontal lobe left – thickness	-0.01 (-0.19-0.17)	.60	.76
Frontal lobe right – thickness	-0.03 (-0.21-0.15)	.25	.71
Occipital lobe left – thickness	-0.03 (-0.09-0.03)	.35	.71
Occipital lobe right – thickness	-0.01 (-0.08-0.06)	.55	.76
Parietal lobe left – thickness	-0.01 (-0.08-0.06)	.79	.79
Parietal lobe right – thickness	-0.03 (-0.10-0.04)	.36	.71
Temporal lobe left – thickness	-0.03 (-0.09-0.04)	.43	.71
Temporal lobe right – thickness	-0.02 (-0.09-0.05)	.62	.76
Frontal lobe left – gyrification index	-0.002 (-0.08-0.07)	.89	.91
Frontal lobe right – gyrification index	-0.003 (-0.07-0.06)	.18	.35
Occipital lobe left – gyrification index	-0.09 (-0.31-0.12)	<.001	.01

© 2023 Mckinnon K et al. *JAMA Network Open.*

Cortical measure	Standardized β coefficient (95% confidence intervals)	Raw *p* value	BH corrected *p* value
Occipital lobe right – gyrification index	<0.001 (-0.06-0.06)	.91	.91
Parietal lobe left – gyrification index	-0.004 (-0.08-0.07)	.79	.91
Parietal lobe right – gyrification index	0.01 (-0.13-0.15)	.68	.87
Temporal lobe left – gyrification index	-0.07 (-0.14-0.004)	.02	.10
Temporal lobe right – gyrification index	-0.01 (-0.09-0.07)	.57	.78
Frontal lobe left – sulcal depth	0.04 (-0.18-0.26)	.07	.36
Frontal lobe right – sulcal depth	0.01 (-0.06-0.09)	.57	.74
Occipital lobe left – sulcal depth	-0.03 (-0.10-0.05)	.44	.68
Occipital lobe right – sulcal depth	-0.01 (-0.09-0.06)	.66	.80
Parietal lobe left – sulcal depth	-0.03 (-0.08-0.02)	.30	.65
Parietal lobe right – sulcal depth	-0.04 (-0.11-0.03)	.08	.36
Temporal lobe left – sulcal depth	0.02 (-0.05-0.09)	.48	.69
Temporal lobe right – sulcal depth	0.02 (-0.05-0.09)	.44	.68

Fully adjusted ridge regression model, including gestation at birth, paternal occupation, gestation at MRI, the interaction term (where significant), birth weight z-score, birth head circumference z-score, sex, smoking in pregnancy, and breast milk at discharge. Corrected for false discovery rate.
BH = Benjamini-Hochberg correction.

© 2023 Mckinnon K et al. *JAMA Network Open.*

eTable 27. Cortical Measures: Association With Gestational Age, Subjective Socioeconomic Status, and Interaction Effect (Fully Adjusted Ridge Regression Models)

Cortical measure	Standardized β coefficient (95% confidence intervals)	Raw *p* value	BH corrected *p* value
Gestation			
Frontal lobe left – curvature	0.03 (-0.07-0.13)	.53	.83
Frontal lobe right – curvature	0.09 (-0.34-0.52)	.07	.33
Occipital lobe left – curvature	-0.04 (-0.51-0.44)	.23	.52
Occipital lobe right – curvature	-0.01 (-0.12-0.11)	.69	.83
Parietal lobe left – curvature	-0.06 (-0.16-0.05)	.19	.52
Parietal lobe right – curvature	-0.05 (-0.16-0.06)	.25	.52
Temporal lobe left – curvature	0.001 (-0.10-0.10)	.98	.98
Temporal lobe right – curvature	0.02 (-0.44-0.48)	.54	.83
Frontal lobe left – surface area	0.14 (0.06-0.22)	<.001	.01
Frontal lobe right – surface area	0.13 (0.05-0.21)	.001	.01
Occipital lobe left – surface area	0.03 (-0.07-0.14)	.44	.76
Occipital lobe right – surface area	0.03 (-0.07-0.14)	.46	.76
Parietal lobe left – surface area	0.14 (0.05-0.22)	.001	.01
Parietal lobe right – surface area	0.10 (0.01-0.19)	.01	.052
Temporal lobe left – surface area	0.09 (0.005-0.18)	.02	.08
Temporal lobe right – surface area	0.09 (0.003-0.17)	.03	.08
Frontal lobe left – thickness	-0.04 (-0.44-0.35)	.06	.33
Frontal lobe right – thickness	-0.05 (-0.45-0.35)	.12	.44
Occipital lobe left – thickness	-0.004 (-0.11-0.10)	.92	.98
Occipital lobe right – thickness	0.02 (-0.09-0.13)	.64	.92
Parietal lobe left – thickness	0.01 (-0.10-0.13)	.47	.89
Parietal lobe right – thickness	0.03 (-0.08-0.14)	.55	.89
Temporal lobe left – thickness	0.07 (-0.03-0.18)	.11	.44
Temporal lobe right – thickness	0.06 (-0.05-0.17)	.17	.50
Frontal lobe left – gyrification index	0.02 (-0.11-0.15)	.46	.65
Frontal lobe right – gyrification index	0.01 (-0.13-0.14)	.46	.65
Occipital lobe left – gyrification index	-0.03 (-0.17-0.11)	.41	.65
Occipital lobe right – gyrification index	-0.001 (-0.13-0.13)	.21	.60
Parietal lobe left – gyrification index	0.02 (-0.10-0.14)	.11	.60
Parietal lobe right – gyrification index	-0.02 (-0.31-0.26)	.02	.26
Temporal lobe left – gyrification index	0.02 (-0.12-0.17)	.19	.60
Temporal lobe right – gyrification index	0.02 (-0.11-0.15)	.28	.65
Frontal lobe left – sulcal depth	0.05 (-0.07-0.16)	.16	.52
Frontal lobe right – sulcal depth	-0.04 (-0.16-0.08)	.15	.52
Occipital lobe left – sulcal depth	-0.05 (-0.16-0.07)	.28	.64

© 2023 Mckinnon K et al. *JAMA Network Open.*

Cortical measure	Standardized β coefficient (95% confidence intervals)	Raw *p* value	BH corrected *p* value
Occipital lobe right – sulcal depth	-0.01 (-0.13-0.10)	.75	.95
Parietal lobe left – sulcal depth	-0.01 (-0.12-0.11)	.75	.95
Parietal lobe right – sulcal depth	-0.002 (-0.12-0.11)	.93	.95
Temporal lobe left – sulcal depth	-0.10 (-0.20-0.01)	.04	.52
Temporal lobe right – sulcal depth	-0.05 (-0.15-0.06)	.32	.64
Interaction			
Frontal lobe right – curvature	-0.001 (-0.01-0.004)	.04	.26
Occipital lobe left – curvature	-0.001 (-0.01-0.01)	.002	.03
Temporal lobe right – curvature	-0.001 (-0.01-0.005)	.02	.22
Frontal lobe left – thickness	-0.001 (-0.01-0.004)	.01	.26
Frontal lobe right – thickness	-0.001 (-0.01-0.004)	.03	.31
Parietal lobe right – gyrification index	-0.001 (-0.004-0.003)	.03	.29
Subjective socioeconomic status			
Frontal lobe left – curvature	-0.01 (-0.01-0.004)	.20	.52
Frontal lobe right – curvature	0.002 (-0.02-0.02)	.70	.83
Occipital lobe left – curvature	-0.01 (-0.03-0.02)	.12	.44
Occipital lobe right – curvature	-0.001 (-0.01-0.01)	.92	.98
Parietal lobe left – curvature	-0.002 (-0.01-0.01)	.64	.83
Parietal lobe right – curvature	-0.002 (-0.01-0.01)	.66	.83
Temporal lobe left – curvature	-0.001 (-0.01-0.01)	.88	.98
Temporal lobe right – curvature	-0.003 (-0.02-0.02)	.42	.80
Frontal lobe left – surface area	0.002 (-0.01-0.01)	.55	.76
Frontal lobe right – surface area	0.003 (-0.004-0.01)	.41	.76
Occipital lobe left – surface area	0.003 (-0.01-0.01)	.53	.76
Occipital lobe right – surface area	0.002 (-0.01-0.01)	.57	.76
Parietal lobe left – surface area	0.001 (-0.01-0.01)	.73	.90
Parietal lobe right – surface area	0.001 (-0.01-0.01)	.79	.90
Temporal lobe left – surface area	0.001 (-0.01-0.01)	.85	.91
Temporal lobe right – surface area	<0.001 (-0.01-0.01)	.91	.91
Frontal lobe left – thickness	-0.001 (-0.02-0.02)	.66	.92
Frontal lobe right – thickness	-0.002 (0.02-0.01)	.53	.89
Occipital lobe left – thickness	-0.001 (-0.01-0.01)	.78	.98
Occipital lobe right – thickness	-0.001 (-0.01-0.01)	.98	.98
Parietal lobe left – thickness	-0.001 (-0.01-0.01)	.92	.98
Parietal lobe right – thickness	-0.001 (-0.01-0.01)	.93	.98
Temporal lobe left – thickness	-0.003 (-0.01-0.01)	.42	.89
Temporal lobe right – thickness	-0.004 (-0.01-0.01)	.33	.85
Frontal lobe left – gyrification index	0.002 (-0.01-0.01)	.39	.65
Frontal lobe right – gyrification index	0.001 (-0.01-0.01)	.35	.65
Occipital lobe left – gyrification index	-0.001 (-0.01-0.01)	.85	.91

© 2023 Mckinnon K et al. *JAMA Network Open.*

Cortical measure	Standardized β coefficient (95% confidence intervals)	Raw *p* value	BH corrected *p* value
Occipital lobe right – gyrification index	<0.001 (-0.01-0.01)	.20	.60
Parietal lobe left – gyrification index	<0.001 (-0.01-0.01)	.91	.91
Parietal lobe right – gyrification index	<0.001 (-0.01-0.01)	.82	.91
Temporal lobe left – gyrification index	0.001 (-0.01-0.01)	.59	.71
Temporal lobe right – gyrification index	0.001 (-0.01-0.01)	.56	.71
Frontal lobe left – sulcal depth	0.001 (-0.01-0.01)	.84	.95
Frontal lobe right – sulcal depth	<0.001 (-0.01-0.01)	.91	.95
Occipital lobe left – sulcal depth	-0.003 (-0.01-0.01)	.39	.69
Occipital lobe right – sulcal depth	<0.001 (-0.01-0.01)	.95	.95
Parietal lobe left – sulcal depth	0.005 (-0.004-0.01)	.20	.54
Parietal lobe right – sulcal depth	0.002 (-0.01-0.01)	.52	.83
Temporal lobe left – sulcal depth	-0.01 (-0.01-0.003)	.14	.52
Temporal lobe right – sulcal depth	-0.01 (-0.01-0.003)	.16	.52

Fully adjusted ridge regression model, including gestation at birth, subjective socioeconomic status, gestation at MRI, the interaction term (where significant), birth weight z-score, birth head circumference z-score, sex, smoking in pregnancy, and breast milk at discharge. Corrected for false discovery rate.
BH = Benjamini-Hochberg correction.

© 2023 Mckinnon K et al. *JAMA Network Open.*

eReferences

1. Scottish National Statistics. *SIMD - Scottish Index of Multiple Deprivation: SIMD16 Technical Notes*. Scottish National Statistics; 2016:1-69.

2. WHO. *World Health Organization Quality of Life Assessment: WHOQOL-BREF*. World Health Organization; 1996.

3. Boardman JP, Hall J, Thrippleton MJ, et al. Impact of preterm birth on brain development and long-term outcome: protocol for a cohort study in Scotland. *Bmj Open*. 2020;10:e035854. doi:10.1136/bmjopen-2019-035854

© 2023 Mckinnon K et al. *JAMA Network Open.*
