# Supplementary material for: Association of Preterm Birth and Socioeconomic Status With Neonatal Brain Structure
Source: JAMA Netw Open. 2023 May 31;6(5):e2316067. doi: 10.1001/jamanetworkopen.2023.16067 (PMC10233421; doi:10.1001/jamanetworkopen.2023.16067)
Supplement: Supplement 2. — Data Sharing Statement [file jamanetwopen-e2316067-s002.pdf]

Data Sharing Statement

Mckinnon. Association of Preterm Birth and Socioeconomic Status With Neonatal Brain Structure. *JAMA Netw Open*. Published May 31, 2023.
doi:10.1001/jamanetworkopen.2023.16067

Data
Data available: Yes
Data types: Deidentified participant data
How to access data: Yes, subject to a data access agreement:
https://www.tebc.ed.ac.uk/2019/12/data-access-and-collaboration/
When available: With publication

Supporting Documents
Document types: Statistical/analytic code
How to access documents: Yes, subject to a data access agreement:
https://www.tebc.ed.ac.uk/2019/12/data-access-and-collaboration/
When available: With publication

Additional Information
Who can access the data: Researchers whose proposed use of the data has been approved.
Types of analyses: For a specified purpose.
Mechanisms of data availability: With a signed data access agreement
Any additional restrictions: Terms and conditions of the data access agreement
https://www.tebc.ed.ac.uk/2019/12/data-access-and-collaboration/
